# The association between informal caregiving and behavioral risk factors: a cross-sectional study

**DOI:** 10.1007/s00038-020-01402-6

**Published:** 2020-06-09

**Authors:** Sophie Gottschalk, Hans-Helmut König, Christian Brettschneider

**Affiliations:** Department of Health Economics and Health Services Research, Hamburg Center for Health Economics, University Medical Center Hamburg-Eppendorf, Hamburg, Germany

**Keywords:** Informal care, Behavioral risk factors, Alcohol consumption, Tobacco use, Physical activity, Obesity

## Abstract

**Objectives:**

This study aimed to compare informal caregivers/dementia caregivers to non-caregivers regarding alcohol consumption, smoking behavior, obesity, and insufficient physical activity and to identify caregiving-related factors (caregiving intensity, length of caregiving, relationship to the care recipient, and type of caregiving task) which are associated with behavioral risk factors in caregivers/dementia caregivers.

**Methods:**

Using cross-sectional data from the Behavioral Risk Factor Surveillance System, we performed the statistical analyses applying logistic regression models and accounted for confounding using the entropy balancing approach.

**Results:**

For caregivers (*n* = 12,044), the odds of overweight/obesity and smoking were higher (OR = 1.14/1.34, *p* < 0.05) and the odds of binge drinking and insufficient physical activity were lower (OR = 0.86/0.83, *p* < 0.05) than for non-caregivers (*n* = 45,925). For dementia caregivers, results point in the same direction. Caregiving-related variables tend to influence the likelihood of behavioral risk factors, but depending on the kind of factor considered, in different directions.

**Conclusions:**

Being a caregiver is associated with risky and health-promoting behavior. However, the effects are relatively low. Future studies should study potential pathways between caregiving characteristics, psychological impacts of caregiving, health behavior, and mental or physical health.

**Electronic supplementary material:**

The online version of this article (10.1007/s00038-020-01402-6) contains supplementary material, which is available to authorized users.

## Introduction

In the United States, an estimated number of 43.5 million adults provides unpaid care (National Alliance for Caregiving (NAC) and AARP Public Policy Institute 2015). The demand for so-called informal caregivers (CG) is probably rising as the number of potential care recipients (population aged 65 plus) is projected to increase (United Nations 2017).

The effects of informal caregiving on the CG have received considerable attention. Physical and mental health were primarily found to be negatively associated with informal caregiving (Bauer and Sousa-Poza 2015). However, more recent findings show that caregiving is also associated with benefits, such as personal growth and meaning of life, and that most of the earlier studies drew their conclusions from convenience samples prone to selection bias (Campos et al. 2019; Roth et al. 2015).

The pathway of the association between caregiver status and physical health outcomes is to date not completely clarified. Bauer and Sousa-Poza (2015) suggest three possible dynamics. First, due to the high physical demand of caregiving, CG may develop chronic diseases, for example, of the musculoskeletal system. Second, they hypothesize that caregiving increases chronic stress which might lead to physical health outcomes like hypertension or cardiovascular diseases. Finally, the negligence of health promoting lifestyles could be responsible for lower physical health in CG compared to non-CG (NCG). Health behavior plays an important role in the development or prevention of negative health outcomes, chronic diseases, and mortality (Loef and Walach 2012; World Health Organization 2017). Furthermore, different studies report that being a caregiver is associated with behavioral risk factors like obesity or tobacco use, soda and fast food consumption (Hoffman et al. 2012; Reeves et al. 2012). Since caregiving can be a burdensome experience CG have to cope with (van der Lee et al. 2014), it can be hypothesized that risky health behavior functions as coping mechanism.

Dementia caregivers (DCG) are a CG group who experiences high psychological stress, resulting in negative effects on physical health (Pinquart and Sörensen 2003, 2007; Schoenmakers et al. 2010) and lower quality of life compared to CG of other neurological diseases (Karg et al. 2018). Dealing with the symptoms of progressive cognitive decline in people with dementia poses a particular challenge for DCG (van der Lee et al. 2014). Hence, DCG are a vulnerable group that may differ from other CG in terms of their health behavior.

This study has two aims. First, we address the hypothesis that worse physical health in CG is attributable to health behavior by comparing NCG to CG and to DCG regarding selected behavioral risk factors (alcohol consumption, smoking behavior, overweight/obesity, insufficient physical activity). Although previous studies had comparable goals, they were either restricted to the baby boom generation (Hoffman et al. 2012) or used a smaller sample, different statistical methods, and focused exclusively on women (Reeves et al. 2012). Second, we assess the relationship between caregiving-related variables (caregiving intensity, length of caregiving, relationship to care recipient, and caregiving tasks) and behavioral risk factors within the group of CG and DCG.

## Methods

### Study design

In this cross-sectional study, we used data from the 2017 wave of the Behavioral Risk Factor Surveillance System (BRFSS) of the Center for Disease Control. In the BRFSS, data from US residents in 50 states, the District of Columbia and in three US territories are annually collected via landline and cellular telephone interviews. The questionnaire consists of a core component, optional modules, and state-added questions. In 2017, the optional caregiver module was implemented in 12 States (Alaska, Hawaii, New Mexico, Oregon, Rhode Island, Kansas, Michigan, New Jersey, New York, Oklahoma, Utah, and Maryland). Hence, the sample consists of 66,601 individuals.

### Dependent variables: health-related risk factors

We considered four health-related risk factors: overweight/obesity, smoking behavior, alcohol consumption, and insufficient physical activity. Body weight was classified by the Body Mass Index (BMI) according to the classification scheme of the World Health Organization. People were assigned to one of the four groups: underweight (BMI < 18.5), normal weight (BMI 18.5–24), overweight (BMI 25–29), and obese (BMI 30+). People were classified according to their self-reported smoking behavior either as non-smoker, smoking some days, or daily smoker. To capture risky alcohol consumption, we used classifications based on the *2015*–*2020 Dietary Guidelines for Americans* (U.S. Department of Health and Human Services and U.S. Department of Agriculture December 2015). A person was considered as binge drinker if 5 or more (men) or 4 or more (women) alcoholic drinks on one occasion were consumed within the last 30 days. One drink is equivalent to a 12-ounce beer, a 5-ounce glass of wine, or a drink with one shot of liquor. Furthermore, based on the average number of drinks per occasion, alcohol consumption was divided into moderate or no drinker (≤ 1 drink for women, ≤ 2 drinks for men), more than moderate drinker (2–3 drinks for women or 3–4 drinks for men), and high-risk drinker (≥ 4 drinks for women and ≥ 5 drinks for men). Physical activity was classified based on the number of minutes of activity per week, in accordance to the guidelines of the Physical Activity Guidelines Advisory Committee (2018). The categories included highly active (at least 300 min or vigorous equivalent), active (150–300 min or vigorous equivalent), insufficiently active (11–149 min), and inactive (no physical activity other than regular job).

### Key independent variables: caregiving-related variables

Caregiver status (yes/no) was derived from the question ‘During the past 30 days, did you provide regular care or assistance to a friend or family member who has a health problem or disability?’. People whose care recipient died within the past 30 days were coded as missing because they were not included in subsequent caregiving-related questions. The variable ‘NCG versus DCG’ was derived from the caregiver status and the question ‘What is the main health problem, long-term illness, or disability that the person you care for has?’ If people reported that the main problem is dementia or another cognitive impairment disorder, people were categorized as DCG. As further caregiving-related variables, caregiving intensity (up to 8 h, 9–39 h, ≥ 40 h per week), length of the caregiving period (< 1 month, 1 month to < 2 years, ≥ 2 years), relationship to caregiving recipient (friend/non-relative, parent/parent-in-law/grandparent, child/grandchild, spouse/partner, sibling(-in-law), other relative), and type of caregiving tasks (household tasks only, personal care only, both, other [not further specified] care tasks) were considered.

### Statistical analysis

We initially compared the group of NCG to the group of CG as well as to the group of DCG with respect to their behavioral risk factors. All groups showed some differences regarding their sociodemographic characteristics. Consequently, we chose the entropy balancing approach (Hainmueller 2012) to achieve comparability between groups by creating a quasi-experiment. In a recent comparative study, this method performed best compared to other matching methods for balancing covariates (inverse probability treatment weighting, propensity score matching) (Matschinger et al. 2020). Entropy balancing was performed for each risk factor separately by balancing the characteristics of the NCG group to the CG or DCG group. In each balancing, the distributions of age, sex, race, highest educational degree, employment status, number of children, marital status, as well as days in poor mental and physical health were made comparable up to the third moment (mean, variance, skewness). In addition, for each risk behavior, the distribution of the other risk factors was balanced, except for overweight and physical inactivity where the respective other risk factor was not included due to high correlation between these two variables. Risk factors were either dichotomized [BMI > 25 (overweight/obesity) vs. BMI ≤ 25; binge drinker yes vs. no (≥ 4 for women or ≥ 5 drinks for men on at least one occasion in the last 30 days); each and someday smoker (smoker) vs. non-smoker; activity < 150 min per week vs. activity ≥ 150 min] in a binomial logistic regression model or used as assessed in multinomial regression models. Furthermore, entropy balancing between the CG and the NCG group was performed for each age category (18–49; 50–64; 65 +) in order to analyze the differences between CG and NCG in different age strata using binominal logistic regressions. Due to limited sample size to successfully balance the groups for all constraints, we did not perform age-stratified analyses for DCG.

In a second step, we assessed the relationship between caregiving-related variables (caregiving intensity, length of caregiving, relationship to care recipient, and caregiving tasks) and behavioral risk factors within the group of CG and DCG using logistic regression models. Since some subgroups within the caregiving-related variables were rather small and thus a successful balancing was not guaranteed, we abstained from using entropy balancing. Therefore, we directly adjusted for several sociodemographic characteristics (age, sex, race, highest degree of education, number of children in household, marital status, general health, and other behavioral risk factors) to warrant a consistent approach in answering the second question.

All analyses were performed using Stata 15.1 [StataCorp. 2017. Stata Statistical Software: Release 15. College Station, TX: StataCorp LLC]. The significance level was set to 0.05.

## Results

### Sample description

Table 1 summarizes sociodemographic, health, and caregiving-related characteristics for the groups of NCG, CG, and DCG. The study sample consisted of 45,925 NCG and 12,044 CG, of whom 1214 are DCG. The distribution of age and sex significantly differed between the comparison groups, with NCG being significantly younger than CG/DCG (mean age: 55.2, 56.1, and 59.7 years) and NCG including a significantly lower share of women (53.8% vs. 63% and 66.2%). In each group, the race category ‘white only’ dominated the sample, although, the distribution of race significantly differed between the groups. A large share of the study population is more highly educated, lives without children, reports good to excellent general health, and only few days in poor physical or mental health. However, CG and DCG report a significantly higher amount of days in poor mental health than NCG. In the unweighted sample, CG and DCG are less frequently binge drinkers or physically inactive but more frequently overweight/obese. CG are also more likely to be smokers. Most CG and DCG provide informal care for less than 9 h per week and for more than 2 years. Those caring for a parent (-in-law) or a grandparent are the largest group of CG/DCG, followed by spouses. Most of the CG/DCG do either help with household tasks only or provide both (household and personal care tasks).

**Table 1:** Sociodemographic and caregiving-related characteristics of US-American informal (dementia) caregivers and non-caregivers from the Behavioral Risk Factor Surveillance System 2017

Variable	Percentage^a^		
	Non-caregiver (*n* = 45,925)	Caregiver (*n* = 12,044)	Dementia caregiver (*n* = 1214)
Female*	53.8	63	66.2
Mean age* (SD)	55.2 (17.4)	56.1 (15.5)	59.7 (13.8)
Race*			
White only	70.6	71.4	73.3
Black only	5.1	5.4	4.6
Other race, non-hispanic	8.9	8	8.5
Multiracial, non-hispanic	4.4	5.1	5.4
Hispanic	9.3	8.2	6.3
Education*			
Graduated college/technical school	40.6	40.2	45
Attended college/technical school	27.1	31.1	30.6
Graduated high school	25.8	23.8	22
Did not graduate high school	6.12	4.7	2.3
Income*^2^			
≥ $50,000	44.7	44.4	50.2
< $50,000	41	42.2	38
Children in household*			
0	73.2	74.6	81.9
1–2	19.8	18.7	14.1
≥ 3	6.5	6.3	3.5
Living without partner*	45.1	38.4	35.3
General health status good to excellent*^1^	82.2	80.1	81.1
Days in poor mental health (per month)*			
0–5	83.9	77.2	78.7
6–15	7.7	10.2	9
> 15	7	11.2	10.8
Days in poor physical health (per month)*^1^			
0–5	78	77	77.8
6–15	8	9.9	9.7
> 15	10	11.6	10.5
Overweight/obese*	65.3	68.6	69.0
Binge drinker*	13.1	11.04	8.2
Smoker*^1^	12.6	16.2	12.6
Insufficiently physically activity*	47.4	43.2	43.7
Caregiving intensity			
< 9 h		60.2	53.2
9–39 h.		22.5	23.2
40+ h		17.3	23.6
Caregiving time			
< 30 days		18.3	8.1
1 month to < 2 years		31.4	35.4
> 2 years		50.4	56.5
Relationship to care recipient			
Spouse		19.8	18.5
Parent(-in-law)/grandparent		38.8	58.8
Child/Grandchild		10.9	1.7
Sibling(-in-law)		8.2	6.0
Other relative/friend		6.5	5.2
Non-relative/friend		15.8	9.8
Caregiving tasks			
Other tasks		16.9	12.4
Household tasks only		34.2	26.4
Personal care tasks only		5.3	6.5
Both		43.6	54.8

^a^Percentages do not sum up to 100% in each variable due to missing data

*p < 0.05 for the statistical significance of the bivariate relationships between caregiver status (caregiver vs. non-caregiver & dementia caregiver vs. non-caregiver) and respective variables

*^1^*p* < 0.05 for the statistical significance of the bivariate relationships between caregiver status (caregiver vs. non-caregiver) and respective variables

*^2^*p* < 0.05 for the statistical significance of the bivariate relationships between caregiver status (dementia caregiver vs. non-caregiver) and respective variables

### Identification of differences

Entropy balancing was successful for all specified balancing terms. The reweighted NCG group matches the CG/DCG group in all of the three moments of the selected covariates (see Online Resource 1 for details).

We initially compared NCG to CG. The weighted logistic regression analysis (Table 2) shows that, compared to NCG, the odds of being overweight or obese are 14% higher for CG (95% CI [1.09–1.19]). Considering the multinomial analysis, the relative risk ratios (RRR) of overweight (RRR = 1.09, 95% CI [1.03–1.15]) as well as of obesity (RRR = 1.17, 95% CI [1.12–1.23]) are significantly higher for CG. The odds of smoking are 1.34 times as great for CG compared to NCG (95% CI [1.26–1.42]) and 1.37 times as great for being a daily smoker (95% CI [1.28–1.47]). On the other hand, caregiving is associated with more responsible alcohol consumption (OR = 0.86, 95% CI [0.81–0.92]) and meeting the recommended weekly physical activity level (OR = 0.83, 95% CI [0.79–0.86]). The RRR of being a more than moderate or high-risk drinker are slightly lower for CG than for NCG, even though, the relationship between CG status and high-risk drinking is not statistically significant.

**Table 2:** Logistic regression models of entropy balanced caregivers and non-caregivers on selected risk factors, sample of US-American adults from the Behavioral Risk Factor Surveillance System 2017

Outcome	OR/RRR (95% CI) for caregivers vs. non-caregivers	OR/RRR (95% CI) for dementia caregivers vs. non-caregivers
**Overweight/obesity**	1.14*** (1.09–1.19)	1.19** (1.04–1.35)
Underweight	0.85 (0.71–1.01)	0.68 (0.39–1.19)
Normal weight	Ref. category	Ref. category
Overweight	1.09** (1.03–1.15)	1.09 (0.94–1.27)
Obese	1.17*** (1.12–1.23)	1.25** (1.08–1.45)
**Binge drinker**	0.86*** (0.81–0.92)	0.72** (0.58–0.89)
Mod./non-drinker	Ref. category	Ref. category
> Moderate	0.90*** (0.85–0.95)	0.89 (0.76–1.05)
High-risk drinker	0.95 (0. 86–1.05)	0.91 (0.67–1.24)
**Smoking**	1.34*** (1.26–1.42)	1.23* (1.04–1.46)
Non-smoker	Ref. category	Ref. category
Some days	1.26*** (1.14–1.39)	1.10 (0.80–1.51)
Every day	1.37*** (1.28–1.47)	1.30* (1.06–1.57)
**Insufficient physical activity**	0.83*** (0.79–0.86)	0.91 (0.81–1.03)
Highly active	1.04 (0.98–1.12)	0.96 (0.82–1.14)
Active	Ref. category	Ref. category
Insufficiently active	0.85*** (0.79–0.91)	0.94 (0.77–1.14)
Inactive	0.85*** (0.80–0.9)	0.86 (0.72–1.03)

Balanced for age, sex, race, highest educational degree, employment status, number of children, marital status, days in poor mental and physical health and for each risk behavior the distribution of the other risk factors was balanced, except for overweight and physical inactivity where the respective other risk factor was not included

*p < 0.05 **p < 0.01 ***p < 0.001

When considering different age groups (Table 3), the association between caregiving and being overweight/obese remains significant. The odds of being overweight/obese are highest for CG aged 65 and older (OR = 1.17, 95% CI [1.08–1.26]) compared to the same age group of NCG, but are only marginally higher than for people aged 18–49. For smoking, the odds decrease with increasing age group but remain significantly higher for CG compared to NCG. For binge drinking, the odds are lower for CG than for NCG when considering the age group 65 and older (OR = 0.73, 95% CI [0.62–0.88]) and 18–49 (OR = 0.86, 95% CI [0.78–0.95]), but not significant for people aged 50–64 (OR = 0.93, 95% CI [0.83–1.04]). Regarding the risk of insufficient physical activity, differences between CG and NCG in favor of CG seem to diminish with increasing age (18–49: OR = 0.73, 95% CI [0.67–0.78]; 50–64: OR = 0.86, 95% CI [0.80–0.92]; 65+: OR = 0.93, 95% CI [0.86–1.00]).

**Table 3:** Age-stratified logistic regression models of entropy balanced caregivers and non-caregivers on selected risk factors, sample of US-American adults from the Behavioral Risk Factor Surveillance System 2017

Outcome	Odds ratios (95% CI) for caregivers vs. non-caregivers		
	Age 18–49	Age 50–64	Age 65+
Overweight/obesity	1.16** (1.06–1.25)	1.09* (1.00–1.18)	1.17*** (1.08–1.26)
Binge drinker	0.86** (0.78–0.95)	0.93 (0.83–1.04)	0.73** (0.62–0.88)
Smoking	1.42*** (1.30–1.56)	1.28*** (1.17–1.41)	1.18* (1.04–1.35)
Insufficient physical activity	0.73*** (0.67–0.78)	0.86*** (0.80–0.92)	0.93 (0.86–1.00)

*p < 0.05 **p < 0.01 ***p < 0.001

Second, we compared NCG–DCG. The results of the binomial and multinomial logistic regression analyses point in a similar direction, however, some estimates revealed a lack of significance (Table 2). The odds of being overweight/obese or a current smoker are significantly higher for DCG than for NCG, but in the multinomial analysis, only the RRR for the more extreme manifestations ‘obesity’ (RRR = 1.25, 95% CI [1.08–1.45]) or ‘being a daily smoker’ (RRR = 1.3, 95% CI [1.06–1.57]) remained significant. The odds of binge drinking are lower for DCG than for NCG (OR = 0.72, 95% CI [0.58–0.89]), but non-significantly lower for more than moderate or high-risk drinking. Concerning insufficient physical activity, the tendency of DCG being less likely to not meet the recommendations can also be found. However, the differences are non-significant.

### Identification of associated factors

Table 4 displays the relationship between CG-related variables and selected risk factors for CG. Higher CG intensity increases the odds of being overweight/obese (9–39 h: OR = 1.11, 95% CI [0.98–1.24]; 40+ h: OR = 1.22, 95% CI [1.06–1.40]). Compared to spousal CG, the odds of being overweight/obese are significantly higher in those providing care for a parent/grandparent, child/grandchild, or another relative. Furthermore, greater odds of overweight/obesity can be seen for household tasks only, personal care only, and the combination of both, compared to other tasks.

**Table 4:** Logistic regression models of caregiving-related variables on selected risk factors in caregivers (odds ratios), sample of US-American adults from the Behavioral Risk Factor Surveillance System 2017

Variables	Overweight/obesity	Binge drinker	Smoking	Physical inactivity
	(95% CI)	(95% CI)	(95% CI)	(95% CI)
**Caregiving intensity**				
< 9 h	Ref. category	Ref. category	Ref. category	Ref. category
9–39 h	1.11	1.15	1.23**	0.88*
	(0.98–1.24)	(0.97–1.36)	(1.06–1.43)	(0.79–0.98)
40 h+	1.22**	0.95	1.37***	1.01
	(1.06–1.40)	(0.77–1.18)	(1.15–1.63)	(0.89–1.15)
**Caregiving time**				
< 30 days	Ref. category	Ref. category	Ref. category	Ref. category
1 month to < 2 years	0.88*	1.00	1.10	1.05
	(0.77–1.00)	(0.83–1.20)	(0.92–1.32)	(0.92–1.19)
> 2 years	0.98	0.78**	1.24*	1.06
	(0.86–1.11)	(0.65–0.94)	(1.04–1.48)	(0.94–1.20)
**Relationship to care recipient**				
Spouse/partner	Ref. category	Ref. category	Ref. category	Ref. category
Parent/parent-in-law/grandparent	1.29***	1.31*	0.85	0.74***
	(1.12–1.48)	(1.05–1.64)	(0.70–1.03)	(0.65–0.84)
Child/grandchild	1.22*	1.47**	0.77*	0.73***
	(1.02–1.46)	(1.10–1.95)	(0.61–0.98)	(0.61–0.86)
Sibling(-in-law)	1.16	1.20	0.85	0.60***
	(0.96–1.42)	(0.87–1.65)	(0.65–1.10)	(0.50–0.72)
Other relative	1.29*	1.51**	0.89	0.67***
	(1.03–1.60)	(1.11–2.07)	(0.67–1.18)	(0.55–0.82)
Non-relative/family friend	1.06	1.20	1.15	0.58***
	(0.89–1.25)	(0.92–1.58)	(0.92–1.44)	(0.49–0.68)
**Type of caregiving tasks**				
Other tasks	Ref. category	Ref. category	Ref. category	Ref. category
Household tasks only	1.19*	1.04	1.22*	0.86*
	(1.04–1.37)	(0.84–1.28)	(1.01–1.49)	(0.75–0.98)
Personal care only	1.48**	0.88	1.33	0.97
	(1.17–1.87)	(0.62–1.23)	(0.98–1.80)	(0.78–1.20)
Both	1.18*	0.89	1.28*	0.91
	(1.02–1.36)	(0.72–1.11)	(1.04–1.56)	(0.79–1.04)
**Observations**	9508	9508	9508	9508

*p < 0.05 **p < 0.01 ***p < 0.001

CG intensity and the type of CG tasks have no significant influence on being a binge drinker. Regarding CG time, significantly lower odds of binge drinking can be found for people caring for more than 2 years compared to those being a CG for less than 30 days (OR = 0.78, 95% CI [0.65–0.94]). Considering the relationship to the CG recipient, the likelihood of being a binge drinker is significantly higher for CG who care for a parent/grandparent, child/gra.ndchild, or another relative than for a spouse/partner.

Higher CG intensity and CG time of more than 2 years are associated with higher odds ratios of smoking, whereas giving care for a child/grandchild is associated with lower odds compared to spouses. Providing household tasks only or personal care and household tasks increase the odds of smoking by 22% (95% CI [1.01–1.49]) and 28% (95% CI [1.04–1.56]).

Physical inactivity is associated with providing 9–39 h of care per week (OR = 0.88, 95% CI [0.79–0.98] for 9–39 h vs. < 9 h) and being a spousal CG since odds ratios for all other relationships to the CG recipient are significantly lower.

The analyses of associated factors with behavioral risks for DCG (Table 5) reveal only a few significant results. Providing care for a parent(-in-law)/grandparent significantly increases the risk of being overweight/obese compared to spousal caregivers (OR = 1.83, 95% CI [1.13–2.97]).

**Table 5:** Logistic regression models of caregiving-related variables on selected risk factors in dementia caregivers (odds ratios), sample of US-American adults from the Behavioral Risk Factor Surveillance System 2017

Variables	Overweight/obesity (95% CI)	Binge drinker (95% CI)	Smoking (95% CI)	Physical inactivity (95% CI)
**Caregiving intensity**				
< 9 h	Ref. category	Ref. category	Ref. category	Ref. category
9–39 h	0.92	1.02	1.31	0.66*
	(0.64–1.33)	(0.56–1.86)	(0.75–2.28)	(0.46–0.94)
40+ h	1.19	0.60	1.29	0.88
	(0.77–1.82)	(0.27–1.33)	(0.71–2.32)	(0.60–1.31)
**Caregiving time**				
< 30 days	Ref. category	Ref. category	Ref. category	Ref. category
1 month to < 2 years	1.10	0.67	0.90	1.10
	(0.64–1.90)	(0.32–1.41)	(0.38–2.13)	(0.65–1.85)
> 2 years	0.95	0.42*	0.95	0.90
	(0.55–1.63)	(0.20–0.90)	(0.40–2.25)	(0.54–1.51)
**Relationship to care recipient**				
Spouse/partner	Ref. category	Ref. category	Ref. category	Ref. category
Parent(-in-law)/grandparent	1.83*	2.04	1.04	0.65
	(1.13–2.97)	(0.65–6.39)	(0.47–2.31)	(0.41–1.02)
Child/grandchild	0.78	empty	0.75	0.23*
	(0.29–2.13)		(0.16–3.57)	(0.075–0.72)
Sibling(-in-law)	0.84	2.77	0.65	0.32**
	(0.42–1.69)	(0.64–11.9)	(0.20–2.06)	(0.15–0.66)
Other relative	1.37	1.41	0.60	0.63
	(0.64–2.95)	(0.27–7.48)	(0.17–2.04)	(0.30–1.31)
Non-relative/friend	0.89	1.80	0.21**	0.30***
	(0.48–1.65)	(0.45–7.16)	(0.068–0.67)	(0.16–0.55)
**Type of caregiving task**				
Other tasks	Ref. category	Ref. category	Ref. category	Ref. category
Household tasks only	1.03	2.22	0.61	0.66
	(0.62–1.71)	(0.83–5.88)	(0.27–1.37)	(0.40–1.09)
Personal care only	1.76	1.56	0.46	0.89
	(0.83–3.72)	(0.43–5.70)	(0.13–1.67)	(0.45–1.75)
Both	1.26	1.87	0.88	1.04
	(0.77–2.07)	(0.71–4.89)	(0.41–1.88)	(0.65–1.68)
Observations	991	971	991	991

*p < 0.05 **p < 0.01 ***p < 0.001

CG time of more than 2 years decreases the odds of being a binge drinker (OR = 0.42, 95% CI [0.20–0.90]).

Similar to the CG group, the likelihood of being a smoker tends to be higher with CG intensity of more than 9 h. Different to the analysis of CG, it seems that helping with only household or personal tasks decreases the odds of being a smoker. Nevertheless, these results are tendencies as they are non-significant.

For physical inactivity, CG intensity of 9–39 h per week is associated with lower odds (OR = 0.66, 95% CI [0.46–0.94]) compared to the reference category (< 9 h). Moreover, providing care for a child/grandchild, sibling, or non-relative/friend has significantly lower odds of physical inactivity compared to spousal DCG.

## Discussion

To our knowledge, this is the first study that compares CG and DCG with NCG regarding behavioral risk factors by using the entropy balancing approach to control for confounding. Being a CG/DCG is positively associated with being a smoker and being overweight/obese, but negatively associated with physical inactivity and risky alcohol consumption. The magnitude of these associations varies by age. As DCG are especially challenged by their services (Pinquart and Sörensen 2003, 2007; Schoenmakers et al. 2010) it is interesting to see that they show behaviors comparable to CG. In case of binge drinking, the OR was even smaller. However, CG caring for people with different diseases or disorders are quite heterogeneous. For this reason, future studies should perform comparisons of CG for people with specific diseases. Due to small sample sizes, we were not able to perform these analyses in a reasonable manner.

Moreover, higher CG intensity is associated with higher odds of being overweight/obese and of being a smoker, but with lower odds of physical inactivity. With increasing CG time, the odds of being overweight/obese and being a binge drinker decrease, whereas the odds of being a smoker increase. Spousal CG have a lower likelihood of being overweight/obese or being a binge drinker, but have higher odds of being physically inactive than CG with other types of relationships to the CG recipient. Providing household tasks only or a combination of household and personal care tasks is associated with higher odds of being overweight/obese and being a smoker. Furthermore, the likelihood of physical inactivity is lower for those providing household tasks only.

In the analysis regarding the identification of associated factors for DCG, the odds are sometimes contrary to the results of CG. However, the confidence intervals are large and non-significant, which is a consequence of the small sample size. Thus, the results for the DCG should be treated as indicators for the drafting of hypotheses for future studies on DCG with larger sample sizes.

The results support previous findings. In an unadjusted analysis by Roth et al. (2013), CG were more frequently current smokers, but less frequently heavy drinkers. However, when CG were compared with propensity-matched NCG, the differences were no longer significant. Hoffman et al. (2012) found that CG from the baby boom generation had higher odds of smoking than NCG. Although their results are non-significant, the tendency that spousal CG are more likely to be current smokers could be found as well. In addition, our results support the findings of Reeves et al. (2012) who used the 2009 wave of the BRFSS to compare female CG and NCG regarding cancer risk behaviors and the utilization of breast and cervical cancer screening.

Even though our analysis is of cross-sectional nature and future research is needed to find causal evidence, we have possible hypotheses. Obesity is a risk factor that is not solely a result of behavior, as it can be explained by a variety of interacting factors like stress, inadequate sleep, overnutrition, inactivity or social pressure (Egger and Dixon 2014). Especially stress could play an important role in caregiving. On the one hand, CG may compensate stress with unhealthy diet (Razzoli et al. 2017; Tomiyama 2018) which we did not assess as single outcome in this study, because data was limited to fruit and vegetable consumption. Although some studies found a relationship between fruit and vegetable consumption and chronic diseases (Wang et al. 2014), the complexity of nutrition is better captured with multifactorial indices to predict health outcomes (Jacobs and Steffen 2003; Schwingshackl and Hoffmann 2015; Wirt and Collins 2009). On the other hand, stress has a direct impact on metabolic processes (Egger and Dixon 2014). Lower physical activity is less likely to explain the greater likelihood of obesity, because CG tend to be more active than NCG in this study. At this point, it is important to mention that it was possible in the questionnaire to list physical activities like gardening, yard work, and household tasks. Thus, the lower odds of being physically inactive may result from the fact that caregiving often includes these kind of activities. However, other factors may outweigh the beneficial influence of physical activity on obesity, since the effect of gardening/household tasks on BMI is maybe lower than, for example, a negative diet-related effect.

Whereas smoking is a typical habit to compensate stress for a short moment, which is easy to incorporate in a caregiving routine, high alcohol consumption probably entails more and especially longer lasting consequences like the inability to manage responsibilities associated with caregiving. DCG have even more stress (Pinquart and Sörensen 2003, 2007; Schoenmakers et al. 2010; van der Lee et al. 2014) and higher responsibility due to their care recipients’ extreme loss of cognitive function, which may explain the even higher likelihood of obesity and the even lower likelihood of being a binge drinker.

### Strengths and limitations

Compared to previous studies, the strengths of this study are the large sample size and the use of entropy balancing to achieve profound comparability between CG/DCG and NCG. However, this study has some limitations. First, the analyses are based on self-reported data which are prone to social desirability and recall biases. Second, only four behavioral risk factors were considered. However, these factors comprise three of the four main factors associated with non-communicable diseases (smoking, physical inactivity, harmful use of alcohol, unhealthy diets) (World Health Organization 2017). Third, caregiving-related variables were limited to the information gathered from the questionnaire and therefore may neglect important other factors. Fourth, the results are not generalizable to the U.S. population as we applied entropy balancing weights to adjust the structure of the covariates. This precluded the use of the BRFSS weighting scheme. Finally, the CG definition used in this study (providing regular care over the past 30 days for someone with a health problem or disability) may include some acute care situations. Thus, the results may not represent the population providing long-term assistance with activities of daily living to a dependent person. Moreover, the results may not be representative of older adults’ CG, since the definition also includes, for example, CG of persons with mental illnesses, addiction disorders, or developmental disabilities.

### Conclusions

In a comparison of NCG and CG/DCG from 12 US states, being a CG/DCG is associated with risky as well as health-promoting behavior. Since health behavior is influenced by psychosocial factors, it cannot be treated as independent factor to explain lower physical health in CG versus NCG. Future studies should study potential pathways between caregiving characteristics, psychological impacts of caregiving, health behavior, and mental or physical health.

## Electronic supplementary material

Below is the link to the electronic supplementary material.

Supplementary material 1 (PDF 2218 kb)
