# Supplementary material for: The association between informal caregiving and behavioral risk factors: a cross-sectional study
Source: Int J Public Health. 2020 Jun 9;65(6):911–21. doi: 10.1007/s00038-020-01402-6 (PMC7426287; doi:10.1007/s00038-020-01402-6)
Supplement: Supplementary file 1 — Supplementary material 1 (PDF 2218 kb) [file 38_2020_1402_MOESM1_ESM.pdf]

International Journal of Public Health.
The association between informal caregiving and behavioral risk factors: A cross-sectional study.

Electronic Supplementary Material 1 –

Covariate distribution before and after entropy balancing. US-American informal (dementia) caregivers and non-caregivers from the Behavioral Risk Factor Surveillance System 2017

Identification of differences – caregivers versus non-caregivers

Table A1 Covariate distribution before and after entropy balancing (caregivers versus non-caregivers) – Analysis of obesity in all age groups

Treated units: 11933 total of weights: 11933
Control units: 45416 total of weights: 11933

Before: without weighting

	Caregivers (treat)			Non-caregivers (control)		
	mean	variance	skewness	mean	variance	skewness
2.sex	.6295	.2332	-.5364	.539	.2485	-.1564
9.sex	.0005028	.0005026	44.56	.0004404	.0004402	47.62
_AGE80	56.15	241	-.5299	55.19	302.3	-.3922
2._RACEGR3	.05447	.05151	3.926	.05082	.04824	4.09
3._RACEGR3	.07969	.07335	3.104	.08865	.08079	2.894
4._RACEGR3	.0507	.04813	4.096	.04375	.04184	4.461
5._RACEGR3	.08238	.0756	3.038	.09261	.08404	2.811
9._RACEGR3	.01869	.01834	7.108	.01803	.01771	7.244
2._EDUCAG	.238	.1814	1.23	.2569	.1909	1.113
3._EDUCAG	.3107	.2142	.8179	.2715	.1978	1.028
4._EDUCAG	.4027	.2405	.3969	.4067	.2413	.3798
9._EDUCAG	.001927	.001924	22.71	.003281	.00327	17.37
2.EMPLOY1	.09553	.08641	2.752	.08944	.08144	2.877
3.EMPLOY1	.02866	.02784	5.65	.02129	.02084	6.632
4.EMPLOY1	.02154	.02107	6.592	.02072	.02029	6.729
5.EMPLOY1	.06176	.05795	3.641	.04853	.04618	4.202
6.EMPLOY1	.01961	.01923	6.929	.02728	.02654	5.804
7.EMPLOY1	.2904	.2061	.9236	.2989	.2095	.8788
8.EMPLOY1	.06989	.06501	3.374	.0637	.05964	3.573
9.EMPLOY1	.006117	.006081	12.67	.006914	.006866	11.9
2._INCOMG	.1362	.1176	2.122	.1316	.1143	2.18
3._INCOMG	.09185	.08342	2.826	.08327	.07634	3.017
4._INCOMG	.1226	.1076	2.301	.1179	.104	2.37
5._INCOMG	.4462	.2471	.2166	.4491	.2474	.2047
9._INCOMG	.1317	.1143	2.179	.1403	.1206	2.071
2._CHLDCNT	.102	.09159	2.63	.1067	.09528	2.549
3._CHLDCNT	.08506	.07783	2.975	.09197	.08351	2.824
4._CHLDCNT	.03654	.03521	4.94	.04029	.03867	4.675
5._CHLDCNT	.01726	.01697	7.412	.01603	.01577	7.707
6._CHLDCNT	.009721	.009627	9.994	.008852	.008773	10.49
9._CHLDCNT	.004022	.004007	15.67	.004492	.004472	14.82
2.marital	.1328	.1152	2.164	.137	.1182	2.111
3.marital	.08296	.07609	3.024	.1237	.1084	2.286
4.marital	.01936	.01898	6.977	.01955	.01917	6.94
5.marital	.1485	.1265	1.977	.1707	.1416	1.751
6.marital	.03377	.03263	5.162	.03195	.03093	5.323
9.marital	.006034	.005998	12.76	.005086	.005061	13.91
1.menthlth1	.1886	.1531	1.592	.1559	.1316	1.897
2.menthlth1	.06201	.05817	3.632	.04562	.04354	4.355
3.menthlth1	.04073	.03907	4.647	.03149	.0305	5.366
4.menthlth1	.02355	.023	6.284	.01451	.0143	8.12
5.menthlth1	.009302	.009216	10.22	.005791	.005758	13.03
6.menthlth1	.07886	.07264	3.125	.04928	.04685	4.165
77.menthlth1	.009972	.009874	9.863	.009996	.009897	9.851
99.menthlth1	.003771	.003757	16.19	.004338	.004319	15.08
1.physhlth1	.2129	.1676	1.403	.1799	.1476	1.666
2.physhlth1	.06235	.05847	3.62	.04915	.04673	4.171
3.physhlth1	.03645	.03513	4.947	.03142	.03043	5.372
4.physhlth1	.01877	.01842	7.092	.01339	.01321	8.468
5.physhlth1	.01115	.01102	9.313	.007905	.007842	11.11
6.physhlth1	.08573	.07839	2.959	.07878	.07258	3.127
77.physhlth1	.01215	.012	8.906	.01462	.01441	8.088
99.physhlth1	.003101	.003091	17.88	.004624	.004603	14.6

1.AlcCatb	.1461	.1248	2.003	.1483	.1263	1.979
2.AlcCatb	.04567	.04359	4.352	.04943	.04699	4.157
77.AlcCatb	.005196	.005169	13.76	.00665	.006606	12.14
99.AlcCatb	.001425	.001423	26.44	.001585	.001583	25.06
2._RFSMOK3	.1602	.1346	1.853	.1246	.1091	2.274
9._RFSMOK3	.005112	.005086	13.88	.005791	.005758	13.03

After: _uw1aObesity as the weighting variable

	Caregivers (treat)			Non-caregivers (control)		
	mean	variance	skewness	mean	variance	skewness
2.sex	.6295	.2332	-.5364	.6295	.2332	-.5363
9.sex	.0005028	.0005026	44.56	.0005028	.0005026	44.56
_AGE80	56.15	241	-.5299	56.15	241	-.5298
2._RACEGR3	.05447	.05151	3.926	.05447	.05151	3.926
3._RACEGR3	.07969	.07335	3.104	.0797	.07335	3.104
4._RACEGR3	.0507	.04813	4.096	.0507	.04813	4.096
5._RACEGR3	.08238	.0756	3.038	.08238	.0756	3.038
9._RACEGR3	.01869	.01834	7.108	.01869	.01834	7.108
2._EDUCAG	.238	.1814	1.23	.238	.1814	1.23
3._EDUCAG	.3107	.2142	.8179	.3108	.2142	.8178
4._EDUCAG	.4027	.2405	.3969	.4027	.2405	.3969
9._EDUCAG	.001927	.001924	22.71	.001928	.001924	22.71
2.EMPLOY1	.09553	.08641	2.752	.09554	.08641	2.752
3.EMPLOY1	.02866	.02784	5.65	.02866	.02784	5.65
4.EMPLOY1	.02154	.02107	6.592	.02154	.02107	6.592
5.EMPLOY1	.06176	.05795	3.641	.06176	.05795	3.641
6.EMPLOY1	.01961	.01923	6.929	.01961	.01923	6.929
7.EMPLOY1	.2904	.2061	.9236	.2904	.2061	.9235
8.EMPLOY1	.06989	.06501	3.374	.06989	.06501	3.374
9.EMPLOY1	.006117	.006081	12.67	.006118	.006081	12.67
2._INCOMG	.1362	.1176	2.122	.1362	.1176	2.121
3._INCOMG	.09185	.08342	2.826	.09185	.08341	2.826
4._INCOMG	.1226	.1076	2.301	.1226	.1076	2.301
5._INCOMG	.4462	.2471	.2166	.4462	.2471	.2166
9._INCOMG	.1317	.1143	2.179	.1317	.1143	2.179
2._CHLDCNT	.102	.09159	2.63	.102	.09159	2.63
3._CHLDCNT	.08506	.07783	2.975	.08506	.07783	2.975
4._CHLDCNT	.03654	.03521	4.94	.03654	.0352	4.94
5._CHLDCNT	.01726	.01697	7.412	.01726	.01697	7.412
6._CHLDCNT	.009721	.009627	9.994	.009721	.009627	9.994
9._CHLDCNT	.004022	.004007	15.67	.004023	.004007	15.67
2.marital	.1328	.1152	2.164	.1328	.1152	2.164
3.marital	.08296	.07609	3.024	.08297	.07609	3.024
4.marital	.01936	.01898	6.977	.01936	.01898	6.977
5.marital	.1485	.1265	1.977	.1485	.1265	1.977
6.marital	.03377	.03263	5.162	.03377	.03263	5.162
9.marital	.006034	.005998	12.76	.006034	.005998	12.76
1.menthlth1	.1886	.1531	1.592	.1886	.1531	1.592
2.menthlth1	.06201	.05817	3.632	.06201	.05817	3.632
3.menthlth1	.04073	.03907	4.647	.04073	.03907	4.647
4.menthlth1	.02355	.023	6.284	.02355	.02299	6.284
5.menthlth1	.009302	.009216	10.22	.009302	.009216	10.22
6.menthlth1	.07886	.07264	3.125	.07886	.07264	3.125
77.menthlth1	.009972	.009874	9.863	.009973	.009874	9.863
99.menthlth1	.003771	.003757	16.19	.003771	.003757	16.19
1.physhlth1	.2129	.1676	1.403	.2129	.1676	1.403
2.physhlth1	.06235	.05847	3.62	.06235	.05846	3.62
3.physhlth1	.03645	.03513	4.947	.03645	.03513	4.947
4.physhlth1	.01877	.01842	7.092	.01877	.01842	7.092
5.physhlth1	.01115	.01102	9.313	.01115	.01102	9.313
6.physhlth1	.08573	.07839	2.959	.08573	.07838	2.959
77.physhlth1	.01215	.012	8.906	.01215	.012	8.905
99.physhlth1	.003101	.003091	17.88	.003101	.003091	17.87
1.AlcCatb	.1461	.1248	2.003	.1462	.1248	2.003
2.AlcCatb	.04567	.04359	4.352	.04567	.04359	4.352
77.AlcCatb	.005196	.005169	13.76	.005196	.005169	13.76
99.AlcCatb	.001425	.001423	26.44	.001425	.001423	26.44
2._RFSMOK3	.1602	.1346	1.853	.1602	.1346	1.852
9._RFSMOK3	.005112	.005086	13.88	.005112	.005086	13.88

Table A2 Covariate distribution before and after entropy balancing (caregivers versus non-caregivers) – Analysis of obesity, age group 18-49

Treated units: 3494 total of weights: 3494
Control units: 15845 total of weights: 3494

Before: without weighting

	Caregivers (treat)			Non-caregivers (control)		
	mean	variance	skewness	mean	variance	skewness
_AGE80	36.25	80.75	-.3605	34.94	81.16	-.167
2.sex	.6116	.2376	-.458	.4979	.25	.008457
9.sex	.0008586	.0008581	34.08	.0004418	.0004416	47.55
2._RACEGR3	.07012	.06522	3.367	.05882	.05536	3.75
3._RACEGR3	.1073	.09584	2.537	.1125	.09987	2.452
4._RACEGR3	.0644	.06027	3.549	.06349	.05946	3.58
5._RACEGR3	.1448	.1239	2.019	.1508	.128	1.952
9._RACEGR3	.02089	.02046	6.7	.01452	.01431	8.118
2._EDUCAG	.2682	.1963	1.047	.2524	.1887	1.14
3._EDUCAG	.3251	.2195	.7466	.2785	.201	.9882
4._EDUCAG	.3483	.2271	.6368	.4038	.2408	.3919
9._EDUCAG	.001717	.001715	24.07	.003282	.003271	17.37
2.EMPLOY1	.09302	.08439	2.802	.08829	.0805	2.902
3.EMPLOY1	.03749	.0361	4.869	.02348	.02293	6.294
4.EMPLOY1	.04264	.04084	4.527	.03515	.03392	5.048
5.EMPLOY1	.08901	.08111	2.887	.06204	.05819	3.631
6.EMPLOY1	.06268	.05877	3.608	.07554	.06984	3.212
7.EMPLOY1	.004865	.004843	14.23	.004986	.004961	14.06
8.EMPLOY1	.06068	.05701	3.68	.0414	.03969	4.604
9.EMPLOY1	.008014	.007952	11.04	.009782	.009687	9.962
2._INCOMG	.16	.1344	1.855	.1278	.1115	2.23
3._INCOMG	.09273	.08416	2.808	.07681	.07091	3.179
4._INCOMG	.1151	.1018	2.413	.1128	.1001	2.448
5._INCOMG	.4184	.2434	.3307	.4896	.2499	.04154
9._INCOMG	.1173	.1036	2.378	.1215	.1067	2.317
2._CHLDCNT	.1866	.1518	1.609	.1986	.1592	1.511
3._CHLDCNT	.2175	.1703	1.369	.2098	.1658	1.425
4._CHLDCNT	.1002	.09016	2.663	.09738	.0879	2.716
5._CHLDCNT	.05066	.04811	4.098	.03957	.03801	4.724
6._CHLDCNT	.03005	.02916	5.505	.02146	.021	6.605
9._CHLDCNT	.006869	.006824	11.94	.008709	.008634	10.57
2.marital	.0996	.0897	2.674	.08514	.07789	2.973
3.marital	.006869	.006824	11.94	.007132	.007081	11.71
4.marital	.02719	.02646	5.814	.02493	.02431	6.094
5.marital	.3151	.2159	.796	.3251	.2194	.7468
6.marital	.06497	.06077	3.53	.06273	.0588	3.607
9.marital	.006869	.006824	11.94	.004923	.004899	14.15
1.menthlth1	.2272	.1757	1.302	.2112	.1666	1.415
2.menthlth1	.08958	.08158	2.874	.06418	.06007	3.557
3.menthlth1	.06211	.05827	3.629	.04443	.04246	4.422
4.menthlth1	.03463	.03344	5.09	.01919	.01882	7.01
5.menthlth1	.01231	.01216	8.847	.008331	.008262	10.82
6.menthlth1	.1093	.09741	2.504	.05484	.05184	3.91
77.menthlth1	.009731	.009639	9.989	.007826	.007765	11.17
99.menthlth1	.00229	.002285	20.83	.002524	.002518	19.83
1.physhlth1	.247	.186	1.173	.2124	.1673	1.406
2.physhlth1	.06783	.06325	3.437	.04519	.04315	4.379
3.physhlth1	.03921	.03768	4.748	.02354	.02299	6.285
4.physhlth1	.01832	.01799	7.184	.009467	.009378	10.13
5.physhlth1	.01259	.01244	8.742	.005617	.005586	13.23
6.physhlth1	.06869	.06399	3.411	.04146	.03975	4.6
77.physhlth1	.0103	.0102	9.699	.008709	.008634	10.57
99.physhlth1	.002003	.002	22.27	.002777	.002769	18.9
1.AlcCatb	.1895	.1536	1.585	.2008	.1605	1.494
2.AlcCatb	.08586	.07851	2.956	.08886	.08097	2.89
77.AlcCatb	.009445	.009358	10.14	.008583	.00851	10.65
99.AlcCatb	.001145	.001144	29.5	.001199	.001198	28.83
2._RFSMOK3	.2327	.1786	1.265	.1529	.1295	1.929
9._RFSMOK3	.005438	.00541	13.45	.004481	.004461	14.84

After: _uw1aObesityB1 as the weighting variable

	Caregivers (treat)			Non-caregivers (control)		
	mean	variance	skewness	mean	variance	skewness
_AGE80	36.25	80.75	-.3605	36.25	80.75	-.3601
2.sex	.6116	.2376	-.458	.6116	.2376	-.4578
9.sex	.0008586	.0008581	34.08	.0008587	.000858	34.08
2._RACEGR3	.07012	.06522	3.367	.07013	.06522	3.367
3._RACEGR3	.1073	.09584	2.537	.1074	.09583	2.537
4._RACEGR3	.0644	.06027	3.549	.0644	.06026	3.549
5._RACEGR3	.1448	.1239	2.019	.1449	.1239	2.018
9._RACEGR3	.02089	.02046	6.7	.0209	.02046	6.699
2._EDUCAG	.2682	.1963	1.047	.2682	.1963	1.046
3._EDUCAG	.3251	.2195	.7466	.3252	.2195	.7463
4._EDUCAG	.3483	.2271	.6368	.3484	.227	.6365
9._EDUCAG	.001717	.001715	24.07	.001718	.001715	24.06
2.EMPLOY1	.09302	.08439	2.802	.09303	.08438	2.802
3.EMPLOY1	.03749	.0361	4.869	.0375	.03609	4.869
4.EMPLOY1	.04264	.04084	4.527	.04265	.04083	4.527
5.EMPLOY1	.08901	.08111	2.887	.08902	.0811	2.886
6.EMPLOY1	.06268	.05877	3.608	.06269	.05876	3.608
7.EMPLOY1	.004865	.004843	14.23	.004866	.004843	14.23
8.EMPLOY1	.06068	.05701	3.68	.06068	.057	3.68
9.EMPLOY1	.008014	.007952	11.04	.008015	.007951	11.04
2._INCOMG	.16	.1344	1.855	.16	.1344	1.855
3._INCOMG	.09273	.08416	2.808	.09274	.08415	2.808
4._INCOMG	.1151	.1018	2.413	.1151	.1018	2.412
5._INCOMG	.4184	.2434	.3307	.4185	.2434	.3305
9._INCOMG	.1173	.1036	2.378	.1174	.1036	2.377
2._CHLDCNT	.1866	.1518	1.609	.1867	.1518	1.608
3._CHLDCNT	.2175	.1703	1.369	.2176	.1702	1.369
4._CHLDCNT	.1002	.09016	2.663	.1002	.09016	2.663
5._CHLDCNT	.05066	.04811	4.098	.05066	.0481	4.098
6._CHLDCNT	.03005	.02916	5.505	.03006	.02915	5.505
9._CHLDCNT	.006869	.006824	11.94	.00687	.006823	11.94
2.marital	.0996	.0897	2.674	.09961	.08969	2.674
3.marital	.006869	.006824	11.94	.00687	.006823	11.94
4.marital	.02719	.02646	5.814	.02719	.02646	5.814
5.marital	.3151	.2159	.796	.3152	.2159	.7957
6.marital	.06497	.06077	3.53	.06498	.06076	3.53
9.marital	.006869	.006824	11.94	.00687	.006823	11.94
1.menthlth1	.2272	.1757	1.302	.2273	.1756	1.301
2.menthlth1	.08958	.08158	2.874	.08959	.08157	2.874
3.menthlth1	.06211	.05827	3.629	.06211	.05826	3.628
4.menthlth1	.03463	.03344	5.09	.03464	.03344	5.09
5.menthlth1	.01231	.01216	8.847	.01231	.01216	8.846
6.menthlth1	.1093	.09741	2.504	.1094	.0974	2.503
77.menthlth1	.009731	.009639	9.989	.009732	.009638	9.988
99.menthlth1	.00229	.002285	20.83	.00229	.002285	20.82
1.physhlth1	.247	.186	1.173	.2471	.186	1.173
2.physhlth1	.06783	.06325	3.437	.06784	.06324	3.437
3.physhlth1	.03921	.03768	4.748	.03922	.03768	4.748
4.physhlth1	.01832	.01799	7.184	.01832	.01799	7.184
5.physhlth1	.01259	.01244	8.742	.01259	.01244	8.741
6.physhlth1	.06869	.06399	3.411	.0687	.06398	3.41
77.physhlth1	.0103	.0102	9.699	.01031	.0102	9.697
99.physhlth1	.002003	.002	22.27	.002004	.002	22.27
1.AlcCatb	.1895	.1536	1.585	.1895	.1536	1.584
2.AlcCatb	.08586	.07851	2.956	.08587	.0785	2.956
77.AlcCatb	.009445	.009358	10.14	.009446	.009358	10.14
99.AlcCatb	.001145	.001144	29.5	.001145	.001144	29.5
2._RFSMOK3	.2327	.1786	1.265	.2327	.1786	1.265
9._RFSMOK3	.005438	.00541	13.45	.005439	.005409	13.45

Table A3 Covariate distribution before and after entropy balancing (caregivers versus non-caregivers) – Analysis of obesity, age group 50-64

Treated units: 4459 total of weights: 4459
Control units: 13325 total of weights: 4459

Before: without weighting

	Caregivers (treat)			Non-caregivers (control)		
	mean	variance	skewness	mean	variance	skewness
_AGE80	57.47	17.87	-.1234	57.53	18.47	-.1554
2.sex	.6378	.2311	-.5735	.5299	.2491	-.1198
9.sex	.0004485	.0004484	47.19	.0003752	.0003751	51.59
2._RACEGR3	.05629	.05313	3.85	.05403	.05112	3.945
3._RACEGR3	.07625	.07045	3.193	.08293	.07606	3.025
4._RACEGR3	.05225	.04953	4.024	.03805	.0366	4.829
5._RACEGR3	.06885	.06412	3.406	.07947	.07316	3.109
9._RACEGR3	.01727	.01697	7.411	.02004	.01964	6.85
2._EDUCAG	.2312	.1778	1.275	.253	.189	1.136
3._EDUCAG	.3124	.2149	.8095	.2693	.1968	1.04
4._EDUCAG	.4149	.2428	.3455	.4155	.2429	.3431
9._EDUCAG	.002243	.002238	21.05	.003152	.003142	17.73
2.EMPLOY1	.1191	.1049	2.352	.1232	.1081	2.293
3.EMPLOY1	.03476	.03356	5.08	.03152	.03053	5.363
4.EMPLOY1	.02108	.02064	6.668	.02296	.02244	6.369
5.EMPLOY1	.05001	.04752	4.129	.03602	.03473	4.98
6.EMPLOY1	.002243	.002238	21.05	.002402	.002396	20.33
7.EMPLOY1	.1377	.1188	2.103	.137	.1183	2.111
8.EMPLOY1	.115	.1018	2.413	.1198	.1054	2.342
9.EMPLOY1	.005607	.005576	13.24	.007129	.007079	11.72
2._INCOMG	.1133	.1005	2.441	.1123	.09973	2.455
3._INCOMG	.0758	.07007	3.205	.06402	.05992	3.562
4._INCOMG	.1076	.09608	2.532	.09929	.08944	2.68
5._INCOMG	.5127	.2499	-.0507	.5153	.2498	-.06142
9._INCOMG	.1146	.1015	2.42	.1192	.105	2.351
2._CHLDCNT	.09935	.0895	2.679	.09936	.0895	2.679
3._CHLDCNT	.04642	.04428	4.312	.05156	.0489	4.056
4._CHLDCNT	.0157	.01546	7.792	.01644	.01617	7.607
5._CHLDCNT	.004934	.004911	14.13	.005478	.005449	13.4
6._CHLDCNT	.002243	.002238	21.05	.004053	.004036	15.61
9._CHLDCNT	.003813	.003799	16.1	.003977	.003962	15.76
2.marital	.1624	.136	1.831	.181	.1483	1.657
3.marital	.05203	.04933	4.034	.05666	.05345	3.835
4.marital	.02063	.02021	6.745	.02364	.02308	6.271
5.marital	.1081	.09643	2.524	.1167	.1031	2.388
6.marital	.02826	.02747	5.694	.02274	.02222	6.403
9.marital	.007176	.007127	11.68	.006079	.006042	12.71
1.menthlth1	.1902	.154	1.579	.1467	.1252	1.997
2.menthlth1	.05584	.05274	3.869	.04255	.04074	4.533
3.menthlth1	.04037	.03875	4.671	.03047	.02954	5.464
4.menthlth1	.02243	.02193	6.451	.01629	.01602	7.643
5.menthlth1	.009868	.009773	9.917	.005854	.00582	12.96
6.menthlth1	.08118	.07461	3.067	.05854	.05511	3.761
77.menthlth1	.008298	.008231	10.84	.01028	.01018	9.709
99.menthlth1	.00471	.004688	14.47	.005478	.005449	13.4
1.physhlth1	.2095	.1656	1.428	.1747	.1442	1.713
2.physhlth1	.06033	.0567	3.693	.04968	.04722	4.145
3.physhlth1	.03678	.03543	4.922	.03452	.03333	5.099
4.physhlth1	.02288	.02236	6.383	.01606	.0158	7.7
5.physhlth1	.01099	.01087	9.381	.009681	.009588	10.02
6.physhlth1	.09778	.08824	2.708	.09966	.08974	2.673
77.physhlth1	.009643	.009553	10.04	.01126	.01113	9.265
99.physhlth1	.002915	.002908	18.44	.004203	.004185	15.33
1.AlcCatb	.1651	.1378	1.804	.1537	.1301	1.92
2.AlcCatb	.04037	.03875	4.671	.04233	.04054	4.546
77.AlcCatb	.003813	.003799	16.1	.005854	.00582	12.96
99.AlcCatb	.00157	.001568	25.18	.001726	.001723	24.01
2._RFSMOK3	.1743	.1439	1.717	.1484	.1264	1.978
9._RFSMOK3	.004037	.004021	15.64	.004803	.00478	14.33

After: _uw1aObesityB2 as the weighting variable

	Caregivers (treat)			Non-caregivers (control)		
	mean	variance	skewness	mean	variance	skewness

_AGE80	57.47	17.87	-.1234	57.47	17.87	-.1223
2.sex	.6378	.2311	-.5735	.6378	.231	-.5733
9.sex	.0004485	.0004484	47.19	.0004486	.0004484	47.18
2._RACEGR3	.05629	.05313	3.85	.0563	.05313	3.85
3._RACEGR3	.07625	.07045	3.193	.07626	.07045	3.193
4._RACEGR3	.05225	.04953	4.024	.05226	.04953	4.024
5._RACEGR3	.06885	.06412	3.406	.06886	.06412	3.405
9._RACEGR3	.01727	.01697	7.411	.01727	.01697	7.411
2._EDUCAG	.2312	.1778	1.275	.2313	.1778	1.275
3._EDUCAG	.3124	.2149	.8095	.3124	.2148	.8093
4._EDUCAG	.4149	.2428	.3455	.4149	.2428	.3454
9._EDUCAG	.002243	.002238	21.05	.002243	.002238	21.04
2.EMPLOY1	.1191	.1049	2.352	.1191	.1049	2.352
3.EMPLOY1	.03476	.03356	5.08	.03476	.03356	5.079
4.EMPLOY1	.02108	.02064	6.668	.02108	.02064	6.667
5.EMPLOY1	.05001	.04752	4.129	.05002	.04752	4.129
6.EMPLOY1	.002243	.002238	21.05	.002243	.002238	21.04
7.EMPLOY1	.1377	.1188	2.103	.1377	.1188	2.103
8.EMPLOY1	.115	.1018	2.413	.1151	.1018	2.413
9.EMPLOY1	.005607	.005576	13.24	.005607	.005576	13.24
2._INCOMG	.1133	.1005	2.441	.1133	.1005	2.44
3._INCOMG	.0758	.07007	3.205	.07581	.07007	3.205
4._INCOMG	.1076	.09608	2.532	.1077	.09608	2.532
5._INCOMG	.5127	.2499	-.0507	.5127	.2499	-.05068
9._INCOMG	.1146	.1015	2.42	.1146	.1015	2.419
2._CHLDCNT	.09935	.0895	2.679	.09936	.08949	2.679
3._CHLDCNT	.04642	.04428	4.312	.04643	.04427	4.311
4._CHLDCNT	.0157	.01546	7.792	.0157	.01545	7.792
5._CHLDCNT	.004934	.004911	14.13	.004934	.00491	14.13
6._CHLDCNT	.002243	.002238	21.05	.002243	.002238	21.04
9._CHLDCNT	.003813	.003799	16.1	.003813	.003799	16.1
2.marital	.1624	.136	1.831	.1624	.136	1.831
3.marital	.05203	.04933	4.034	.05203	.04933	4.034
4.marital	.02063	.02021	6.745	.02063	.02021	6.744
5.marital	.1081	.09643	2.524	.1081	.09643	2.524
6.marital	.02826	.02747	5.694	.02826	.02746	5.693
9.marital	.007176	.007127	11.68	.007177	.007126	11.68
1.menthlth1	.1902	.154	1.579	.1902	.154	1.579
2.menthlth1	.05584	.05274	3.869	.05585	.05273	3.868
3.menthlth1	.04037	.03875	4.671	.04037	.03874	4.67
4.menthlth1	.02243	.02193	6.451	.02243	.02193	6.45
5.menthlth1	.009868	.009773	9.917	.009869	.009772	9.917
6.menthlth1	.08118	.07461	3.067	.08119	.0746	3.067
77.menthlth1	.008298	.008231	10.84	.008299	.00823	10.84
99.menthlth1	.00471	.004688	14.47	.00471	.004688	14.47
1.physhlth1	.2095	.1656	1.428	.2095	.1656	1.428
2.physhlth1	.06033	.0567	3.693	.06033	.0567	3.693
3.physhlth1	.03678	.03543	4.922	.03678	.03543	4.922
4.physhlth1	.02288	.02236	6.383	.02288	.02236	6.382
5.physhlth1	.01099	.01087	9.381	.01099	.01087	9.381
6.physhlth1	.09778	.08824	2.708	.09779	.08823	2.708
77.physhlth1	.009643	.009553	10.04	.009644	.009552	10.03
99.physhlth1	.002915	.002908	18.44	.002916	.002907	18.44
1.AlcCatb	.1651	.1378	1.804	.1651	.1378	1.804
2.AlcCatb	.04037	.03875	4.671	.04037	.03874	4.67
77.AlcCatb	.003813	.003799	16.1	.003813	.003799	16.1
99.AlcCatb	.00157	.001568	25.18	.00157	.001568	25.18
2._RFSMOK3	.1743	.1439	1.717	.1743	.1439	1.717
9._RFSMOK3	.004037	.004021	15.64	.004037	.004021	15.64

Table A4 Covariate distribution before and after entropy balancing (caregivers versus non-caregivers) – Analysis of obesity, age group 65+

Treated units: 3980 total of weights: 3980
Control units: 16246 total of weights: 3980

Before: without weighting

	Caregivers (treat)			Non-caregivers (control)		
	mean	variance	skewness	mean	variance	skewness

_AGE80	72.15	25.86	.2612	73.04	27.68	.0221
2.sex	.6359	.2316	-.565	.5865	.2425	-.3512
9.sex	.0002513	.0002513	63.06	.0004924	.0004922	45.03
2._RACEGR3	.03869	.03721	4.784	.04038	.03875	4.67
3._RACEGR3	.0593	.05579	3.732	.07005	.06515	3.369
4._RACEGR3	.03693	.03558	4.911	.02918	.02833	5.595
5._RACEGR3	.04271	.0409	4.523	.04666	.04448	4.299
9._RACEGR3	.01834	.01801	7.179	.01982	.01943	6.89
2._EDUCAG	.2191	.1711	1.358	.2646	.1946	1.068
3._EDUCAG	.2962	.2085	.8926	.2665	.1955	1.056
4._EDUCAG	.4367	.2461	.2553	.4023	.2405	.3984
9._EDUCAG	.001759	.001756	23.78	.003385	.003374	17.1
2.EMPLOY1	.07136	.06628	3.33	.06285	.0589	3.603
3.EMPLOY1	.01407	.01388	8.251	.01077	.01066	9.479
4.EMPLOY1	.003518	.003506	16.77	.004801	.004778	14.33
5.EMPLOY1	.05101	.04842	4.082	.04561	.04353	4.356
6.EMPLOY1	.001256	.001255	28.16	.0006155	.0006152	40.27
7.EMPLOY1	.7121	.2051	-.9367	.7182	.2024	-.9701
8.EMPLOY1	.02739	.02664	5.792	.03946	.0379	4.731
9.EMPLOY1	.005025	.005001	14	.003939	.003924	15.84
2._INCOMG	.141	.1211	2.064	.1511	.1282	1.949
3._INCOMG	.109	.09718	2.509	.1054	.09428	2.57
4._INCOMG	.146	.1247	2.005	.1381	.119	2.098
5._INCOMG	.396	.2392	.4254	.3552	.2291	.605
9._INCOMG	.1633	.1367	1.822	.176	.145	1.702
2._CHLDCNT	.03065	.02972	5.446	.02296	.02243	6.37
3._CHLDCNT	.01206	.01192	8.94	.01016	.01005	9.771
4._CHLDCNT	.00402	.004005	15.68	.004186	.004168	15.36
5._CHLDCNT	.001759	.001756	23.78	.001724	.001721	24.03
6._CHLDCNT	.0002513	.0002513	63.06	.0004924	.0004922	45.03
9._CHLDCNT	.001759	.001756	23.78	.0008002	.0007996	35.31
2.marital	.1289	.1123	2.215	.1515	.1286	1.944
3.marital	.1844	.1504	1.627	.2924	.2069	.9129
4.marital	.01106	.01094	9.352	.01096	.01084	9.396
5.marital	.04749	.04524	4.255	.06439	.06024	3.55
6.marital	.01256	.01241	8.753	.009479	.00939	10.12
9.marital	.00402	.004005	15.68	.004432	.004412	14.92
1.menthlth1	.153	.1296	1.928	.1095	.09752	2.501
2.menthlth1	.04472	.04273	4.405	.03004	.02914	5.507
3.menthlth1	.02236	.02187	6.461	.0197	.01931	6.913
4.menthlth1	.01508	.01485	7.959	.008494	.008423	10.71
5.menthlth1	.00603	.005995	12.76	.003262	.003252	17.42
6.menthlth1	.0495	.04706	4.154	.03626	.03494	4.962
77.menthlth1	.01206	.01192	8.94	.01188	.01174	9.01
99.menthlth1	.00402	.004005	15.68	.005171	.005144	13.8
1.physhlth1	.1867	.1519	1.608	.1525	.1293	1.933
2.physhlth1	.0598	.05624	3.713	.05257	.04981	4.01
3.physhlth1	.03367	.03254	5.171	.03656	.03523	4.938
4.physhlth1	.01457	.01436	8.102	.01502	.01479	7.975
5.physhlth1	.01005	.009952	9.824	.008679	.008604	10.59
6.physhlth1	.08719	.0796	2.927	.09805	.08845	2.703
77.physhlth1	.01658	.01631	7.571	.02314	.02261	6.343
99.physhlth1	.004271	.004254	15.2	.006771	.006725	12.03
1.AlcCatb	.08693	.0794	2.932	.09276	.08416	2.808
2.AlcCatb	.01633	.01607	7.632	.0168	.01652	7.518
77.AlcCatb	.003015	.003007	18.13	.005417	.005388	13.48
99.AlcCatb	.001508	.001506	25.7	.001847	.001843	23.21
2._RFSMOK3	.0809	.07438	3.074	.07737	.07139	3.164
9._RFSMOK3	.00603	.005995	12.76	.007879	.007817	11.13

After: _uw1aObesityB3 as the weighting variable

	Caregivers (treat)			Non-caregivers (control)		
	mean	variance	skewness	mean	variance	skewness
_AGE80	72.15	25.86	.2612	72.15	25.86	.2629
2.sex	.6359	.2316	-.565	.6359	.2316	-.5648
9.sex	.0002513	.0002513	63.06	.0002514	.0002514	63.05
2._RACEGR3	.03869	.03721	4.784	.0387	.0372	4.783
3._RACEGR3	.0593	.05579	3.732	.0593	.05579	3.732
4._RACEGR3	.03693	.03558	4.911	.03694	.03558	4.91

5._RACEGR3	.04271	.0409	4.523	.04272	.0409	4.523
9._RACEGR3	.01834	.01801	7.179	.01834	.01801	7.179
2._EDUCAG	.2191	.1711	1.358	.2191	.1711	1.358
3._EDUCAG	.2962	.2085	.8926	.2963	.2085	.8923
4._EDUCAG	.4367	.2461	.2553	.4367	.246	.2552
9._EDUCAG	.001759	.001756	23.78	.00176	.001757	23.78
2.EMPLOY1	.07136	.06628	3.33	.07136	.06627	3.33
3.EMPLOY1	.01407	.01388	8.251	.01407	.01387	8.251
4.EMPLOY1	.003518	.003506	16.77	.003518	.003506	16.77
5.EMPLOY1	.05101	.04842	4.082	.05101	.04841	4.081
6.EMPLOY1	.001256	.001255	28.16	.001256	.001255	28.16
7.EMPLOY1	.7121	.2051	-.9367	.712	.2051	-.9364
8.EMPLOY1	.02739	.02664	5.792	.02739	.02664	5.791
9.EMPLOY1	.005025	.005001	14	.005026	.005001	14
2._INCOMG	.141	.1211	2.064	.141	.1211	2.063
3._INCOMG	.109	.09718	2.509	.1091	.09718	2.508
4._INCOMG	.146	.1247	2.005	.146	.1247	2.005
5._INCOMG	.396	.2392	.4254	.396	.2392	.4253
9._INCOMG	.1633	.1367	1.822	.1634	.1367	1.821
2._CHLDCNT	.03065	.02972	5.446	.03066	.02972	5.445
3._CHLDCNT	.01206	.01192	8.94	.01206	.01192	8.94
4._CHLDCNT	.00402	.004005	15.68	.004021	.004005	15.68
5._CHLDCNT	.001759	.001756	23.78	.001759	.001756	23.78
6._CHLDCNT	.0002513	.0002513	63.06	.0002514	.0002513	63.05
9._CHLDCNT	.001759	.001756	23.78	.001759	.001756	23.78
2.marital	.1289	.1123	2.215	.1289	.1123	2.215
3.marital	.1844	.1504	1.627	.1845	.1505	1.627
4.marital	.01106	.01094	9.352	.01106	.01093	9.352
5.marital	.04749	.04524	4.255	.04749	.04524	4.255
6.marital	.01256	.01241	8.753	.01256	.01241	8.752
9.marital	.00402	.004005	15.68	.004021	.004005	15.68
1.menthlth1	.153	.1296	1.928	.153	.1296	1.927
2.menthlth1	.04472	.04273	4.405	.04473	.04273	4.405
3.menthlth1	.02236	.02187	6.461	.02236	.02187	6.46
4.menthlth1	.01508	.01485	7.959	.01508	.01485	7.959
5.menthlth1	.00603	.005995	12.76	.006031	.005995	12.76
6.menthlth1	.0495	.04706	4.154	.0495	.04706	4.154
77.menthlth1	.01206	.01192	8.94	.01206	.01192	8.94
99.menthlth1	.00402	.004005	15.68	.004021	.004005	15.68
1.physhlth1	.1867	.1519	1.608	.1867	.1519	1.608
2.physhlth1	.0598	.05624	3.713	.0598	.05623	3.713
3.physhlth1	.03367	.03254	5.171	.03367	.03254	5.17
4.physhlth1	.01457	.01436	8.102	.01457	.01436	8.101
5.physhlth1	.01005	.009952	9.824	.01005	.009952	9.823
6.physhlth1	.08719	.0796	2.927	.0872	.0796	2.926
77.physhlth1	.01658	.01631	7.571	.01659	.01631	7.57
99.physhlth1	.004271	.004254	15.2	.004272	.004254	15.2
1.AlcCatb	.08693	.0794	2.932	.08694	.07939	2.932
2.AlcCatb	.01633	.01607	7.632	.01633	.01607	7.632
77.AlcCatb	.003015	.003007	18.13	.003016	.003007	18.13
99.AlcCatb	.001508	.001506	25.7	.001508	.001506	25.7
2._RFSMOK3	.0809	.07438	3.074	.08091	.07437	3.074
9._RFSMOK3	.00603	.005995	12.76	.006031	.005995	12.76

Table A5 Covariate distribution before and after entropy balancing (caregivers versus non-caregivers) – Analysis of alcohol consumption, all age groups

Treated units: 12044 total of weights: 12044
Control units: 45929 total of weights: 12044

Before: without weighting

	Caregivers (treat)			Non-caregivers (control)		
	mean	variance	skewness	mean	variance	skewness
2.sex	.6295	.2332	-.5364	.5381	.2486	-.1528
9.sex	.0004982	.000498	44.77	.0005661	.0005658	41.99
_AGE80	56.14	241.1	-.5296	55.19	302.3	-.3926
2._RACEGR3	.0543	.05136	3.934	.0509	.04831	4.086
3._RACEGR3	.07962	.07329	3.106	.08855	.08071	2.897

4._RACEGR3	.05073	.04816	4.095	.04363	.04173	4.468
5._RACEGR3	.08212	.07538	3.044	.09293	.08429	2.804
9._RACEGR3	.01926	.01889	6.995	.01838	.01804	7.172
2._EDUCAG	.2384	.1816	1.228	.2578	.1913	1.108
3._EDUCAG	.3108	.2142	.8177	.2709	.1975	1.031
4._EDUCAG	.4021	.2404	.3993	.406	.2412	.3828
9._EDUCAG	.001993	.001989	22.33	.003527	.003515	16.75
2.EMPLOY1	.09582	.08664	2.746	.08946	.08146	2.877
3.EMPLOY1	.02873	.02791	5.643	.02125	.0208	6.639
4.EMPLOY1	.02159	.02112	6.584	.02097	.02053	6.687
5.EMPLOY1	.06177	.05796	3.641	.04851	.04616	4.203
6.EMPLOY1	.01968	.01929	6.917	.02728	.02654	5.804
7.EMPLOY1	.2904	.2061	.9237	.2989	.2096	.8786
8.EMPLOY1	.07008	.06517	3.368	.06379	.05973	3.57
9.EMPLOY1	.00631	.006271	12.47	.00725	.007198	11.62
2._INCOMG	.1365	.1179	2.118	.1316	.1142	2.18
3._INCOMG	.09158	.0832	2.832	.08317	.07626	3.019
4._INCOMG	.1226	.1076	2.301	.1177	.1039	2.372
5._INCOMG	.4442	.2469	.2246	.4471	.2472	.2128
9._INCOMG	.1338	.1159	2.151	.1427	.1223	2.044
2._CHLDCNT	.1021	.0917	2.628	.1066	.0952	2.55
3._CHLDCNT	.08469	.07752	2.983	.09188	.08344	2.826
4._CHLDCNT	.03645	.03512	4.947	.04004	.03844	4.692
5._CHLDCNT	.01719	.01689	7.43	.01592	.01566	7.736
6._CHLDCNT	.009797	.009702	9.954	.008949	.008869	10.43
9._CHLDCNT	.004151	.004135	15.42	.004855	.004832	14.25
2.marital	.1328	.1152	2.163	.1369	.1182	2.113
3.marital	.08303	.07614	3.022	.1238	.1085	2.284
4.marital	.01926	.01889	6.995	.01951	.01913	6.948
5.marital	.149	.1268	1.972	.1707	.1416	1.75
6.marital	.03396	.03281	5.146	.03201	.03098	5.318
9.marital	.00631	.006271	12.47	.005269	.005241	13.67
1.menthlth1	.1882	.1528	1.595	.1555	.1313	1.902
2.menthlth1	.06186	.05804	3.638	.04533	.04328	4.371
3.menthlth1	.04052	.03888	4.661	.03135	.03037	5.378
4.menthlth1	.02341	.02287	6.303	.01452	.01431	8.116
5.menthlth1	.009216	.009132	10.27	.005748	.005715	13.08
6.menthlth1	.07913	.07287	3.118	.04958	.04712	4.15
77.menthlth1	.01038	.01027	9.662	.01034	.01024	9.68
99.menthlth1	.003902	.003887	15.91	.00442	.0044	14.94
1.physhlth1	.2121	.1671	1.409	.1794	.1472	1.671
2.physhlth1	.06227	.0584	3.623	.04903	.04663	4.177
3.physhlth1	.0367	.03535	4.928	.03133	.03035	5.38
4.physhlth1	.01868	.01833	7.11	.01337	.01319	8.474
5.physhlth1	.01104	.01092	9.358	.007838	.007777	11.16
6.physhlth1	.08585	.07849	2.957	.07886	.07264	3.125
77.physhlth1	.01262	.01246	8.732	.01511	.01488	7.95
99.physhlth1	.003155	.003145	17.72	.004703	.004681	14.48
2._RFSMOK3	.1611	.1351	1.844	.125	.1094	2.267
9._RFSMOK3	.00548	.00545	13.4	.006205	.006167	12.58
2._PAINDX1	.4171	.2432	.3361	.4569	.2481	.1731
9._PAINDX1	.03412	.03296	5.132	.03593	.03464	4.987
2._RFBMI5	.6461	.2287	-.6112	.6145	.2369	-.4704
9._RFBMI5	.05762	.05431	3.797	.05916	.05566	3.737

After: _uw1aAlcohol as the weighting variable

	Caregivers (treat)			Non-caregivers (control)		
	mean	variance	skewness	mean	variance	skewness
2.sex	.6295	.2332	-.5364	.6295	.2332	-.5363
9.sex	.0004982	.000498	44.77	.0004982	.000498	44.77
_AGE80	56.14	241.1	-.5296	56.14	241.1	-.5295
2._RACEGR3	.0543	.05136	3.934	.0543	.05135	3.934
3._RACEGR3	.07962	.07329	3.106	.07963	.07329	3.106
4._RACEGR3	.05073	.04816	4.095	.05073	.04816	4.094
5._RACEGR3	.08212	.07538	3.044	.08212	.07538	3.044
9._RACEGR3	.01926	.01889	6.995	.01926	.01889	6.995
2._EDUCAG	.2384	.1816	1.228	.2384	.1816	1.228
3._EDUCAG	.3108	.2142	.8177	.3108	.2142	.8176
4._EDUCAG	.4021	.2404	.3993	.4021	.2404	.3993

9._EDUCAG	.001993	.001989	22.33	.001993	.001989	22.33
2.EMPLOY1	.09582	.08664	2.746	.09582	.08664	2.746
3.EMPLOY1	.02873	.02791	5.643	.02873	.0279	5.643
4.EMPLOY1	.02159	.02112	6.584	.02159	.02112	6.584
5.EMPLOY1	.06177	.05796	3.641	.06178	.05796	3.641
6.EMPLOY1	.01968	.01929	6.917	.01968	.01929	6.916
7.EMPLOY1	.2904	.2061	.9237	.2904	.2061	.9236
8.EMPLOY1	.07008	.06517	3.368	.07008	.06517	3.368
9.EMPLOY1	.00631	.006271	12.47	.006311	.006271	12.47
2._INCOMG	.1365	.1179	2.118	.1365	.1179	2.117
3._INCOMG	.09158	.0832	2.832	.09158	.0832	2.832
4._INCOMG	.1226	.1076	2.301	.1226	.1076	2.301
5._INCOMG	.4442	.2469	.2246	.4442	.2469	.2246
9._INCOMG	.1338	.1159	2.151	.1339	.1159	2.151
2._CHLDCNT	.1021	.0917	2.628	.1021	.0917	2.628
3._CHLDCNT	.08469	.07752	2.983	.08469	.07752	2.983
4._CHLDCNT	.03645	.03512	4.947	.03645	.03512	4.947
5._CHLDCNT	.01719	.01689	7.43	.01719	.01689	7.43
6._CHLDCNT	.009797	.009702	9.954	.009798	.009702	9.954
9._CHLDCNT	.004151	.004135	15.42	.004152	.004135	15.42
2.marital	.1328	.1152	2.163	.1329	.1152	2.163
3.marital	.08303	.07614	3.022	.08304	.07614	3.022
4.marital	.01926	.01889	6.995	.01926	.01889	6.995
5.marital	.149	.1268	1.972	.149	.1268	1.972
6.marital	.03396	.03281	5.146	.03396	.03281	5.146
9.marital	.00631	.006271	12.47	.00631	.006271	12.47
1.menthlth1	.1882	.1528	1.595	.1882	.1528	1.595
2.menthlth1	.06186	.05804	3.638	.06186	.05803	3.638
3.menthlth1	.04052	.03888	4.661	.04052	.03888	4.661
4.menthlth1	.02341	.02287	6.303	.02341	.02287	6.303
5.menthlth1	.009216	.009132	10.27	.009216	.009132	10.27
6.menthlth1	.07913	.07287	3.118	.07913	.07287	3.118
77.menthlth1	.01038	.01027	9.662	.01038	.01027	9.662
99.menthlth1	.003902	.003887	15.91	.003903	.003887	15.91
1.physhlth1	.2121	.1671	1.409	.2121	.1671	1.409
2.physhlth1	.06227	.0584	3.623	.06227	.0584	3.623
3.physhlth1	.0367	.03535	4.928	.0367	.03535	4.928
4.physhlth1	.01868	.01833	7.11	.01868	.01833	7.11
5.physhlth1	.01104	.01092	9.358	.01104	.01092	9.358
6.physhlth1	.08585	.07849	2.957	.08585	.07848	2.957
77.physhlth1	.01262	.01246	8.732	.01262	.01246	8.732
99.physhlth1	.003155	.003145	17.72	.003155	.003146	17.72
2._RFSMOK3	.1611	.1351	1.844	.1611	.1351	1.844
9._RFSMOK3	.00548	.00545	13.4	.00548	.00545	13.4
2._PAINDX1	.4171	.2432	.3361	.4171	.2431	.336
9._PAINDX1	.03412	.03296	5.132	.03413	.03296	5.132
2._RFBMI5	.6461	.2287	-.6112	.6461	.2287	-.6111
9._RFBMI5	.05762	.05431	3.797	.05762	.0543	3.797

Table A6 Covariate distribution before and after entropy balancing (caregivers versus non-caregivers) – Analysis of alcohol consumption, age group 18-49

Treated units: 3534 total of weights: 3534
Control units: 16016 total of weights: 3534

Before: without weighting

	Caregivers (treat)			Non-caregivers (control)		
	mean	variance	skewness	mean	variance	skewness
_AGE80	36.25	80.8	-.3605	34.93	81.24	-.1653
2.sex	.6109	.2378	-.455	.4971	.25	.01149
9.sex	.0008489	.0008484	34.28	.0006868	.0006864	38.12
2._RACEGR3	.06933	.06454	3.391	.05882	.05536	3.75
3._RACEGR3	.1075	.09599	2.534	.1123	.09971	2.455
4._RACEGR3	.0648	.06062	3.536	.06325	.05925	3.589
5._RACEGR3	.144	.1233	2.028	.1517	.1287	1.942
9._RACEGR3	.02179	.02132	6.551	.01467	.01446	8.073
2._EDUCAG	.2688	.1966	1.043	.2537	.1893	1.132
3._EDUCAG	.3257	.2197	.7439	.278	.2007	.9909
4._EDUCAG	.3469	.2266	.6432	.4027	.2405	.397

9._EDUCAG	.001981	.001977	22.4	.003309	.003298	17.3
2.EMPLOY1	.09451	.0856	2.772	.08829	.0805	2.902
3.EMPLOY1	.0382	.03675	4.818	.02348	.02293	6.294
4.EMPLOY1	.04244	.04065	4.539	.03571	.03444	5.004
5.EMPLOY1	.08885	.08098	2.89	.06213	.05827	3.628
6.EMPLOY1	.06282	.05889	3.604	.07561	.0699	3.21
7.EMPLOY1	.005659	.005629	13.18	.004995	.00497	14.04
8.EMPLOY1	.06112	.0574	3.664	.04133	.03963	4.608
9.EMPLOY1	.008489	.008419	10.71	.01005	.009952	9.823
2._INCOMG	.1607	.1349	1.848	.1277	.1114	2.231
3._INCOMG	.09253	.08399	2.812	.07686	.07096	3.177
4._INCOMG	.1143	.1013	2.424	.1127	.1	2.45
5._INCOMG	.416	.243	.341	.4877	.2499	.04922
9._INCOMG	.1205	.106	2.331	.1236	.1083	2.288
2._CHLDCNT	.187	.1521	1.605	.1987	.1593	1.51
3._CHLDCNT	.2162	.1695	1.379	.2096	.1657	1.427
4._CHLDCNT	.09989	.08993	2.669	.09684	.08747	2.726
5._CHLDCNT	.05037	.04784	4.112	.03915	.03762	4.752
6._CHLDCNT	.03028	.02937	5.483	.02173	.02126	6.561
9._CHLDCNT	.007074	.007026	11.76	.009241	.009156	10.26
2.marital	.09989	.08993	2.669	.08498	.07776	2.977
3.marital	.006791	.006747	12.01	.007055	.007006	11.78
4.marital	.02688	.02617	5.85	.02498	.02435	6.088
5.marital	.3161	.2162	.7912	.3253	.2195	.7458
6.marital	.06565	.06136	3.508	.06275	.05882	3.606
9.marital	.007074	.007026	11.76	.00512	.005094	13.87
1.menthlth1	.2261	.175	1.31	.211	.1665	1.417
2.menthlth1	.08942	.08144	2.878	.06369	.05963	3.574
3.menthlth1	.0614	.05765	3.654	.04421	.04225	4.435
4.menthlth1	.03452	.03334	5.099	.01917	.0188	7.013
5.menthlth1	.01217	.01202	8.899	.008304	.008236	10.84
6.menthlth1	.1101	.09799	2.492	.05526	.05221	3.893
77.menthlth1	.009904	.009808	9.899	.008117	.008052	10.96
99.menthlth1	.002547	.002541	19.74	.002622	.002616	19.45
1.physhlth1	.2459	.1855	1.18	.2119	.167	1.41
2.physhlth1	.06706	.06258	3.462	.04508	.04305	4.385
3.physhlth1	.04018	.03858	4.683	.02348	.02293	6.294
4.physhlth1	.01811	.01779	7.228	.009491	.009401	10.12
5.physhlth1	.01245	.0123	8.794	.005557	.005526	13.3
6.physhlth1	.06933	.06454	3.391	.0414	.03968	4.604
77.physhlth1	.01075	.01064	9.487	.009053	.008972	10.37
99.physhlth1	.002264	.002259	20.95	.002935	.002926	18.38
2._RFSMOK3	.2357	.1802	1.245	.153	.1296	1.927
9._RFSMOK3	.005376	.005349	13.53	.004808	.004785	14.32
2._PAINDX1	.4287	.245	.2882	.4841	.2498	.06347
9._PAINDX1	.03282	.03176	5.244	.03109	.03013	5.403
2._RFBMI5	.6208	.2355	-.4981	.5755	.2443	-.3054
9._RFBMI5	.06508	.06086	3.526	.06993	.06504	3.373

After: _uw1aAlcoholB1 as the weighting variable

	Caregivers (treat)			Non-caregivers (control)		
	mean	variance	skewness	mean	variance	skewness
_AGE80	36.25	80.8	-.3605	36.25	80.79	-.3599
2.sex	.6109	.2378	-.455	.6109	.2377	-.4548
9.sex	.0008489	.0008484	34.28	.000849	.0008483	34.28
2._RACEGR3	.06933	.06454	3.391	.06933	.06453	3.391
3._RACEGR3	.1075	.09599	2.534	.1076	.09599	2.533
4._RACEGR3	.0648	.06062	3.536	.06481	.06061	3.536
5._RACEGR3	.144	.1233	2.028	.1441	.1233	2.027
9._RACEGR3	.02179	.02132	6.551	.02179	.02132	6.551
2._EDUCAG	.2688	.1966	1.043	.2689	.1966	1.043
3._EDUCAG	.3257	.2197	.7439	.3257	.2197	.7436
4._EDUCAG	.3469	.2266	.6432	.347	.2266	.643
9._EDUCAG	.001981	.001977	22.4	.001981	.001978	22.4
2.EMPLOY1	.09451	.0856	2.772	.09452	.08559	2.772
3.EMPLOY1	.0382	.03675	4.818	.0382	.03675	4.818
4.EMPLOY1	.04244	.04065	4.539	.04245	.04065	4.539
5.EMPLOY1	.08885	.08098	2.89	.08886	.08097	2.89
6.EMPLOY1	.06282	.05889	3.604	.06283	.05888	3.603

7.EMPLOY1	.005659	.005629	13.18	.00566	.005628	13.18
8.EMPLOY1	.06112	.0574	3.664	.06113	.05739	3.664
9.EMPLOY1	.008489	.008419	10.71	.00849	.008419	10.71
2._INCOMG	.1607	.1349	1.848	.1608	.1349	1.847
3._INCOMG	.09253	.08399	2.812	.09254	.08398	2.812
4._INCOMG	.1143	.1013	2.424	.1143	.1013	2.424
5._INCOMG	.416	.243	.341	.416	.243	.3409
9._INCOMG	.1205	.106	2.331	.1206	.106	2.33
2._CHLDCNT	.187	.1521	1.605	.1871	.1521	1.605
3._CHLDCNT	.2162	.1695	1.379	.2162	.1695	1.379
4._CHLDCNT	.09989	.08993	2.669	.0999	.08992	2.669
5._CHLDCNT	.05037	.04784	4.112	.05037	.04784	4.112
6._CHLDCNT	.03028	.02937	5.483	.03028	.02937	5.482
9._CHLDCNT	.007074	.007026	11.76	.007075	.007026	11.76
2.marital	.09989	.08993	2.669	.0999	.08992	2.669
3.marital	.006791	.006747	12.01	.006792	.006746	12.01
4.marital	.02688	.02617	5.85	.02689	.02616	5.85
5.marital	.3161	.2162	.7912	.3161	.2162	.7909
6.marital	.06565	.06136	3.508	.06566	.06135	3.507
9.marital	.007074	.007026	11.76	.007075	.007025	11.76
1.menthlth1	.2261	.175	1.31	.2261	.175	1.309
2.menthlth1	.08942	.08144	2.878	.08943	.08143	2.878
3.menthlth1	.0614	.05765	3.654	.06141	.05764	3.654
4.menthlth1	.03452	.03334	5.099	.03453	.03334	5.099
5.menthlth1	.01217	.01202	8.899	.01217	.01202	8.899
6.menthlth1	.1101	.09799	2.492	.1101	.09798	2.491
77.menthlth1	.009904	.009808	9.899	.009905	.009808	9.898
99.menthlth1	.002547	.002541	19.74	.002547	.002541	19.74
1.physhlth1	.2459	.1855	1.18	.246	.1855	1.18
2.physhlth1	.06706	.06258	3.462	.06707	.06258	3.461
3.physhlth1	.04018	.03858	4.683	.04019	.03857	4.683
4.physhlth1	.01811	.01779	7.228	.01811	.01779	7.227
5.physhlth1	.01245	.0123	8.794	.01245	.0123	8.793
6.physhlth1	.06933	.06454	3.391	.06933	.06453	3.391
77.physhlth1	.01075	.01064	9.487	.01075	.01064	9.487
99.physhlth1	.002264	.002259	20.95	.002264	.002259	20.94
2._RFSMOK3	.2357	.1802	1.245	.2358	.1802	1.245
9._RFSMOK3	.005376	.005349	13.53	.005377	.005348	13.53
2._PAINDX1	.4287	.245	.2882	.4287	.2449	.288
9._PAINDX1	.03282	.03176	5.244	.03283	.03175	5.244
2._RFBMI5	.6208	.2355	-.4981	.6208	.2354	-.4978
9._RFBMI5	.06508	.06086	3.526	.06509	.06086	3.526

Table A7 Covariate distribution before and after entropy balancing (caregivers versus non-caregivers) – Analysis of alcohol consumption, age group 50-64

Treated units: 4498 total of weights: 4498
Control units: 13485 total of weights: 4498

Before: without weighting

	Caregivers (treat)			Non-caregivers (control)		
	mean	variance	skewness	mean	variance	skewness
_AGE80	57.48	17.87	-.1272	57.52	18.47	-.154
2.sex	.6383	.2309	-.5756	.5281	.2492	-.1127
9.sex	.0004446	.0004445	47.39	.0004449	.0004448	47.38
2._RACEGR3	.05669	.05349	3.834	.05428	.05134	3.934
3._RACEGR3	.07603	.07027	3.199	.08291	.07604	3.025
4._RACEGR3	.05202	.04933	4.034	.03797	.03653	4.835
5._RACEGR3	.0687	.06399	3.41	.0795	.07318	3.109
9._RACEGR3	.01801	.01769	7.249	.02054	.02012	6.76
2._EDUCAG	.2314	.1779	1.274	.2538	.1894	1.131
3._EDUCAG	.3126	.2149	.8086	.2682	.1963	1.046
4._EDUCAG	.4142	.2427	.3484	.4148	.2428	.3457
9._EDUCAG	.002223	.002219	21.14	.003634	.003621	16.5
2.EMPLOY1	.1189	.1048	2.354	.1235	.1082	2.289
3.EMPLOY1	.03446	.03328	5.104	.03137	.03039	5.377
4.EMPLOY1	.02112	.02068	6.661	.02314	.0226	6.344
5.EMPLOY1	.05024	.04773	4.118	.03574	.03447	5.001
6.EMPLOY1	.002223	.002219	21.14	.002373	.002368	20.46

7.EMPLOY1	.1378	.1189	2.101	.1372	.1184	2.109
8.EMPLOY1	.1152	.1019	2.411	.1201	.1057	2.338
9.EMPLOY1	.00578	.005748	13.04	.007638	.00758	11.31
2._INCOMG	.1127	.1	2.449	.1126	.09991	2.452
3._INCOMG	.07559	.06989	3.211	.06415	.06004	3.558
4._INCOMG	.108	.0964	2.525	.099	.0892	2.685
5._INCOMG	.5109	.2499	-.04359	.5126	.2499	-.05059
9._INCOMG	.1169	.1033	2.384	.1218	.107	2.312
2._CHLDCNT	.09916	.08934	2.682	.09907	.08926	2.684
3._CHLDCNT	.04624	.04411	4.321	.05161	.04895	4.053
4._CHLDCNT	.01556	.01532	7.828	.01631	.01605	7.636
5._CHLDCNT	.004891	.004868	14.19	.005562	.005531	13.3
6._CHLDCNT	.002223	.002219	21.14	.004079	.004062	15.56
9._CHLDCNT	.004002	.003987	15.71	.004598	.004577	14.65
2.marital	.1621	.1358	1.834	.1814	.1485	1.654
3.marital	.05225	.04953	4.024	.0571	.05384	3.818
4.marital	.02068	.02025	6.737	.02351	.02296	6.29
5.marital	.1085	.09674	2.518	.1167	.1031	2.387
6.marital	.02823	.02744	5.696	.02306	.02253	6.355
9.marital	.007559	.007503	11.37	.006377	.006337	12.4
1.menthlth1	.1903	.1541	1.578	.1463	.1249	2.002
2.menthlth1	.0558	.0527	3.87	.04234	.04055	4.545
3.menthlth1	.04024	.03863	4.679	.03026	.02934	5.485
4.menthlth1	.02223	.02174	6.481	.01624	.01598	7.655
5.menthlth1	.009782	.009689	9.962	.005784	.005751	13.03
6.menthlth1	.08137	.07477	3.062	.05895	.05548	3.745
77.menthlth1	.009115	.009034	10.33	.0106	.01049	9.556
99.menthlth1	.004891	.004868	14.19	.005562	.005531	13.3
1.physhlth1	.209	.1653	1.432	.1741	.1438	1.719
2.physhlth1	.06069	.05702	3.68	.04968	.04722	4.145
3.physhlth1	.03668	.03535	4.929	.03426	.03309	5.121
4.physhlth1	.02268	.02217	6.413	.01594	.01569	7.729
5.physhlth1	.01089	.01078	9.424	.00964	.009548	10.04
6.physhlth1	.09738	.08791	2.716	.1004	.09033	2.659
77.physhlth1	.009782	.009689	9.962	.01172	.01158	9.075
99.physhlth1	.00289	.002882	18.52	.004375	.004356	15.02
2._RFSMOK3	.1739	.1437	1.721	.1498	.1274	1.963
9._RFSMOK3	.005113	.005088	13.88	.005636	.005605	13.21
2._PAINDX1	.4211	.2438	.3197	.4584	.2483	.1668
9._PAINDX1	.03779	.03637	4.847	.03878	.03728	4.777
2._RFBMI5	.6654	.2227	-.7011	.6666	.2223	-.7068
9._RFBMI5	.06247	.05858	3.616	.05858	.05516	3.759

After: _uw1aAlcoholB2 as the weighting variable

		Caregivers (treat)			Non-caregivers (control)	
	mean	variance	skewness	mean	variance	skewness
_AGE80	57.48	17.87	-.1272	57.48	17.87	-.1259
2.sex	.6383	.2309	-.5756	.6382	.2309	-.5754
9.sex	.0004446	.0004445	47.39	.0004447	.0004445	47.39
2._RACEGR3	.05669	.05349	3.834	.0567	.05349	3.834
3._RACEGR3	.07603	.07027	3.199	.07604	.07026	3.199
4._RACEGR3	.05202	.04933	4.034	.05203	.04932	4.034
5._RACEGR3	.0687	.06399	3.41	.0687	.06399	3.41
9._RACEGR3	.01801	.01769	7.249	.01801	.01769	7.249
2._EDUCAG	.2314	.1779	1.274	.2315	.1779	1.273
3._EDUCAG	.3126	.2149	.8086	.3126	.2149	.8084
4._EDUCAG	.4142	.2427	.3484	.4142	.2427	.3483
9._EDUCAG	.002223	.002219	21.14	.002223	.002219	21.14
2.EMPLOY1	.1189	.1048	2.354	.119	.1048	2.354
3.EMPLOY1	.03446	.03328	5.104	.03446	.03328	5.104
4.EMPLOY1	.02112	.02068	6.661	.02112	.02068	6.661
5.EMPLOY1	.05024	.04773	4.118	.05025	.04773	4.118
6.EMPLOY1	.002223	.002219	21.14	.002223	.002219	21.14
7.EMPLOY1	.1378	.1189	2.101	.1379	.1189	2.101
8.EMPLOY1	.1152	.1019	2.411	.1152	.1019	2.411
9.EMPLOY1	.00578	.005748	13.04	.005781	.005748	13.04
2._INCOMG	.1127	.1	2.449	.1127	.1	2.449
3._INCOMG	.07559	.06989	3.211	.07559	.06989	3.211
4._INCOMG	.108	.0964	2.525	.1081	.09639	2.525

5._INCOMG	.5109	.2499	-.04359	.5109	.2499	-.04357
9._INCOMG	.1169	.1033	2.384	.117	.1033	2.384
2._CHLDCNT	.09916	.08934	2.682	.09916	.08934	2.682
3._CHLDCNT	.04624	.04411	4.321	.04625	.04411	4.321
4._CHLDCNT	.01556	.01532	7.828	.01556	.01532	7.827
5._CHLDCNT	.004891	.004868	14.19	.004892	.004868	14.19
6._CHLDCNT	.002223	.002219	21.14	.002224	.002219	21.14
9._CHLDCNT	.004002	.003987	15.71	.004002	.003986	15.71
2.marital	.1621	.1358	1.834	.1621	.1358	1.834
3.marital	.05225	.04953	4.024	.05225	.04952	4.024
4.marital	.02068	.02025	6.737	.02068	.02025	6.737
5.marital	.1085	.09674	2.518	.1085	.09674	2.517
6.marital	.02823	.02744	5.696	.02824	.02744	5.696
9.marital	.007559	.007503	11.37	.00756	.007503	11.37
1.menthlth1	.1903	.1541	1.578	.1903	.1541	1.578
2.menthlth1	.0558	.0527	3.87	.05581	.0527	3.87
3.menthlth1	.04024	.03863	4.679	.04024	.03863	4.679
4.menthlth1	.02223	.02174	6.481	.02223	.02174	6.481
5.menthlth1	.009782	.009689	9.962	.009783	.009688	9.961
6.menthlth1	.08137	.07477	3.062	.08138	.07476	3.062
77.menthlth1	.009115	.009034	10.33	.009116	.009034	10.33
99.menthlth1	.004891	.004868	14.19	.004892	.004868	14.19
1.physhlth1	.209	.1653	1.432	.209	.1653	1.431
2.physhlth1	.06069	.05702	3.68	.0607	.05702	3.68
3.physhlth1	.03668	.03535	4.929	.03669	.03534	4.929
4.physhlth1	.02268	.02217	6.413	.02268	.02217	6.412
5.physhlth1	.01089	.01078	9.424	.01089	.01078	9.423
6.physhlth1	.09738	.08791	2.716	.09738	.08791	2.716
77.physhlth1	.009782	.009689	9.962	.009783	.009688	9.961
99.physhlth1	.00289	.002882	18.52	.00289	.002882	18.52
2._RFSMOK3	.1739	.1437	1.721	.1739	.1437	1.721
9._RFSMOK3	.005113	.005088	13.88	.005114	.005088	13.88
2._PAINDX1	.4211	.2438	.3197	.4211	.2438	.3196
9._PAINDX1	.03779	.03637	4.847	.0378	.03637	4.847
2._RFBMI5	.6654	.2227	-.7011	.6654	.2227	-.7009
9._RFBMI5	.06247	.05858	3.616	.06248	.05858	3.616

Table A8 Covariate distribution before and after entropy balancing (caregivers versus non-caregivers) – Analysis of alcohol consumption, age group 65+

Treated units: 4012 total of weights: 4012
Control units: 16428 total of weights: 4012

Before: without weighting

	Caregivers (treat)			Non-caregivers (control)		
	mean	variance	skewness	mean	variance	skewness
_AGE80	72.15	25.84	.2641	73.04	27.68	.02282
2.sex	.6361	.2315	-.5657	.5862	.2426	-.35
9.sex	.0002493	.0002493	63.32	.0005478	.0005476	42.69
2._RACEGR3	.03838	.03692	4.805	.04042	.03879	4.667
3._RACEGR3	.05907	.0556	3.74	.07	.06511	3.371
4._RACEGR3	.03689	.03554	4.914	.02916	.02831	5.597
5._RACEGR3	.04262	.04082	4.528	.04663	.04446	4.301
9._RACEGR3	.01844	.01811	7.158	.02021	.0198	6.819
2._EDUCAG	.2193	.1713	1.356	.265	.1948	1.065
3._EDUCAG	.2956	.2083	.8958	.2661	.1953	1.059
4._EDUCAG	.4372	.2461	.2533	.4021	.2404	.3995
9._EDUCAG	.001745	.001742	23.88	.003652	.003639	16.46
2.EMPLOY1	.07104	.06601	3.34	.0627	.05877	3.608
3.EMPLOY1	.01396	.01377	8.286	.01077	.01066	9.478
4.EMPLOY1	.003739	.003726	16.26	.004809	.004786	14.32
5.EMPLOY1	.05085	.04827	4.089	.04571	.04363	4.35
6.EMPLOY1	.001246	.001245	28.27	.0006087	.0006084	40.49
7.EMPLOY1	.7121	.2051	-.9369	.7182	.2024	-.9698
8.EMPLOY1	.02742	.02667	5.788	.03951	.03795	4.728
9.EMPLOY1	.004985	.004961	14.06	.0042	.004183	15.33
2._INCOMG	.1418	.1217	2.053	.1509	.1281	1.951
3._INCOMG	.1087	.09689	2.515	.1049	.09394	2.578
4._INCOMG	.1463	.1249	2.002	.1381	.119	2.098

5._INCOMG	.3943	.2389	.4325	.3537	.2286	.6119
9._INCOMG	.1645	.1375	1.81	.1784	.1466	1.68
2._CHLDCNT	.03066	.02973	5.445	.02283	.02231	6.39
3._CHLDCNT	.01196	.01182	8.977	.01017	.01006	9.766
4._CHLDCNT	.003988	.003973	15.74	.004139	.004122	15.45
5._CHLDCNT	.001745	.001742	23.88	.001765	.001762	23.74
6._CHLDCNT	.0002493	.0002493	63.32	.000487	.0004868	45.28
9._CHLDCNT	.001745	.001742	23.88	.0007913	.0007908	35.51
2.marital	.1291	.1125	2.212	.151	.1282	1.949
3.marital	.1847	.1506	1.625	.2924	.2069	.913
4.marital	.01097	.01085	9.391	.0109	.01078	9.423
5.marital	.04711	.0449	4.275	.0644	.06026	3.549
6.marital	.01246	.01231	8.789	.009374	.009287	10.18
9.marital	.004237	.00422	15.26	.004505	.004484	14.8
1.menthlth1	.1525	.1293	1.933	.1089	.09705	2.511
2.menthlth1	.04437	.04241	4.426	.02989	.029	5.522
3.menthlth1	.02243	.02193	6.45	.01972	.01933	6.908
4.menthlth1	.01496	.01474	7.993	.008583	.00851	10.65
5.menthlth1	.005982	.005948	12.81	.003226	.003216	17.52
6.menthlth1	.04935	.04693	4.161	.03634	.03502	4.955
77.menthlth1	.01221	.01207	8.882	.0123	.01215	8.851
99.menthlth1	.003988	.003973	15.74	.005235	.005208	13.71
1.physhlth1	.1857	.1512	1.617	.1519	.1289	1.939
2.physhlth1	.05982	.05626	3.712	.05235	.04961	4.02
3.physhlth1	.03365	.03252	5.172	.03658	.03525	4.937
4.physhlth1	.01471	.01449	8.063	.01504	.01481	7.97
5.physhlth1	.00997	.009873	9.865	.008583	.00851	10.65
6.physhlth1	.08749	.07985	2.92	.0977	.08816	2.71
77.physhlth1	.01745	.01715	7.371	.0238	.02324	6.248
99.physhlth1	.004237	.00422	15.26	.006696	.006651	12.1
2._RFSMOK3	.08101	.07446	3.071	.07743	.07144	3.162
9._RFSMOK3	.005982	.005948	12.81	.008035	.007971	11.02
2._PAINDX1	.4025	.2406	.3975	.429	.245	.2868
9._PAINDX1	.03116	.03019	5.397	.03829	.03682	4.812
2._RFBMI5	.6468	.2285	-.6143	.6097	.238	-.4497
9._RFBMI5	.04561	.04354	4.356	.04912	.04671	4.172

After: _uw1aAlcoholB3 as the weighting variable

	Caregivers (treat)			Non-caregivers (control)		
	mean	variance	skewness	mean	variance	skewness
_AGE80	72.15	25.84	.2641	72.14	25.84	.266
2.sex	.6361	.2315	-.5657	.636	.2315	-.5655
9.sex	.0002493	.0002493	63.32	.0002495	.0002494	63.29
2._RACEGR3	.03838	.03692	4.805	.03839	.03692	4.805
3._RACEGR3	.05907	.0556	3.74	.05908	.05559	3.74
4._RACEGR3	.03689	.03554	4.914	.03689	.03553	4.914
5._RACEGR3	.04262	.04082	4.528	.04263	.04081	4.528
9._RACEGR3	.01844	.01811	7.158	.01845	.01811	7.157
2._EDUCAG	.2193	.1713	1.356	.2194	.1713	1.356
3._EDUCAG	.2956	.2083	.8958	.2957	.2083	.8956
4._EDUCAG	.4372	.2461	.2533	.4372	.2461	.2532
9._EDUCAG	.001745	.001742	23.88	.001746	.001743	23.87
2.EMPLOY1	.07104	.06601	3.34	.07104	.066	3.34
3.EMPLOY1	.01396	.01377	8.286	.01396	.01377	8.286
4.EMPLOY1	.003739	.003726	16.26	.003739	.003726	16.26
5.EMPLOY1	.05085	.04827	4.089	.05085	.04827	4.089
6.EMPLOY1	.001246	.001245	28.27	.001246	.001245	28.27
7.EMPLOY1	.7121	.2051	-.9369	.7121	.205	-.9367
8.EMPLOY1	.02742	.02667	5.788	.02742	.02667	5.787
9.EMPLOY1	.004985	.004961	14.06	.004986	.004961	14.06
2._INCOMG	.1418	.1217	2.053	.1419	.1217	2.053
3._INCOMG	.1087	.09689	2.515	.1087	.09689	2.514
4._INCOMG	.1463	.1249	2.002	.1463	.1249	2.001
5._INCOMG	.3943	.2389	.4325	.3943	.2389	.4324
9._INCOMG	.1645	.1375	1.81	.1645	.1375	1.81
2._CHLDCNT	.03066	.02973	5.445	.03066	.02972	5.445
3._CHLDCNT	.01196	.01182	8.977	.01197	.01182	8.977
4._CHLDCNT	.003988	.003973	15.74	.003989	.003973	15.74
5._CHLDCNT	.001745	.001742	23.88	.001745	.001742	23.88

6._CHLDCNT	.0002493	.0002493	63.32	.0002493	.0002493	63.3
9._CHLDCNT	.001745	.001742	23.88	.001745	.001742	23.88
2.marital	.1291	.1125	2.212	.1291	.1125	2.212
3.marital	.1847	.1506	1.625	.1847	.1506	1.625
4.marital	.01097	.01085	9.391	.01097	.01085	9.391
5.marital	.04711	.0449	4.275	.04711	.0449	4.275
6.marital	.01246	.01231	8.789	.01246	.01231	8.789
9.marital	.004237	.00422	15.26	.004238	.00422	15.26
1.menthlth1	.1525	.1293	1.933	.1526	.1293	1.932
2.menthlth1	.04437	.04241	4.426	.04437	.0424	4.425
3.menthlth1	.02243	.02193	6.45	.02244	.02193	6.45
4.menthlth1	.01496	.01474	7.993	.01496	.01473	7.992
5.menthlth1	.005982	.005948	12.81	.005983	.005947	12.81
6.menthlth1	.04935	.04693	4.161	.04936	.04692	4.161
77.menthlth1	.01221	.01207	8.882	.01221	.01207	8.881
99.menthlth1	.003988	.003973	15.74	.003989	.003973	15.74
1.physhlth1	.1857	.1512	1.617	.1857	.1512	1.616
2.physhlth1	.05982	.05626	3.712	.05983	.05625	3.712
3.physhlth1	.03365	.03252	5.172	.03365	.03252	5.172
4.physhlth1	.01471	.01449	8.063	.01471	.01449	8.063
5.physhlth1	.00997	.009873	9.865	.009971	.009872	9.864
6.physhlth1	.08749	.07985	2.92	.0875	.07985	2.92
77.physhlth1	.01745	.01715	7.371	.01745	.01715	7.37
99.physhlth1	.004237	.00422	15.26	.004238	.004221	15.26
2._RFSMOK3	.08101	.07446	3.071	.08101	.07446	3.071
9._RFSMOK3	.005982	.005948	12.81	.005983	.005948	12.81
2._PAINDX1	.4025	.2406	.3975	.4026	.2405	.3973
9._PAINDX1	.03116	.03019	5.397	.03116	.03019	5.397
2._RFBMI5	.6468	.2285	-.6143	.6468	.2285	-.6141
9._RFBMI5	.04561	.04354	4.356	.04562	.04354	4.355

Table A9 Covariate distribution before and after entropy balancing (caregivers versus non-caregivers) – Analysis of tobacco use, all age groups

Treated units: 11933 total of weights: 11933
Control units: 45416 total of weights: 11933

Before: without weighting

	Caregivers (treat)			Non-caregivers (control)		
	mean	variance	skewness	mean	variance	skewness
2.sex	.6295	.2332	-.5364	.539	.2485	-.1564
9.sex	.0005028	.0005026	44.56	.0004404	.0004402	47.62
_AGE80	56.15	241	-.5299	55.19	302.3	-.3922
2._RACEGR3	.05447	.05151	3.926	.05082	.04824	4.09
3._RACEGR3	.07969	.07335	3.104	.08865	.08079	2.894
4._RACEGR3	.0507	.04813	4.096	.04375	.04184	4.461
5._RACEGR3	.08238	.0756	3.038	.09261	.08404	2.811
9._RACEGR3	.01869	.01834	7.108	.01803	.01771	7.244
2._EDUCAG	.238	.1814	1.23	.2569	.1909	1.113
3._EDUCAG	.3107	.2142	.8179	.2715	.1978	1.028
4._EDUCAG	.4027	.2405	.3969	.4067	.2413	.3798
9._EDUCAG	.001927	.001924	22.71	.003281	.00327	17.37
2.EMPLOY1	.09553	.08641	2.752	.08944	.08144	2.877
3.EMPLOY1	.02866	.02784	5.65	.02129	.02084	6.632
4.EMPLOY1	.02154	.02107	6.592	.02072	.02029	6.729
5.EMPLOY1	.06176	.05795	3.641	.04853	.04618	4.202
6.EMPLOY1	.01961	.01923	6.929	.02728	.02654	5.804
7.EMPLOY1	.2904	.2061	.9236	.2989	.2095	.8788
8.EMPLOY1	.06989	.06501	3.374	.0637	.05964	3.573
9.EMPLOY1	.006117	.006081	12.67	.006914	.006866	11.9
2._INCOMG	.1362	.1176	2.122	.1316	.1143	2.18
3._INCOMG	.09185	.08342	2.826	.08327	.07634	3.017
4._INCOMG	.1226	.1076	2.301	.1179	.104	2.37
5._INCOMG	.4462	.2471	.2166	.4491	.2474	.2047
9._INCOMG	.1317	.1143	2.179	.1403	.1206	2.071
2._CHLDCNT	.102	.09159	2.63	.1067	.09528	2.549
3._CHLDCNT	.08506	.07783	2.975	.09197	.08351	2.824
4._CHLDCNT	.03654	.03521	4.94	.04029	.03867	4.675

5._CHLDCNT	.01726	.01697	7.412	.01603	.01577	7.707
6._CHLDCNT	.009721	.009627	9.994	.008852	.008773	10.49
9._CHLDCNT	.004022	.004007	15.67	.004492	.004472	14.82
2.marital	.1328	.1152	2.164	.137	.1182	2.111
3.marital	.08296	.07609	3.024	.1237	.1084	2.286
4.marital	.01936	.01898	6.977	.01955	.01917	6.94
5.marital	.1485	.1265	1.977	.1707	.1416	1.751
6.marital	.03377	.03263	5.162	.03195	.03093	5.323
9.marital	.006034	.005998	12.76	.005086	.005061	13.91
1.menthlth1	.1886	.1531	1.592	.1559	.1316	1.897
2.menthlth1	.06201	.05817	3.632	.04562	.04354	4.355
3.menthlth1	.04073	.03907	4.647	.03149	.0305	5.366
4.menthlth1	.02355	.023	6.284	.01451	.0143	8.12
5.menthlth1	.009302	.009216	10.22	.005791	.005758	13.03
6.menthlth1	.07886	.07264	3.125	.04928	.04685	4.165
77.menthlth1	.009972	.009874	9.863	.009996	.009897	9.851
99.menthlth1	.003771	.003757	16.19	.004338	.004319	15.08
1.physhlth1	.2129	.1676	1.403	.1799	.1476	1.666
2.physhlth1	.06235	.05847	3.62	.04915	.04673	4.171
3.physhlth1	.03645	.03513	4.947	.03142	.03043	5.372
4.physhlth1	.01877	.01842	7.092	.01339	.01321	8.468
5.physhlth1	.01115	.01102	9.313	.007905	.007842	11.11
6.physhlth1	.08573	.07839	2.959	.07878	.07258	3.127
77.physhlth1	.01215	.012	8.906	.01462	.01441	8.088
99.physhlth1	.003101	.003091	17.88	.004624	.004603	14.6
1.AlcCatb	.1461	.1248	2.003	.1483	.1263	1.979
2.AlcCatb	.04567	.04359	4.352	.04943	.04699	4.157
77.AlcCatb	.005196	.005169	13.76	.00665	.006606	12.14
99.AlcCatb	.001425	.001423	26.44	.001585	.001583	25.06
2._PAINDX1	.4174	.2432	.3349	.4563	.2481	.1754
9._PAINDX1	.03293	.03185	5.234	.03494	.03372	5.065
2._RFBMI5	.6466	.2285	-.6134	.6158	.2366	-.476
9._RFBMI5	.05657	.05337	3.839	.05802	.05465	3.781

After: _uw1aTobacco as the weighting variable

	Caregivers (treat)			Non-caregivers (control)		
	mean	variance	skewness	mean	variance	skewness
2.sex	.6295	.2332	-.5364	.6295	.2332	-.5363
9.sex	.0005028	.0005026	44.56	.0005028	.0005026	44.56
_AGE80	56.15	241	-.5299	56.15	241	-.5298
2._RACEGR3	.05447	.05151	3.926	.05447	.05151	3.926
3._RACEGR3	.07969	.07335	3.104	.0797	.07335	3.104
4._RACEGR3	.0507	.04813	4.096	.0507	.04813	4.096
5._RACEGR3	.08238	.0756	3.038	.08238	.0756	3.038
9._RACEGR3	.01869	.01834	7.108	.01869	.01834	7.108
2._EDUCAG	.238	.1814	1.23	.238	.1814	1.23
3._EDUCAG	.3107	.2142	.8179	.3107	.2142	.8178
4._EDUCAG	.4027	.2405	.3969	.4027	.2405	.3969
9._EDUCAG	.001927	.001924	22.71	.001928	.001924	22.71
2.EMPLOY1	.09553	.08641	2.752	.09554	.08641	2.752
3.EMPLOY1	.02866	.02784	5.65	.02866	.02784	5.65
4.EMPLOY1	.02154	.02107	6.592	.02154	.02107	6.592
5.EMPLOY1	.06176	.05795	3.641	.06176	.05795	3.641
6.EMPLOY1	.01961	.01923	6.929	.01961	.01923	6.929
7.EMPLOY1	.2904	.2061	.9236	.2904	.2061	.9235
8.EMPLOY1	.06989	.06501	3.374	.06989	.06501	3.374
9.EMPLOY1	.006117	.006081	12.67	.006118	.006081	12.67
2._INCOMG	.1362	.1176	2.122	.1362	.1176	2.121
3._INCOMG	.09185	.08342	2.826	.09185	.08341	2.826
4._INCOMG	.1226	.1076	2.301	.1226	.1076	2.301
5._INCOMG	.4462	.2471	.2166	.4462	.2471	.2166
9._INCOMG	.1317	.1143	2.179	.1317	.1143	2.179
2._CHLDCNT	.102	.09159	2.63	.102	.09159	2.63
3._CHLDCNT	.08506	.07783	2.975	.08506	.07783	2.975
4._CHLDCNT	.03654	.03521	4.94	.03654	.0352	4.94
5._CHLDCNT	.01726	.01697	7.412	.01726	.01697	7.412
6._CHLDCNT	.009721	.009627	9.994	.009721	.009627	9.994
9._CHLDCNT	.004022	.004007	15.67	.004023	.004007	15.67
2.marital	.1328	.1152	2.164	.1328	.1152	2.164

3.marital	.08296	.07609	3.024	.08297	.07609	3.024
4.marital	.01936	.01898	6.977	.01936	.01898	6.977
5.marital	.1485	.1265	1.977	.1485	.1265	1.977
6.marital	.03377	.03263	5.162	.03377	.03263	5.162
9.marital	.006034	.005998	12.76	.006034	.005998	12.76
1.menthlth1	.1886	.1531	1.592	.1886	.1531	1.592
2.menthlth1	.06201	.05817	3.632	.06201	.05817	3.632
3.menthlth1	.04073	.03907	4.647	.04073	.03907	4.647
4.menthlth1	.02355	.023	6.284	.02355	.02299	6.284
5.menthlth1	.009302	.009216	10.22	.009302	.009216	10.22
6.menthlth1	.07886	.07264	3.125	.07886	.07264	3.125
77.menthlth1	.009972	.009874	9.863	.009973	.009873	9.863
99.menthlth1	.003771	.003757	16.19	.003771	.003757	16.19
1.physhlth1	.2129	.1676	1.403	.2129	.1676	1.403
2.physhlth1	.06235	.05847	3.62	.06235	.05846	3.62
3.physhlth1	.03645	.03513	4.947	.03645	.03513	4.947
4.physhlth1	.01877	.01842	7.092	.01877	.01842	7.092
5.physhlth1	.01115	.01102	9.313	.01115	.01102	9.313
6.physhlth1	.08573	.07839	2.959	.08573	.07838	2.959
77.physhlth1	.01215	.012	8.906	.01215	.012	8.905
99.physhlth1	.003101	.003091	17.88	.003101	.003091	17.87
1.AlcCatb	.1461	.1248	2.003	.1462	.1248	2.003
2.AlcCatb	.04567	.04359	4.352	.04567	.04359	4.352
77.AlcCatb	.005196	.005169	13.76	.005196	.005169	13.76
99.AlcCatb	.001425	.001423	26.44	.001425	.001423	26.44
2._PAINDX1	.4174	.2432	.3349	.4174	.2432	.3349
9._PAINDX1	.03293	.03185	5.234	.03294	.03185	5.234
2._RFBMI5	.6466	.2285	-.6134	.6466	.2285	-.6133
9._RFBMI5	.05657	.05337	3.839	.05657	.05337	3.839

Table A10 Covariate distribution before and after entropy balancing (caregivers versus non-caregivers) – Analysis of tobacco use, age group 18-49

Treated units: 3494 total of weights: 3494
Control units: 15845 total of weights: 3494

Before: without weighting

	Caregivers (treat)			Non-caregivers (control)		
	mean	variance	skewness	mean	variance	skewness
_AGE80	36.25	80.75	-.3605	34.94	81.16	-.167
2.sex	.6116	.2376	-.458	.4979	.25	.008457
9.sex	.0008586	.0008581	34.08	.0004418	.0004416	47.55
2._RACEGR3	.07012	.06522	3.367	.05882	.05536	3.75
3._RACEGR3	.1073	.09584	2.537	.1125	.09987	2.452
4._RACEGR3	.0644	.06027	3.549	.06349	.05946	3.58
5._RACEGR3	.1448	.1239	2.019	.1508	.128	1.952
9._RACEGR3	.02089	.02046	6.7	.01452	.01431	8.118
2._EDUCAG	.2682	.1963	1.047	.2524	.1887	1.14
3._EDUCAG	.3251	.2195	.7466	.2785	.201	.9882
4._EDUCAG	.3483	.2271	.6368	.4038	.2408	.3919
9._EDUCAG	.001717	.001715	24.07	.003282	.003271	17.37
2.EMPLOY1	.09302	.08439	2.802	.08829	.0805	2.902
3.EMPLOY1	.03749	.0361	4.869	.02348	.02293	6.294
4.EMPLOY1	.04264	.04084	4.527	.03515	.03392	5.048
5.EMPLOY1	.08901	.08111	2.887	.06204	.05819	3.631
6.EMPLOY1	.06268	.05877	3.608	.07554	.06984	3.212
7.EMPLOY1	.004865	.004843	14.23	.004986	.004961	14.06
8.EMPLOY1	.06068	.05701	3.68	.0414	.03969	4.604
9.EMPLOY1	.008014	.007952	11.04	.009782	.009687	9.962
2._INCOMG	.16	.1344	1.855	.1278	.1115	2.23
3._INCOMG	.09273	.08416	2.808	.07681	.07091	3.179
4._INCOMG	.1151	.1018	2.413	.1128	.1001	2.448
5._INCOMG	.4184	.2434	.3307	.4896	.2499	.04154
9._INCOMG	.1173	.1036	2.378	.1215	.1067	2.317
2._CHLDCNT	.1866	.1518	1.609	.1986	.1592	1.511
3._CHLDCNT	.2175	.1703	1.369	.2098	.1658	1.425
4._CHLDCNT	.1002	.09016	2.663	.09738	.0879	2.716
5._CHLDCNT	.05066	.04811	4.098	.03957	.03801	4.724
6._CHLDCNT	.03005	.02916	5.505	.02146	.021	6.605

9._CHLDCNT	.006869	.006824	11.94	.008709	.008634	10.57
2.marital	.0996	.0897	2.674	.08514	.07789	2.973
3.marital	.006869	.006824	11.94	.007132	.007081	11.71
4.marital	.02719	.02646	5.814	.02493	.02431	6.094
5.marital	.3151	.2159	.796	.3251	.2194	.7468
6.marital	.06497	.06077	3.53	.06273	.0588	3.607
9.marital	.006869	.006824	11.94	.004923	.004899	14.15
1.menthlth1	.2272	.1757	1.302	.2112	.1666	1.415
2.menthlth1	.08958	.08158	2.874	.06418	.06007	3.557
3.menthlth1	.06211	.05827	3.629	.04443	.04246	4.422
4.menthlth1	.03463	.03344	5.09	.01919	.01882	7.01
5.menthlth1	.01231	.01216	8.847	.008331	.008262	10.82
6.menthlth1	.1093	.09741	2.504	.05484	.05184	3.91
77.menthlth1	.009731	.009639	9.989	.007826	.007765	11.17
99.menthlth1	.00229	.002285	20.83	.002524	.002518	19.83
1.physhlth1	.247	.186	1.173	.2124	.1673	1.406
2.physhlth1	.06783	.06325	3.437	.04519	.04315	4.379
3.physhlth1	.03921	.03768	4.748	.02354	.02299	6.285
4.physhlth1	.01832	.01799	7.184	.009467	.009378	10.13
5.physhlth1	.01259	.01244	8.742	.005617	.005586	13.23
6.physhlth1	.06869	.06399	3.411	.04146	.03975	4.6
77.physhlth1	.0103	.0102	9.699	.008709	.008634	10.57
99.physhlth1	.002003	.002	22.27	.002777	.002769	18.9
1.AlcCatb	.1895	.1536	1.585	.2008	.1605	1.494
2.AlcCatb	.08586	.07851	2.956	.08886	.08097	2.89
77.AlcCatb	.009445	.009358	10.14	.008583	.00851	10.65
99.AlcCatb	.001145	.001144	29.5	.001199	.001198	28.83
2._PAINDX1	.4284	.245	.2892	.4831	.2497	.06782
9._PAINDX1	.03234	.0313	5.287	.03029	.02938	5.481
2._RFBMI5	.6208	.2355	-.4979	.5761	.2442	-.3079
9._RFBMI5	.06497	.06077	3.53	.06923	.06444	3.394

After: _uw1aTobaccoB1 as the weighting variable

		Caregivers (treat)			Non-caregivers (control)	
	mean	variance	skewness	mean	variance	skewness
_AGE80	36.25	80.75	-.3605	36.25	80.75	-.36
2.sex	.6116	.2376	-.458	.6116	.2376	-.4578
9.sex	.0008586	.0008581	34.08	.0008587	.000858	34.08
2._RACEGR3	.07012	.06522	3.367	.07013	.06521	3.367
3._RACEGR3	.1073	.09584	2.537	.1074	.09583	2.537
4._RACEGR3	.0644	.06027	3.549	.0644	.06026	3.549
5._RACEGR3	.1448	.1239	2.019	.1449	.1239	2.018
9._RACEGR3	.02089	.02046	6.7	.0209	.02046	6.699
2._EDUCAG	.2682	.1963	1.047	.2682	.1963	1.046
3._EDUCAG	.3251	.2195	.7466	.3252	.2195	.7464
4._EDUCAG	.3483	.2271	.6368	.3484	.227	.6365
9._EDUCAG	.001717	.001715	24.07	.001718	.001715	24.06
2.EMPLOY1	.09302	.08439	2.802	.09303	.08438	2.802
3.EMPLOY1	.03749	.0361	4.869	.0375	.03609	4.869
4.EMPLOY1	.04264	.04084	4.527	.04265	.04083	4.527
5.EMPLOY1	.08901	.08111	2.887	.08902	.0811	2.886
6.EMPLOY1	.06268	.05877	3.608	.06269	.05876	3.608
7.EMPLOY1	.004865	.004843	14.23	.004866	.004843	14.23
8.EMPLOY1	.06068	.05701	3.68	.06068	.057	3.68
9.EMPLOY1	.008014	.007952	11.04	.008015	.007951	11.04
2._INCOMG	.16	.1344	1.855	.16	.1344	1.855
3._INCOMG	.09273	.08416	2.808	.09274	.08414	2.808
4._INCOMG	.1151	.1018	2.413	.1151	.1018	2.412
5._INCOMG	.4184	.2434	.3307	.4185	.2434	.3305
9._INCOMG	.1173	.1036	2.378	.1174	.1036	2.378
2._CHLDCNT	.1866	.1518	1.609	.1867	.1518	1.608
3._CHLDCNT	.2175	.1703	1.369	.2176	.1702	1.369
4._CHLDCNT	.1002	.09016	2.663	.1002	.09016	2.663
5._CHLDCNT	.05066	.04811	4.098	.05066	.0481	4.098
6._CHLDCNT	.03005	.02916	5.505	.03006	.02915	5.505
9._CHLDCNT	.006869	.006824	11.94	.00687	.006823	11.94
2.marital	.0996	.0897	2.674	.09961	.08969	2.674
3.marital	.006869	.006824	11.94	.00687	.006823	11.94
4.marital	.02719	.02646	5.814	.02719	.02646	5.814

5.marital	.3151	.2159	.796	.3152	.2159	.7957
6.marital	.06497	.06077	3.53	.06498	.06076	3.53
9.marital	.006869	.006824	11.94	.00687	.006823	11.94
1.menthlth1	.2272	.1757	1.302	.2273	.1756	1.301
2.menthlth1	.08958	.08158	2.874	.08959	.08157	2.874
3.menthlth1	.06211	.05827	3.629	.06211	.05826	3.628
4.menthlth1	.03463	.03344	5.09	.03463	.03344	5.09
5.menthlth1	.01231	.01216	8.847	.01231	.01216	8.846
6.menthlth1	.1093	.09741	2.504	.1094	.0974	2.503
77.menthlth1	.009731	.009639	9.989	.009732	.009638	9.988
99.menthlth1	.00229	.002285	20.83	.00229	.002285	20.83
1.physhlth1	.247	.186	1.173	.2471	.186	1.173
2.physhlth1	.06783	.06325	3.437	.06784	.06324	3.437
3.physhlth1	.03921	.03768	4.748	.03921	.03768	4.748
4.physhlth1	.01832	.01799	7.184	.01832	.01798	7.184
5.physhlth1	.01259	.01244	8.742	.01259	.01244	8.741
6.physhlth1	.06869	.06399	3.411	.0687	.06398	3.41
77.physhlth1	.0103	.0102	9.699	.01031	.0102	9.697
99.physhlth1	.002003	.002	22.27	.002004	.002	22.27
1.AlcCatb	.1895	.1536	1.585	.1895	.1536	1.584
2.AlcCatb	.08586	.07851	2.956	.08587	.0785	2.956
77.AlcCatb	.009445	.009358	10.14	.009446	.009357	10.14
99.AlcCatb	.001145	.001144	29.5	.001145	.001144	29.5
2._PAINDX1	.4284	.245	.2892	.4285	.2449	.289
9._PAINDX1	.03234	.0313	5.287	.03235	.0313	5.287
2._RFBMI5	.6208	.2355	-.4979	.6207	.2354	-.4976
9._RFBMI5	.06497	.06077	3.53	.06498	.06076	3.53

Table A11 Covariate distribution before and after entropy balancing (caregivers versus non-caregivers) – Analysis of tobacco use, age group 50-64

Treated units: 4459 total of weights: 4459
Control units: 13325 total of weights: 4459

Before: without weighting

	Caregivers (treat)			Non-caregivers (control)		
	mean	variance	skewness	mean	variance	skewness
_AGE80	57.47	17.87	-.1234	57.53	18.47	-.1554
2.sex	.6378	.2311	-.5735	.5299	.2491	-.1198
9.sex	.0004485	.0004484	47.19	.0003752	.0003751	51.59
2._RACEGR3	.05629	.05313	3.85	.05403	.05112	3.945
3._RACEGR3	.07625	.07045	3.193	.08293	.07606	3.025
4._RACEGR3	.05225	.04953	4.024	.03805	.0366	4.829
5._RACEGR3	.06885	.06412	3.406	.07947	.07316	3.109
9._RACEGR3	.01727	.01697	7.411	.02004	.01964	6.85
2._EDUCAG	.2312	.1778	1.275	.253	.189	1.136
3._EDUCAG	.3124	.2149	.8095	.2693	.1968	1.04
4._EDUCAG	.4149	.2428	.3455	.4155	.2429	.3431
9._EDUCAG	.002243	.002238	21.05	.003152	.003142	17.73
2.EMPLOY1	.1191	.1049	2.352	.1232	.1081	2.293
3.EMPLOY1	.03476	.03356	5.08	.03152	.03053	5.363
4.EMPLOY1	.02108	.02064	6.668	.02296	.02244	6.369
5.EMPLOY1	.05001	.04752	4.129	.03602	.03473	4.98
6.EMPLOY1	.002243	.002238	21.05	.002402	.002396	20.33
7.EMPLOY1	.1377	.1188	2.103	.137	.1183	2.111
8.EMPLOY1	.115	.1018	2.413	.1198	.1054	2.342
9.EMPLOY1	.005607	.005576	13.24	.007129	.007079	11.72
2._INCOMG	.1133	.1005	2.441	.1123	.09973	2.455
3._INCOMG	.0758	.07007	3.205	.06402	.05992	3.562
4._INCOMG	.1076	.09608	2.532	.09929	.08944	2.68
5._INCOMG	.5127	.2499	-.0507	.5153	.2498	-.06142
9._INCOMG	.1146	.1015	2.42	.1192	.105	2.351
2._CHLDCNT	.09935	.0895	2.679	.09936	.0895	2.679
3._CHLDCNT	.04642	.04428	4.312	.05156	.0489	4.056
4._CHLDCNT	.0157	.01546	7.792	.01644	.01617	7.607
5._CHLDCNT	.004934	.004911	14.13	.005478	.005449	13.4
6._CHLDCNT	.002243	.002238	21.05	.004053	.004036	15.61
9._CHLDCNT	.003813	.003799	16.1	.003977	.003962	15.76

2.marital	.1624	.136	1.831	.181	.1483	1.657
3.marital	.05203	.04933	4.034	.05666	.05345	3.835
4.marital	.02063	.02021	6.745	.02364	.02308	6.271
5.marital	.1081	.09643	2.524	.1167	.1031	2.388
6.marital	.02826	.02747	5.694	.02274	.02222	6.403
9.marital	.007176	.007127	11.68	.006079	.006042	12.71
1.menthlth1	.1902	.154	1.579	.1467	.1252	1.997
2.menthlth1	.05584	.05274	3.869	.04255	.04074	4.533
3.menthlth1	.04037	.03875	4.671	.03047	.02954	5.464
4.menthlth1	.02243	.02193	6.451	.01629	.01602	7.643
5.menthlth1	.009868	.009773	9.917	.005854	.00582	12.96
6.menthlth1	.08118	.07461	3.067	.05854	.05511	3.761
77.menthlth1	.008298	.008231	10.84	.01028	.01018	9.709
99.menthlth1	.00471	.004688	14.47	.005478	.005449	13.4
1.physhlth1	.2095	.1656	1.428	.1747	.1442	1.713
2.physhlth1	.06033	.0567	3.693	.04968	.04722	4.145
3.physhlth1	.03678	.03543	4.922	.03452	.03333	5.099
4.physhlth1	.02288	.02236	6.383	.01606	.0158	7.7
5.physhlth1	.01099	.01087	9.381	.009681	.009588	10.02
6.physhlth1	.09778	.08824	2.708	.09966	.08974	2.673
77.physhlth1	.009643	.009553	10.04	.01126	.01113	9.265
99.physhlth1	.002915	.002908	18.44	.004203	.004185	15.33
1.AlcCatb	.1651	.1378	1.804	.1537	.1301	1.92
2.AlcCatb	.04037	.03875	4.671	.04233	.04054	4.546
77.AlcCatb	.003813	.003799	16.1	.005854	.00582	12.96
99.AlcCatb	.00157	.001568	25.18	.001726	.001723	24.01
2._PAINDX1	.4216	.2439	.3174	.4581	.2483	.1682
9._PAINDX1	.03588	.0346	4.991	.03737	.03598	4.878
2._RFBMI5	.6667	.2222	-.7075	.6684	.2217	-.7152
9._RFBMI5	.06033	.0567	3.693	.05726	.05399	3.811

After: _uw1aTobaccoB2 as the weighting variable

	Caregivers (treat)			Non-caregivers (control)		
	mean	variance	skewness	mean	variance	skewness
_AGE80	57.47	17.87	-.1234	57.47	17.87	-.122
2.sex	.6378	.2311	-.5735	.6378	.231	-.5733
9.sex	.0004485	.0004484	47.19	.0004486	.0004484	47.18
2._RACEGR3	.05629	.05313	3.85	.0563	.05313	3.85
3._RACEGR3	.07625	.07045	3.193	.07626	.07045	3.193
4._RACEGR3	.05225	.04953	4.024	.05226	.04953	4.024
5._RACEGR3	.06885	.06412	3.406	.06885	.06412	3.405
9._RACEGR3	.01727	.01697	7.411	.01727	.01697	7.411
2._EDUCAG	.2312	.1778	1.275	.2313	.1778	1.275
3._EDUCAG	.3124	.2149	.8095	.3124	.2148	.8093
4._EDUCAG	.4149	.2428	.3455	.4149	.2428	.3454
9._EDUCAG	.002243	.002238	21.05	.002243	.002238	21.04
2.EMPLOY1	.1191	.1049	2.352	.1191	.1049	2.352
3.EMPLOY1	.03476	.03356	5.08	.03476	.03356	5.08
4.EMPLOY1	.02108	.02064	6.668	.02108	.02064	6.667
5.EMPLOY1	.05001	.04752	4.129	.05002	.04752	4.129
6.EMPLOY1	.002243	.002238	21.05	.002243	.002238	21.04
7.EMPLOY1	.1377	.1188	2.103	.1377	.1188	2.103
8.EMPLOY1	.115	.1018	2.413	.1151	.1018	2.413
9.EMPLOY1	.005607	.005576	13.24	.005607	.005576	13.24
2._INCOMG	.1133	.1005	2.441	.1133	.1005	2.44
3._INCOMG	.0758	.07007	3.205	.07581	.07007	3.205
4._INCOMG	.1076	.09608	2.532	.1077	.09608	2.532
5._INCOMG	.5127	.2499	-.0507	.5127	.2499	-.05068
9._INCOMG	.1146	.1015	2.42	.1146	.1015	2.419
2._CHLDCNT	.09935	.0895	2.679	.09936	.08949	2.679
3._CHLDCNT	.04642	.04428	4.312	.04643	.04427	4.311
4._CHLDCNT	.0157	.01546	7.792	.0157	.01545	7.792
5._CHLDCNT	.004934	.004911	14.13	.004934	.00491	14.13
6._CHLDCNT	.002243	.002238	21.05	.002243	.002238	21.04
9._CHLDCNT	.003813	.003799	16.1	.003813	.003799	16.1
2.marital	.1624	.136	1.831	.1624	.136	1.831
3.marital	.05203	.04933	4.034	.05203	.04933	4.034
4.marital	.02063	.02021	6.745	.02063	.02021	6.744
5.marital	.1081	.09643	2.524	.1081	.09643	2.524

6.marital	.02826	.02747	5.694	.02826	.02746	5.693
9.marital	.007176	.007127	11.68	.007177	.007126	11.68
1.menthlth1	.1902	.154	1.579	.1902	.154	1.579
2.menthlth1	.05584	.05274	3.869	.05585	.05273	3.869
3.menthlth1	.04037	.03875	4.671	.04037	.03874	4.67
4.menthlth1	.02243	.02193	6.451	.02243	.02193	6.451
5.menthlth1	.009868	.009773	9.917	.009869	.009772	9.917
6.menthlth1	.08118	.07461	3.067	.08119	.0746	3.067
77.menthlth1	.008298	.008231	10.84	.008299	.00823	10.84
99.menthlth1	.00471	.004688	14.47	.00471	.004688	14.47
1.physhlth1	.2095	.1656	1.428	.2095	.1656	1.428
2.physhlth1	.06033	.0567	3.693	.06033	.0567	3.693
3.physhlth1	.03678	.03543	4.922	.03678	.03543	4.922
4.physhlth1	.02288	.02236	6.383	.02288	.02236	6.382
5.physhlth1	.01099	.01087	9.381	.01099	.01087	9.381
6.physhlth1	.09778	.08824	2.708	.09779	.08823	2.708
77.physhlth1	.009643	.009553	10.04	.009644	.009552	10.03
99.physhlth1	.002915	.002908	18.44	.002916	.002907	18.44
1.AlcCatb	.1651	.1378	1.804	.1651	.1378	1.804
2.AlcCatb	.04037	.03875	4.671	.04037	.03874	4.67
77.AlcCatb	.003813	.003799	16.1	.003813	.003799	16.1
99.AlcCatb	.00157	.001568	25.18	.00157	.001568	25.18
2._PAINDX1	.4216	.2439	.3174	.4216	.2439	.3174
9._PAINDX1	.03588	.0346	4.991	.03589	.0346	4.99
2._RFBMI5	.6667	.2222	-.7075	.6667	.2222	-.7073
9._RFBMI5	.06033	.0567	3.693	.06033	.0567	3.693

Table A12 Covariate distribution before and after entropy balancing (caregivers versus non-caregivers) – Analysis of tobacco use, age group 65+

Treated units: 3980 total of weights: 3980
Control units: 16246 total of weights: 3980

Before: without weighting

		Caregivers (treat)			Non-caregivers (control)	
	mean	variance	skewness	mean	variance	skewness
_AGE80	72.15	25.86	.2612	73.04	27.68	.0221
2.sex	.6359	.2316	-.565	.5865	.2425	-.3512
9.sex	.0002513	.0002513	63.06	.0004924	.0004922	45.03
2._RACEGR3	.03869	.03721	4.784	.04038	.03875	4.67
3._RACEGR3	.0593	.05579	3.732	.07005	.06515	3.369
4._RACEGR3	.03693	.03558	4.911	.02918	.02833	5.595
5._RACEGR3	.04271	.0409	4.523	.04666	.04448	4.299
9._RACEGR3	.01834	.01801	7.179	.01982	.01943	6.89
2._EDUCAG	.2191	.1711	1.358	.2646	.1946	1.068
3._EDUCAG	.2962	.2085	.8926	.2665	.1955	1.056
4._EDUCAG	.4367	.2461	.2553	.4023	.2405	.3984
9._EDUCAG	.001759	.001756	23.78	.003385	.003374	17.1
2.EMPLOY1	.07136	.06628	3.33	.06285	.0589	3.603
3.EMPLOY1	.01407	.01388	8.251	.01077	.01066	9.479
4.EMPLOY1	.003518	.003506	16.77	.004801	.004778	14.33
5.EMPLOY1	.05101	.04842	4.082	.04561	.04353	4.356
6.EMPLOY1	.001256	.001255	28.16	.0006155	.0006152	40.27
7.EMPLOY1	.7121	.2051	-.9367	.7182	.2024	-.9701
8.EMPLOY1	.02739	.02664	5.792	.03946	.0379	4.731
9.EMPLOY1	.005025	.005001	14	.003939	.003924	15.84
2._INCOMG	.141	.1211	2.064	.1511	.1282	1.949
3._INCOMG	.109	.09718	2.509	.1054	.09428	2.57
4._INCOMG	.146	.1247	2.005	.1381	.119	2.098
5._INCOMG	.396	.2392	.4254	.3552	.2291	.605
9._INCOMG	.1633	.1367	1.822	.176	.145	1.702
2._CHLDCNT	.03065	.02972	5.446	.02296	.02243	6.37
3._CHLDCNT	.01206	.01192	8.94	.01016	.01005	9.771
4._CHLDCNT	.00402	.004005	15.68	.004186	.004168	15.36
5._CHLDCNT	.001759	.001756	23.78	.001724	.001721	24.03
6._CHLDCNT	.0002513	.0002513	63.06	.0004924	.0004922	45.03
9._CHLDCNT	.001759	.001756	23.78	.0008002	.0007996	35.31
2.marital	.1289	.1123	2.215	.1515	.1286	1.944

3.marital	.1844	.1504	1.627	.2924	.2069	.9129
4.marital	.01106	.01094	9.352	.01096	.01084	9.396
5.marital	.04749	.04524	4.255	.06439	.06024	3.55
6.marital	.01256	.01241	8.753	.009479	.00939	10.12
9.marital	.00402	.004005	15.68	.004432	.004412	14.92
1.menthlth1	.153	.1296	1.928	.1095	.09752	2.501
2.menthlth1	.04472	.04273	4.405	.03004	.02914	5.507
3.menthlth1	.02236	.02187	6.461	.0197	.01931	6.913
4.menthlth1	.01508	.01485	7.959	.008494	.008423	10.71
5.menthlth1	.00603	.005995	12.76	.003262	.003252	17.42
6.menthlth1	.0495	.04706	4.154	.03626	.03494	4.962
77.menthlth1	.01206	.01192	8.94	.01188	.01174	9.01
99.menthlth1	.00402	.004005	15.68	.005171	.005144	13.8
1.physhlth1	.1867	.1519	1.608	.1525	.1293	1.933
2.physhlth1	.0598	.05624	3.713	.05257	.04981	4.01
3.physhlth1	.03367	.03254	5.171	.03656	.03523	4.938
4.physhlth1	.01457	.01436	8.102	.01502	.01479	7.975
5.physhlth1	.01005	.009952	9.824	.008679	.008604	10.59
6.physhlth1	.08719	.0796	2.927	.09805	.08845	2.703
77.physhlth1	.01658	.01631	7.571	.02314	.02261	6.343
99.physhlth1	.004271	.004254	15.2	.006771	.006725	12.03
1.AlcCatb	.08693	.0794	2.932	.09276	.08416	2.808
2.AlcCatb	.01633	.01607	7.632	.0168	.01652	7.518
77.AlcCatb	.003015	.003007	18.13	.005417	.005388	13.48
99.AlcCatb	.001508	.001506	25.7	.001847	.001843	23.21
2._PAINDX1	.403	.2407	.3955	.4288	.2449	.2878
9._PAINDX1	.03015	.02925	5.495	.03749	.03608	4.87
2._RFBMI5	.6467	.2285	-.614	.6114	.2376	-.4569
9._RFBMI5	.04497	.04296	4.391	.0477	.04543	4.244

After: _uw1aTobaccoB3 as the weighting variable

	Caregivers (treat)			Non-caregivers (control)		
	mean	variance	skewness	mean	variance	skewness
_AGE80	72.15	25.86	.2612	72.15	25.86	.2631
2.sex	.6359	.2316	-.565	.6359	.2316	-.5648
9.sex	.0002513	.0002513	63.06	.0002514	.0002513	63.05
2._RACEGR3	.03869	.03721	4.784	.0387	.0372	4.783
3._RACEGR3	.0593	.05579	3.732	.0593	.05579	3.732
4._RACEGR3	.03693	.03558	4.911	.03694	.03558	4.91
5._RACEGR3	.04271	.0409	4.523	.04272	.0409	4.523
9._RACEGR3	.01834	.01801	7.179	.01834	.01801	7.179
2._EDUCAG	.2191	.1711	1.358	.2191	.1711	1.358
3._EDUCAG	.2962	.2085	.8926	.2963	.2085	.8923
4._EDUCAG	.4367	.2461	.2553	.4367	.246	.2553
9._EDUCAG	.001759	.001756	23.78	.00176	.001757	23.78
2.EMPLOY1	.07136	.06628	3.33	.07136	.06627	3.33
3.EMPLOY1	.01407	.01388	8.251	.01407	.01387	8.251
4.EMPLOY1	.003518	.003506	16.77	.003518	.003506	16.77
5.EMPLOY1	.05101	.04842	4.082	.05101	.04841	4.081
6.EMPLOY1	.001256	.001255	28.16	.001256	.001255	28.16
7.EMPLOY1	.7121	.2051	-.9367	.712	.2051	-.9364
8.EMPLOY1	.02739	.02664	5.792	.02739	.02664	5.791
9.EMPLOY1	.005025	.005001	14	.005026	.005001	14
2._INCOMG	.141	.1211	2.064	.141	.1211	2.063
3._INCOMG	.109	.09718	2.509	.1091	.09718	2.508
4._INCOMG	.146	.1247	2.005	.146	.1247	2.005
5._INCOMG	.396	.2392	.4254	.396	.2392	.4253
9._INCOMG	.1633	.1367	1.822	.1634	.1367	1.821
2._CHLDCNT	.03065	.02972	5.446	.03066	.02972	5.445
3._CHLDCNT	.01206	.01192	8.94	.01206	.01192	8.94
4._CHLDCNT	.00402	.004005	15.68	.004021	.004005	15.68
5._CHLDCNT	.001759	.001756	23.78	.001759	.001756	23.78
6._CHLDCNT	.0002513	.0002513	63.06	.0002513	.0002513	63.05
9._CHLDCNT	.001759	.001756	23.78	.001759	.001756	23.78
2.marital	.1289	.1123	2.215	.1289	.1123	2.215
3.marital	.1844	.1504	1.627	.1845	.1505	1.627
4.marital	.01106	.01094	9.352	.01106	.01093	9.352
5.marital	.04749	.04524	4.255	.04749	.04524	4.255
6.marital	.01256	.01241	8.753	.01256	.01241	8.752

9.marital	.00402	.004005	15.68	.004021	.004005	15.68
1.menthlth1	.153	.1296	1.928	.153	.1296	1.927
2.menthlth1	.04472	.04273	4.405	.04473	.04273	4.405
3.menthlth1	.02236	.02187	6.461	.02236	.02187	6.46
4.menthlth1	.01508	.01485	7.959	.01508	.01485	7.959
5.menthlth1	.00603	.005995	12.76	.006031	.005995	12.76
6.menthlth1	.0495	.04706	4.154	.0495	.04705	4.154
77.menthlth1	.01206	.01192	8.94	.01206	.01192	8.94
99.menthlth1	.00402	.004005	15.68	.004021	.004005	15.68
1.physhlth1	.1867	.1519	1.608	.1867	.1519	1.608
2.physhlth1	.0598	.05624	3.713	.0598	.05623	3.713
3.physhlth1	.03367	.03254	5.171	.03367	.03254	5.17
4.physhlth1	.01457	.01436	8.102	.01457	.01436	8.101
5.physhlth1	.01005	.009952	9.824	.01005	.009952	9.823
6.physhlth1	.08719	.0796	2.927	.0872	.0796	2.926
77.physhlth1	.01658	.01631	7.571	.01659	.01631	7.57
99.physhlth1	.004271	.004254	15.2	.004272	.004254	15.2
1.AlcCatb	.08693	.0794	2.932	.08694	.07939	2.932
2.AlcCatb	.01633	.01607	7.632	.01633	.01607	7.632
77.AlcCatb	.003015	.003007	18.13	.003016	.003007	18.13
99.AlcCatb	.001508	.001506	25.7	.001508	.001506	25.7
2._PAINDX1	.403	.2407	.3955	.4031	.2406	.3953
9._PAINDX1	.03015	.02925	5.495	.03015	.02925	5.495
2._RFBMI5	.6467	.2285	-.614	.6467	.2285	-.6137
9._RFBMI5	.04497	.04296	4.391	.04498	.04296	4.391

Table A13 Covariate distribution before and after entropy balancing (caregivers versus non-caregivers) – Analysis of physical activity, all age groups

Treated units: 11933 total of weights: 11933
Control units: 45416 total of weights: 11933

Before: without weighting

		Caregivers (treat)			Non-caregivers (control)	
	mean	variance	skewness	mean	variance	skewness
2.sex	.6295	.2332	-.5364	.539	.2485	-.1564
9.sex	.0005028	.0005026	44.56	.0004404	.0004402	47.62
_AGE80	56.15	241	-.5299	55.19	302.3	-.3922
2._RACEGR3	.05447	.05151	3.926	.05082	.04824	4.09
3._RACEGR3	.07969	.07335	3.104	.08865	.08079	2.894
4._RACEGR3	.0507	.04813	4.096	.04375	.04184	4.461
5._RACEGR3	.08238	.0756	3.038	.09261	.08404	2.811
9._RACEGR3	.01869	.01834	7.108	.01803	.01771	7.244
2._EDUCAG	.238	.1814	1.23	.2569	.1909	1.113
3._EDUCAG	.3107	.2142	.8179	.2715	.1978	1.028
4._EDUCAG	.4027	.2405	.3969	.4067	.2413	.3798
9._EDUCAG	.001927	.001924	22.71	.003281	.00327	17.37
2.EMPLOY1	.09553	.08641	2.752	.08944	.08144	2.877
3.EMPLOY1	.02866	.02784	5.65	.02129	.02084	6.632
4.EMPLOY1	.02154	.02107	6.592	.02072	.02029	6.729
5.EMPLOY1	.06176	.05795	3.641	.04853	.04618	4.202
6.EMPLOY1	.01961	.01923	6.929	.02728	.02654	5.804
7.EMPLOY1	.2904	.2061	.9236	.2989	.2095	.8788
8.EMPLOY1	.06989	.06501	3.374	.0637	.05964	3.573
9.EMPLOY1	.006117	.006081	12.67	.006914	.006866	11.9
2._INCOMG	.1362	.1176	2.122	.1316	.1143	2.18
3._INCOMG	.09185	.08342	2.826	.08327	.07634	3.017
4._INCOMG	.1226	.1076	2.301	.1179	.104	2.37
5._INCOMG	.4462	.2471	.2166	.4491	.2474	.2047
9._INCOMG	.1317	.1143	2.179	.1403	.1206	2.071
2._CHLDCNT	.102	.09159	2.63	.1067	.09528	2.549
3._CHLDCNT	.08506	.07783	2.975	.09197	.08351	2.824
4._CHLDCNT	.03654	.03521	4.94	.04029	.03867	4.675
5._CHLDCNT	.01726	.01697	7.412	.01603	.01577	7.707
6._CHLDCNT	.009721	.009627	9.994	.008852	.008773	10.49
9._CHLDCNT	.004022	.004007	15.67	.004492	.004472	14.82
2.marital	.1328	.1152	2.164	.137	.1182	2.111
3.marital	.08296	.07609	3.024	.1237	.1084	2.286
4.marital	.01936	.01898	6.977	.01955	.01917	6.94

5.marital	.1485	.1265	1.977	.1707	.1416	1.751
6.marital	.03377	.03263	5.162	.03195	.03093	5.323
9.marital	.006034	.005998	12.76	.005086	.005061	13.91
1.menthlth1	.1886	.1531	1.592	.1559	.1316	1.897
2.menthlth1	.06201	.05817	3.632	.04562	.04354	4.355
3.menthlth1	.04073	.03907	4.647	.03149	.0305	5.366
4.menthlth1	.02355	.023	6.284	.01451	.0143	8.12
5.menthlth1	.009302	.009216	10.22	.005791	.005758	13.03
6.menthlth1	.07886	.07264	3.125	.04928	.04685	4.165
77.menthlth1	.009972	.009874	9.863	.009996	.009897	9.851
99.menthlth1	.003771	.003757	16.19	.004338	.004319	15.08
1.physhlth1	.2129	.1676	1.403	.1799	.1476	1.666
2.physhlth1	.06235	.05847	3.62	.04915	.04673	4.171
3.physhlth1	.03645	.03513	4.947	.03142	.03043	5.372
4.physhlth1	.01877	.01842	7.092	.01339	.01321	8.468
5.physhlth1	.01115	.01102	9.313	.007905	.007842	11.11
6.physhlth1	.08573	.07839	2.959	.07878	.07258	3.127
77.physhlth1	.01215	.012	8.906	.01462	.01441	8.088
99.physhlth1	.003101	.003091	17.88	.004624	.004603	14.6
1.AlcCatb	.1461	.1248	2.003	.1483	.1263	1.979
2.AlcCatb	.04567	.04359	4.352	.04943	.04699	4.157
77.AlcCatb	.005196	.005169	13.76	.00665	.006606	12.14
99.AlcCatb	.001425	.001423	26.44	.001585	.001583	25.06
2._RFSMOK3	.1602	.1346	1.853	.1246	.1091	2.274
9._RFSMOK3	.005112	.005086	13.88	.005791	.005758	13.03

After: _uw1aPA as the weighting variable

	Caregivers (treat)			Non-caregivers (control)		
	mean	variance	skewness	mean	variance	skewness
2.sex	.6295	.2332	-.5364	.6295	.2332	-.5363
9.sex	.0005028	.0005026	44.56	.0005028	.0005026	44.56
_AGE80	56.15	241	-.5299	56.15	241	-.5298
2._RACEGR3	.05447	.05151	3.926	.05447	.05151	3.926
3._RACEGR3	.07969	.07335	3.104	.0797	.07335	3.104
4._RACEGR3	.0507	.04813	4.096	.0507	.04813	4.096
5._RACEGR3	.08238	.0756	3.038	.08238	.0756	3.038
9._RACEGR3	.01869	.01834	7.108	.01869	.01834	7.108
2._EDUCAG	.238	.1814	1.23	.238	.1814	1.23
3._EDUCAG	.3107	.2142	.8179	.3108	.2142	.8178
4._EDUCAG	.4027	.2405	.3969	.4027	.2405	.3969
9._EDUCAG	.001927	.001924	22.71	.001928	.001924	22.71
2.EMPLOY1	.09553	.08641	2.752	.09554	.08641	2.752
3.EMPLOY1	.02866	.02784	5.65	.02866	.02784	5.65
4.EMPLOY1	.02154	.02107	6.592	.02154	.02107	6.592
5.EMPLOY1	.06176	.05795	3.641	.06176	.05795	3.641
6.EMPLOY1	.01961	.01923	6.929	.01961	.01923	6.929
7.EMPLOY1	.2904	.2061	.9236	.2904	.2061	.9235
8.EMPLOY1	.06989	.06501	3.374	.06989	.06501	3.374
9.EMPLOY1	.006117	.006081	12.67	.006118	.006081	12.67
2._INCOMG	.1362	.1176	2.122	.1362	.1176	2.121
3._INCOMG	.09185	.08342	2.826	.09185	.08341	2.826
4._INCOMG	.1226	.1076	2.301	.1226	.1076	2.301
5._INCOMG	.4462	.2471	.2166	.4462	.2471	.2166
9._INCOMG	.1317	.1143	2.179	.1317	.1143	2.179
2._CHLDCNT	.102	.09159	2.63	.102	.09159	2.63
3._CHLDCNT	.08506	.07783	2.975	.08506	.07783	2.975
4._CHLDCNT	.03654	.03521	4.94	.03654	.0352	4.94
5._CHLDCNT	.01726	.01697	7.412	.01726	.01697	7.412
6._CHLDCNT	.009721	.009627	9.994	.009721	.009627	9.994
9._CHLDCNT	.004022	.004007	15.67	.004023	.004007	15.67
2.marital	.1328	.1152	2.164	.1328	.1152	2.164
3.marital	.08296	.07609	3.024	.08297	.07609	3.024
4.marital	.01936	.01898	6.977	.01936	.01898	6.977
5.marital	.1485	.1265	1.977	.1485	.1265	1.977
6.marital	.03377	.03263	5.162	.03377	.03263	5.162
9.marital	.006034	.005998	12.76	.006034	.005998	12.76
1.menthlth1	.1886	.1531	1.592	.1886	.1531	1.592
2.menthlth1	.06201	.05817	3.632	.06201	.05817	3.632
3.menthlth1	.04073	.03907	4.647	.04073	.03907	4.647

4.menthlth1	.02355	.023	6.284	.02355	.02299	6.284
5.menthlth1	.009302	.009216	10.22	.009302	.009216	10.22
6.menthlth1	.07886	.07264	3.125	.07886	.07264	3.125
77.menthlth1	.009972	.009874	9.863	.009973	.009874	9.863
99.menthlth1	.003771	.003757	16.19	.003771	.003757	16.19
1.physhlth1	.2129	.1676	1.403	.2129	.1676	1.403
2.physhlth1	.06235	.05847	3.62	.06235	.05846	3.62
3.physhlth1	.03645	.03513	4.947	.03645	.03513	4.947
4.physhlth1	.01877	.01842	7.092	.01877	.01842	7.092
5.physhlth1	.01115	.01102	9.313	.01115	.01102	9.313
6.physhlth1	.08573	.07839	2.959	.08573	.07838	2.959
77.physhlth1	.01215	.012	8.906	.01215	.012	8.905
99.physhlth1	.003101	.003091	17.88	.003101	.003091	17.87
1.AlcCatb	.1461	.1248	2.003	.1462	.1248	2.003
2.AlcCatb	.04567	.04359	4.352	.04567	.04359	4.352
77.AlcCatb	.005196	.005169	13.76	.005196	.005169	13.76
99.AlcCatb	.001425	.001423	26.44	.001425	.001423	26.44
2._RFSMOK3	.1602	.1346	1.853	.1602	.1346	1.852
9._RFSMOK3	.005112	.005086	13.88	.005112	.005086	13.88

Table A14 Covariate distribution before and after entropy balancing (caregivers versus non-caregivers) – Analysis of physical activity, age group 18-49

Treated units: 3494 total of weights: 3494
Control units: 15845 total of weights: 3494

Before: without weighting

	Caregivers (treat)			Non-caregivers (control)		
	mean	variance	skewness	mean	variance	skewness
_AGE80	36.25	80.75	-.3605	34.94	81.16	-.167
2.sex	.6116	.2376	-.458	.4979	.25	.008457
9.sex	.0008586	.0008581	34.08	.0004418	.0004416	47.55
2._RACEGR3	.07012	.06522	3.367	.05882	.05536	3.75
3._RACEGR3	.1073	.09584	2.537	.1125	.09987	2.452
4._RACEGR3	.0644	.06027	3.549	.06349	.05946	3.58
5._RACEGR3	.1448	.1239	2.019	.1508	.128	1.952
9._RACEGR3	.02089	.02046	6.7	.01452	.01431	8.118
2._EDUCAG	.2682	.1963	1.047	.2524	.1887	1.14
3._EDUCAG	.3251	.2195	.7466	.2785	.201	.9882
4._EDUCAG	.3483	.2271	.6368	.4038	.2408	.3919
9._EDUCAG	.001717	.001715	24.07	.003282	.003271	17.37
2.EMPLOY1	.09302	.08439	2.802	.08829	.0805	2.902
3.EMPLOY1	.03749	.0361	4.869	.02348	.02293	6.294
4.EMPLOY1	.04264	.04084	4.527	.03515	.03392	5.048
5.EMPLOY1	.08901	.08111	2.887	.06204	.05819	3.631
6.EMPLOY1	.06268	.05877	3.608	.07554	.06984	3.212
7.EMPLOY1	.004865	.004843	14.23	.004986	.004961	14.06
8.EMPLOY1	.06068	.05701	3.68	.0414	.03969	4.604
9.EMPLOY1	.008014	.007952	11.04	.009782	.009687	9.962
2._INCOMG	.16	.1344	1.855	.1278	.1115	2.23
3._INCOMG	.09273	.08416	2.808	.07681	.07091	3.179
4._INCOMG	.1151	.1018	2.413	.1128	.1001	2.448
5._INCOMG	.4184	.2434	.3307	.4896	.2499	.04154
9._INCOMG	.1173	.1036	2.378	.1215	.1067	2.317
2._CHLDCNT	.1866	.1518	1.609	.1986	.1592	1.511
3._CHLDCNT	.2175	.1703	1.369	.2098	.1658	1.425
4._CHLDCNT	.1002	.09016	2.663	.09738	.0879	2.716
5._CHLDCNT	.05066	.04811	4.098	.03957	.03801	4.724
6._CHLDCNT	.03005	.02916	5.505	.02146	.021	6.605
9._CHLDCNT	.006869	.006824	11.94	.008709	.008634	10.57
2.marital	.0996	.0897	2.674	.08514	.07789	2.973
3.marital	.006869	.006824	11.94	.007132	.007081	11.71
4.marital	.02719	.02646	5.814	.02493	.02431	6.094
5.marital	.3151	.2159	.796	.3251	.2194	.7468
6.marital	.06497	.06077	3.53	.06273	.0588	3.607
9.marital	.006869	.006824	11.94	.004923	.004899	14.15
1.menthlth1	.2272	.1757	1.302	.2112	.1666	1.415
2.menthlth1	.08958	.08158	2.874	.06418	.06007	3.557
3.menthlth1	.06211	.05827	3.629	.04443	.04246	4.422

4.menthlth1	.03463	.03344	5.09	.01919	.01882	7.01
5.menthlth1	.01231	.01216	8.847	.008331	.008262	10.82
6.menthlth1	.1093	.09741	2.504	.05484	.05184	3.91
77.menthlth1	.009731	.009639	9.989	.007826	.007765	11.17
99.menthlth1	.00229	.002285	20.83	.002524	.002518	19.83
1.physhlth1	.247	.186	1.173	.2124	.1673	1.406
2.physhlth1	.06783	.06325	3.437	.04519	.04315	4.379
3.physhlth1	.03921	.03768	4.748	.02354	.02299	6.285
4.physhlth1	.01832	.01799	7.184	.009467	.009378	10.13
5.physhlth1	.01259	.01244	8.742	.005617	.005586	13.23
6.physhlth1	.06869	.06399	3.411	.04146	.03975	4.6
77.physhlth1	.0103	.0102	9.699	.008709	.008634	10.57
99.physhlth1	.002003	.002	22.27	.002777	.002769	18.9
1.AlcCatb	.1895	.1536	1.585	.2008	.1605	1.494
2.AlcCatb	.08586	.07851	2.956	.08886	.08097	2.89
77.AlcCatb	.009445	.009358	10.14	.008583	.00851	10.65
99.AlcCatb	.001145	.001144	29.5	.001199	.001198	28.83
2._RFSMOK3	.2327	.1786	1.265	.1529	.1295	1.929
9._RFSMOK3	.005438	.00541	13.45	.004481	.004461	14.84

After: _uw1aPAB1 as the weighting variable

	Caregivers (treat)			Non-caregivers (control)		
	mean	variance	skewness	mean	variance	skewness
_AGE80	36.25	80.75	-.3605	36.25	80.75	-.3601
2.sex	.6116	.2376	-.458	.6116	.2376	-.4578
9.sex	.0008586	.0008581	34.08	.0008587	.000858	34.08
2._RACEGR3	.07012	.06522	3.367	.07013	.06522	3.367
3._RACEGR3	.1073	.09584	2.537	.1074	.09583	2.537
4._RACEGR3	.0644	.06027	3.549	.0644	.06026	3.549
5._RACEGR3	.1448	.1239	2.019	.1449	.1239	2.018
9._RACEGR3	.02089	.02046	6.7	.0209	.02046	6.699
2._EDUCAG	.2682	.1963	1.047	.2682	.1963	1.046
3._EDUCAG	.3251	.2195	.7466	.3252	.2195	.7463
4._EDUCAG	.3483	.2271	.6368	.3484	.227	.6365
9._EDUCAG	.001717	.001715	24.07	.001718	.001715	24.06
2.EMPLOY1	.09302	.08439	2.802	.09303	.08438	2.802
3.EMPLOY1	.03749	.0361	4.869	.0375	.03609	4.869
4.EMPLOY1	.04264	.04084	4.527	.04265	.04083	4.527
5.EMPLOY1	.08901	.08111	2.887	.08902	.0811	2.886
6.EMPLOY1	.06268	.05877	3.608	.06269	.05876	3.608
7.EMPLOY1	.004865	.004843	14.23	.004866	.004843	14.23
8.EMPLOY1	.06068	.05701	3.68	.06068	.057	3.68
9.EMPLOY1	.008014	.007952	11.04	.008015	.007951	11.04
2._INCOMG	.16	.1344	1.855	.16	.1344	1.855
3._INCOMG	.09273	.08416	2.808	.09274	.08415	2.808
4._INCOMG	.1151	.1018	2.413	.1151	.1018	2.412
5._INCOMG	.4184	.2434	.3307	.4185	.2434	.3305
9._INCOMG	.1173	.1036	2.378	.1174	.1036	2.377
2._CHLDCNT	.1866	.1518	1.609	.1867	.1518	1.608
3._CHLDCNT	.2175	.1703	1.369	.2176	.1702	1.369
4._CHLDCNT	.1002	.09016	2.663	.1002	.09016	2.663
5._CHLDCNT	.05066	.04811	4.098	.05066	.0481	4.098
6._CHLDCNT	.03005	.02916	5.505	.03006	.02915	5.505
9._CHLDCNT	.006869	.006824	11.94	.00687	.006823	11.94
2.marital	.0996	.0897	2.674	.09961	.08969	2.674
3.marital	.006869	.006824	11.94	.00687	.006823	11.94
4.marital	.02719	.02646	5.814	.02719	.02646	5.814
5.marital	.3151	.2159	.796	.3152	.2159	.7957
6.marital	.06497	.06077	3.53	.06498	.06076	3.53
9.marital	.006869	.006824	11.94	.00687	.006823	11.94
1.menthlth1	.2272	.1757	1.302	.2273	.1756	1.301
2.menthlth1	.08958	.08158	2.874	.08959	.08157	2.874
3.menthlth1	.06211	.05827	3.629	.06211	.05826	3.628
4.menthlth1	.03463	.03344	5.09	.03464	.03344	5.09
5.menthlth1	.01231	.01216	8.847	.01231	.01216	8.846
6.menthlth1	.1093	.09741	2.504	.1094	.0974	2.503
77.menthlth1	.009731	.009639	9.989	.009732	.009638	9.988
99.menthlth1	.00229	.002285	20.83	.00229	.002285	20.82
1.physhlth1	.247	.186	1.173	.2471	.186	1.173

2.physhlth1	.06783	.06325	3.437	.06784	.06324	3.437
3.physhlth1	.03921	.03768	4.748	.03922	.03768	4.748
4.physhlth1	.01832	.01799	7.184	.01832	.01799	7.184
5.physhlth1	.01259	.01244	8.742	.01259	.01244	8.741
6.physhlth1	.06869	.06399	3.411	.0687	.06398	3.41
77.physhlth1	.0103	.0102	9.699	.01031	.0102	9.697
99.physhlth1	.002003	.002	22.27	.002004	.002	22.27
1.AlcCatb	.1895	.1536	1.585	.1895	.1536	1.584
2.AlcCatb	.08586	.07851	2.956	.08587	.0785	2.956
77.AlcCatb	.009445	.009358	10.14	.009446	.009358	10.14
99.AlcCatb	.001145	.001144	29.5	.001145	.001144	29.5
2._RFSMOK3	.2327	.1786	1.265	.2327	.1786	1.265
9._RFSMOK3	.005438	.00541	13.45	.005439	.005409	13.45

Table A15 Covariate distribution before and after entropy balancing (caregivers versus non-caregivers) – Analysis of physical activity, age group 50-64

Treated units: 4459 total of weights: 4459
Control units: 13325 total of weights: 4459

Before: without weighting

	Caregivers (treat)			Non-caregivers (control)		
	mean	variance	skewness	mean	variance	skewness
_AGE80	57.47	17.87	-.1234	57.53	18.47	-.1554
2.sex	.6378	.2311	-.5735	.5299	.2491	-.1198
9.sex	.0004485	.0004484	47.19	.0003752	.0003751	51.59
2._RACEGR3	.05629	.05313	3.85	.05403	.05112	3.945
3._RACEGR3	.07625	.07045	3.193	.08293	.07606	3.025
4._RACEGR3	.05225	.04953	4.024	.03805	.0366	4.829
5._RACEGR3	.06885	.06412	3.406	.07947	.07316	3.109
9._RACEGR3	.01727	.01697	7.411	.02004	.01964	6.85
2._EDUCAG	.2312	.1778	1.275	.253	.189	1.136
3._EDUCAG	.3124	.2149	.8095	.2693	.1968	1.04
4._EDUCAG	.4149	.2428	.3455	.4155	.2429	.3431
9._EDUCAG	.002243	.002238	21.05	.003152	.003142	17.73
2.EMPLOY1	.1191	.1049	2.352	.1232	.1081	2.293
3.EMPLOY1	.03476	.03356	5.08	.03152	.03053	5.363
4.EMPLOY1	.02108	.02064	6.668	.02296	.02244	6.369
5.EMPLOY1	.05001	.04752	4.129	.03602	.03473	4.98
6.EMPLOY1	.002243	.002238	21.05	.002402	.002396	20.33
7.EMPLOY1	.1377	.1188	2.103	.137	.1183	2.111
8.EMPLOY1	.115	.1018	2.413	.1198	.1054	2.342
9.EMPLOY1	.005607	.005576	13.24	.007129	.007079	11.72
2._INCOMG	.1133	.1005	2.441	.1123	.09973	2.455
3._INCOMG	.0758	.07007	3.205	.06402	.05992	3.562
4._INCOMG	.1076	.09608	2.532	.09929	.08944	2.68
5._INCOMG	.5127	.2499	-.0507	.5153	.2498	-.06142
9._INCOMG	.1146	.1015	2.42	.1192	.105	2.351
2._CHLDCNT	.09935	.0895	2.679	.09936	.0895	2.679
3._CHLDCNT	.04642	.04428	4.312	.05156	.0489	4.056
4._CHLDCNT	.0157	.01546	7.792	.01644	.01617	7.607
5._CHLDCNT	.004934	.004911	14.13	.005478	.005449	13.4
6._CHLDCNT	.002243	.002238	21.05	.004053	.004036	15.61
9._CHLDCNT	.003813	.003799	16.1	.003977	.003962	15.76
2.marital	.1624	.136	1.831	.181	.1483	1.657
3.marital	.05203	.04933	4.034	.05666	.05345	3.835
4.marital	.02063	.02021	6.745	.02364	.02308	6.271
5.marital	.1081	.09643	2.524	.1167	.1031	2.388
6.marital	.02826	.02747	5.694	.02274	.02222	6.403
9.marital	.007176	.007127	11.68	.006079	.006042	12.71
1.menthlth1	.1902	.154	1.579	.1467	.1252	1.997
2.menthlth1	.05584	.05274	3.869	.04255	.04074	4.533
3.menthlth1	.04037	.03875	4.671	.03047	.02954	5.464
4.menthlth1	.02243	.02193	6.451	.01629	.01602	7.643
5.menthlth1	.009868	.009773	9.917	.005854	.00582	12.96
6.menthlth1	.08118	.07461	3.067	.05854	.05511	3.761
77.menthlth1	.008298	.008231	10.84	.01028	.01018	9.709
99.menthlth1	.00471	.004688	14.47	.005478	.005449	13.4
1.physhlth1	.2095	.1656	1.428	.1747	.1442	1.713

2.physhlth1	.06033	.0567	3.693	.04968	.04722	4.145
3.physhlth1	.03678	.03543	4.922	.03452	.03333	5.099
4.physhlth1	.02288	.02236	6.383	.01606	.0158	7.7
5.physhlth1	.01099	.01087	9.381	.009681	.009588	10.02
6.physhlth1	.09778	.08824	2.708	.09966	.08974	2.673
77.physhlth1	.009643	.009553	10.04	.01126	.01113	9.265
99.physhlth1	.002915	.002908	18.44	.004203	.004185	15.33
1.AlcCatb	.1651	.1378	1.804	.1537	.1301	1.92
2.AlcCatb	.04037	.03875	4.671	.04233	.04054	4.546
77.AlcCatb	.003813	.003799	16.1	.005854	.00582	12.96
99.AlcCatb	.00157	.001568	25.18	.001726	.001723	24.01
2._RFSMOK3	.1743	.1439	1.717	.1484	.1264	1.978
9._RFSMOK3	.004037	.004021	15.64	.004803	.00478	14.33

After: _uw1aPAB2 as the weighting variable

	Caregivers (treat)			Non-caregivers (control)		
	mean	variance	skewness	mean	variance	skewness
_AGE80	57.47	17.87	-.1234	57.47	17.87	-.1223
2.sex	.6378	.2311	-.5735	.6378	.231	-.5733
9.sex	.0004485	.0004484	47.19	.0004486	.0004484	47.18
2._RACEGR3	.05629	.05313	3.85	.0563	.05313	3.85
3._RACEGR3	.07625	.07045	3.193	.07626	.07045	3.193
4._RACEGR3	.05225	.04953	4.024	.05226	.04953	4.024
5._RACEGR3	.06885	.06412	3.406	.06886	.06412	3.405
9._RACEGR3	.01727	.01697	7.411	.01727	.01697	7.411
2._EDUCAG	.2312	.1778	1.275	.2313	.1778	1.275
3._EDUCAG	.3124	.2149	.8095	.3124	.2148	.8093
4._EDUCAG	.4149	.2428	.3455	.4149	.2428	.3454
9._EDUCAG	.002243	.002238	21.05	.002243	.002238	21.04
2.EMPLOY1	.1191	.1049	2.352	.1191	.1049	2.352
3.EMPLOY1	.03476	.03356	5.08	.03476	.03356	5.079
4.EMPLOY1	.02108	.02064	6.668	.02108	.02064	6.667
5.EMPLOY1	.05001	.04752	4.129	.05002	.04752	4.129
6.EMPLOY1	.002243	.002238	21.05	.002243	.002238	21.04
7.EMPLOY1	.1377	.1188	2.103	.1377	.1188	2.103
8.EMPLOY1	.115	.1018	2.413	.1151	.1018	2.413
9.EMPLOY1	.005607	.005576	13.24	.005607	.005576	13.24
2._INCOMG	.1133	.1005	2.441	.1133	.1005	2.44
3._INCOMG	.0758	.07007	3.205	.07581	.07007	3.205
4._INCOMG	.1076	.09608	2.532	.1077	.09608	2.532
5._INCOMG	.5127	.2499	-.0507	.5127	.2499	-.05068
9._INCOMG	.1146	.1015	2.42	.1146	.1015	2.419
2._CHLDCNT	.09935	.0895	2.679	.09936	.08949	2.679
3._CHLDCNT	.04642	.04428	4.312	.04643	.04427	4.311
4._CHLDCNT	.0157	.01546	7.792	.0157	.01545	7.792
5._CHLDCNT	.004934	.004911	14.13	.004934	.00491	14.13
6._CHLDCNT	.002243	.002238	21.05	.002243	.002238	21.04
9._CHLDCNT	.003813	.003799	16.1	.003813	.003799	16.1
2.marital	.1624	.136	1.831	.1624	.136	1.831
3.marital	.05203	.04933	4.034	.05203	.04933	4.034
4.marital	.02063	.02021	6.745	.02063	.02021	6.744
5.marital	.1081	.09643	2.524	.1081	.09643	2.524
6.marital	.02826	.02747	5.694	.02826	.02746	5.693
9.marital	.007176	.007127	11.68	.007177	.007126	11.68
1.menthlth1	.1902	.154	1.579	.1902	.154	1.579
2.menthlth1	.05584	.05274	3.869	.05585	.05273	3.868
3.menthlth1	.04037	.03875	4.671	.04037	.03874	4.67
4.menthlth1	.02243	.02193	6.451	.02243	.02193	6.45
5.menthlth1	.009868	.009773	9.917	.009869	.009772	9.917
6.menthlth1	.08118	.07461	3.067	.08119	.0746	3.067
77.menthlth1	.008298	.008231	10.84	.008299	.00823	10.84
99.menthlth1	.00471	.004688	14.47	.00471	.004688	14.47
1.physhlth1	.2095	.1656	1.428	.2095	.1656	1.428
2.physhlth1	.06033	.0567	3.693	.06033	.0567	3.693
3.physhlth1	.03678	.03543	4.922	.03678	.03543	4.922
4.physhlth1	.02288	.02236	6.383	.02288	.02236	6.382
5.physhlth1	.01099	.01087	9.381	.01099	.01087	9.381
6.physhlth1	.09778	.08824	2.708	.09779	.08823	2.708
77.physhlth1	.009643	.009553	10.04	.009644	.009552	10.03

99.physhlth1	.002915	.002908	18.44	.002916	.002907	18.44
1.AlcCatb	.1651	.1378	1.804	.1651	.1378	1.804
2.AlcCatb	.04037	.03875	4.671	.04037	.03874	4.67
77.AlcCatb	.003813	.003799	16.1	.003813	.003799	16.1
99.AlcCatb	.00157	.001568	25.18	.00157	.001568	25.18
2._RFSMOK3	.1743	.1439	1.717	.1743	.1439	1.717
9._RFSMOK3	.004037	.004021	15.64	.004037	.004021	15.64

Table A16 Covariate distribution before and after entropy balancing (caregivers versus non-caregivers) – Analysis of physical activity, age group 65+

Treated units: 3980 total of weights: 3980
Control units: 16246 total of weights: 3980

Before: without weighting

	Caregivers (treat)			Non-caregivers (control)		
	mean	variance	skewness	mean	variance	skewness
_AGE80	72.15	25.86	.2612	73.04	27.68	.0221
2.sex	.6359	.2316	-.565	.5865	.2425	-.3512
9.sex	.0002513	.0002513	63.06	.0004924	.0004922	45.03
2._RACEGR3	.03869	.03721	4.784	.04038	.03875	4.67
3._RACEGR3	.0593	.05579	3.732	.07005	.06515	3.369
4._RACEGR3	.03693	.03558	4.911	.02918	.02833	5.595
5._RACEGR3	.04271	.0409	4.523	.04666	.04448	4.299
9._RACEGR3	.01834	.01801	7.179	.01982	.01943	6.89
2._EDUCAG	.2191	.1711	1.358	.2646	.1946	1.068
3._EDUCAG	.2962	.2085	.8926	.2665	.1955	1.056
4._EDUCAG	.4367	.2461	.2553	.4023	.2405	.3984
9._EDUCAG	.001759	.001756	23.78	.003385	.003374	17.1
2.EMPLOY1	.07136	.06628	3.33	.06285	.0589	3.603
3.EMPLOY1	.01407	.01388	8.251	.01077	.01066	9.479
4.EMPLOY1	.003518	.003506	16.77	.004801	.004778	14.33
5.EMPLOY1	.05101	.04842	4.082	.04561	.04353	4.356
6.EMPLOY1	.001256	.001255	28.16	.0006155	.0006152	40.27
7.EMPLOY1	.7121	.2051	-.9367	.7182	.2024	-.9701
8.EMPLOY1	.02739	.02664	5.792	.03946	.0379	4.731
9.EMPLOY1	.005025	.005001	14	.003939	.003924	15.84
2._INCOMG	.141	.1211	2.064	.1511	.1282	1.949
3._INCOMG	.109	.09718	2.509	.1054	.09428	2.57
4._INCOMG	.146	.1247	2.005	.1381	.119	2.098
5._INCOMG	.396	.2392	.4254	.3552	.2291	.605
9._INCOMG	.1633	.1367	1.822	.176	.145	1.702
2._CHLDCNT	.03065	.02972	5.446	.02296	.02243	6.37
3._CHLDCNT	.01206	.01192	8.94	.01016	.01005	9.771
4._CHLDCNT	.00402	.004005	15.68	.004186	.004168	15.36
5._CHLDCNT	.001759	.001756	23.78	.001724	.001721	24.03
6._CHLDCNT	.0002513	.0002513	63.06	.0004924	.0004922	45.03
9._CHLDCNT	.001759	.001756	23.78	.0008002	.0007996	35.31
2.marital	.1289	.1123	2.215	.1515	.1286	1.944
3.marital	.1844	.1504	1.627	.2924	.2069	.9129
4.marital	.01106	.01094	9.352	.01096	.01084	9.396
5.marital	.04749	.04524	4.255	.06439	.06024	3.55
6.marital	.01256	.01241	8.753	.009479	.00939	10.12
9.marital	.00402	.004005	15.68	.004432	.004412	14.92
1.menthlth1	.153	.1296	1.928	.1095	.09752	2.501
2.menthlth1	.04472	.04273	4.405	.03004	.02914	5.507
3.menthlth1	.02236	.02187	6.461	.0197	.01931	6.913
4.menthlth1	.01508	.01485	7.959	.008494	.008423	10.71
5.menthlth1	.00603	.005995	12.76	.003262	.003252	17.42
6.menthlth1	.0495	.04706	4.154	.03626	.03494	4.962
77.menthlth1	.01206	.01192	8.94	.01188	.01174	9.01
99.menthlth1	.00402	.004005	15.68	.005171	.005144	13.8
1.physhlth1	.1867	.1519	1.608	.1525	.1293	1.933
2.physhlth1	.0598	.05624	3.713	.05257	.04981	4.01
3.physhlth1	.03367	.03254	5.171	.03656	.03523	4.938
4.physhlth1	.01457	.01436	8.102	.01502	.01479	7.975
5.physhlth1	.01005	.009952	9.824	.008679	.008604	10.59
6.physhlth1	.08719	.0796	2.927	.09805	.08845	2.703
77.physhlth1	.01658	.01631	7.571	.02314	.02261	6.343

99.physhlth1	.004271	.004254	15.2	.006771	.006725	12.03
1.AlcCatb	.08693	.0794	2.932	.09276	.08416	2.808
2.AlcCatb	.01633	.01607	7.632	.0168	.01652	7.518
77.AlcCatb	.003015	.003007	18.13	.005417	.005388	13.48
99.AlcCatb	.001508	.001506	25.7	.001847	.001843	23.21
2._RFSMOK3	.0809	.07438	3.074	.07737	.07139	3.164
9._RFSMOK3	.00603	.005995	12.76	.007879	.007817	11.13

After: _uw1aPAB3 as the weighting variable

	Caregivers (treat)			Non-caregivers (control)		
	mean	variance	skewness	mean	variance	skewness
_AGE80	72.15	25.86	.2612	72.15	25.86	.2629
2.sex	.6359	.2316	-.565	.6359	.2316	-.5648
9.sex	.0002513	.0002513	63.06	.0002514	.0002514	63.05
2._RACEGR3	.03869	.03721	4.784	.0387	.0372	4.783
3._RACEGR3	.0593	.05579	3.732	.0593	.05579	3.732
4._RACEGR3	.03693	.03558	4.911	.03694	.03558	4.91
5._RACEGR3	.04271	.0409	4.523	.04272	.0409	4.523
9._RACEGR3	.01834	.01801	7.179	.01834	.01801	7.179
2._EDUCAG	.2191	.1711	1.358	.2191	.1711	1.358
3._EDUCAG	.2962	.2085	.8926	.2963	.2085	.8923
4._EDUCAG	.4367	.2461	.2553	.4367	.246	.2552
9._EDUCAG	.001759	.001756	23.78	.00176	.001757	23.78
2.EMPLOY1	.07136	.06628	3.33	.07136	.06627	3.33
3.EMPLOY1	.01407	.01388	8.251	.01407	.01387	8.251
4.EMPLOY1	.003518	.003506	16.77	.003518	.003506	16.77
5.EMPLOY1	.05101	.04842	4.082	.05101	.04841	4.081
6.EMPLOY1	.001256	.001255	28.16	.001256	.001255	28.16
7.EMPLOY1	.7121	.2051	-.9367	.712	.2051	-.9364
8.EMPLOY1	.02739	.02664	5.792	.02739	.02664	5.791
9.EMPLOY1	.005025	.005001	14	.005026	.005001	14
2._INCOMG	.141	.1211	2.064	.141	.1211	2.063
3._INCOMG	.109	.09718	2.509	.1091	.09718	2.508
4._INCOMG	.146	.1247	2.005	.146	.1247	2.005
5._INCOMG	.396	.2392	.4254	.396	.2392	.4253
9._INCOMG	.1633	.1367	1.822	.1634	.1367	1.821
2._CHLDCNT	.03065	.02972	5.446	.03066	.02972	5.445
3._CHLDCNT	.01206	.01192	8.94	.01206	.01192	8.94
4._CHLDCNT	.00402	.004005	15.68	.004021	.004005	15.68
5._CHLDCNT	.001759	.001756	23.78	.001759	.001756	23.78
6._CHLDCNT	.0002513	.0002513	63.06	.0002514	.0002513	63.05
9._CHLDCNT	.001759	.001756	23.78	.001759	.001756	23.78
2.marital	.1289	.1123	2.215	.1289	.1123	2.215
3.marital	.1844	.1504	1.627	.1845	.1505	1.627
4.marital	.01106	.01094	9.352	.01106	.01093	9.352
5.marital	.04749	.04524	4.255	.04749	.04524	4.255
6.marital	.01256	.01241	8.753	.01256	.01241	8.752
9.marital	.00402	.004005	15.68	.004021	.004005	15.68
1.menthlth1	.153	.1296	1.928	.153	.1296	1.927
2.menthlth1	.04472	.04273	4.405	.04473	.04273	4.405
3.menthlth1	.02236	.02187	6.461	.02236	.02187	6.46
4.menthlth1	.01508	.01485	7.959	.01508	.01485	7.959
5.menthlth1	.00603	.005995	12.76	.006031	.005995	12.76
6.menthlth1	.0495	.04706	4.154	.0495	.04706	4.154
77.menthlth1	.01206	.01192	8.94	.01206	.01192	8.94
99.menthlth1	.00402	.004005	15.68	.004021	.004005	15.68
1.physhlth1	.1867	.1519	1.608	.1867	.1519	1.608
2.physhlth1	.0598	.05624	3.713	.0598	.05623	3.713
3.physhlth1	.03367	.03254	5.171	.03367	.03254	5.17
4.physhlth1	.01457	.01436	8.102	.01457	.01436	8.101
5.physhlth1	.01005	.009952	9.824	.01005	.009952	9.823
6.physhlth1	.08719	.0796	2.927	.0872	.0796	2.926
77.physhlth1	.01658	.01631	7.571	.01659	.01631	7.57
99.physhlth1	.004271	.004254	15.2	.004272	.004254	15.2
1.AlcCatb	.08693	.0794	2.932	.08694	.07939	2.932
2.AlcCatb	.01633	.01607	7.632	.01633	.01607	7.632
77.AlcCatb	.003015	.003007	18.13	.003016	.003007	18.13
99.AlcCatb	.001508	.001506	25.7	.001508	.001506	25.7
2._RFSMOK3	.0809	.07438	3.074	.08091	.07437	3.074

9._RFSMOK3	.00603	.005995	12.76	.006031	.005995	12.76

Identification of differences – dementia caregivers versus non-caregivers

Table A17 Covariate distribution before and after entropy balancing (dementia caregivers versus non-caregivers) – Analysis of obesity

Treated units: 1205 total of weights: 1205
Control units: 45416 total of weights: 1205

Before: without weighting

	Dementia caregivers (treat) mean	variance	skewness	Non-caregivers (control) mean	variance	skewness
2.sex	.6622	.2239	-.6861	.539	.2485	-.1564
9.sex	.00166	.001658	24.48	.0004404	.0004402	47.62
_AGE80	59.69	190.5	-.696	55.19	302.3	-.3922
2._RACEGR3	.04647	.04435	4.309	.05082	.04824	4.09
3._RACEGR3	.08548	.07824	2.965	.08865	.08079	2.894
4._RACEGR3	.05394	.05107	3.949	.04375	.04184	4.461
5._RACEGR3	.0639	.05987	3.566	.09261	.08404	2.811
9._RACEGR3	.01909	.01874	7.029	.01803	.01771	7.244
2._EDUCAG	.2191	.1712	1.358	.2569	.1909	1.113
3._EDUCAG	.3071	.2129	.8366	.2715	.1978	1.028
4._EDUCAG	.4498	.2477	.2019	.4067	.2413	.3798
9._EDUCAG	.00166	.001658	24.48	.003281	.00327	17.37
2.EMPLOY1	.09461	.08573	2.77	.08944	.08144	2.877
3.EMPLOY1	.0249	.0243	6.099	.02129	.02084	6.632
4.EMPLOY1	.01079	.01068	9.471	.02072	.02029	6.729
5.EMPLOY1	.06307	.05914	3.595	.04853	.04618	4.202
6.EMPLOY1	.01411	.01392	8.24	.02728	.02654	5.804
7.EMPLOY1	.3519	.2282	.6204	.2989	.2095	.8788
8.EMPLOY1	.04066	.03904	4.651	.0637	.05964	3.573
9.EMPLOY1	.005809	.00578	13.01	.006914	.006866	11.9
2._INCOMG	.1245	.1091	2.275	.1316	.1143	2.18
3._INCOMG	.07967	.07338	3.105	.08327	.07634	3.017
4._INCOMG	.1212	.1066	2.322	.1179	.104	2.37
5._INCOMG	.5012	.2502	-.004979	.4491	.2474	.2047
9._INCOMG	.1195	.1053	2.346	.1403	.1206	2.071
2._CHLDCNT	.08714	.07961	2.928	.1067	.09528	2.549
3._CHLDCNT	.05394	.05107	3.949	.09197	.08351	2.824
4._CHLDCNT	.02075	.02033	6.725	.04029	.03867	4.675
5._CHLDCNT	.01079	.01068	9.471	.01603	.01577	7.707
6._CHLDCNT	.00332	.003311	17.27	.008852	.008773	10.49
9._CHLDCNT	.004979	.004959	14.07	.004492	.004472	14.82
2.marital	.1336	.1159	2.154	.137	.1182	2.111
3.marital	.08382	.07686	3.004	.1237	.1084	2.286
4.marital	.01411	.01392	8.24	.01955	.01917	6.94
5.marital	.122	.1072	2.31	.1707	.1416	1.751
6.marital	.01826	.01794	7.197	.03195	.03093	5.323
9.marital	.004979	.004959	14.07	.005086	.005061	13.91
1.menthlth1	.19	.1541	1.58	.1559	.1316	1.897
2.menthlth1	.05477	.05181	3.914	.04562	.04354	4.355
3.menthlth1	.03485	.03367	5.072	.03149	.0305	5.366
4.menthlth1	.02241	.02192	6.454	.01451	.0143	8.12
5.menthlth1	.008299	.008237	10.84	.005791	.005758	13.03
6.menthlth1	.07801	.07198	3.147	.04928	.04685	4.165
77.menthlth1	.01079	.01068	9.471	.009996	.009897	9.851
99.menthlth1	.004149	.004136	15.43	.004338	.004319	15.08
1.physhlth1	.2041	.1626	1.468	.1799	.1476	1.666
2.physhlth1	.06639	.06203	3.483	.04915	.04673	4.171
3.physhlth1	.03154	.03057	5.361	.03142	.03043	5.372
4.physhlth1	.01494	.01473	7.997	.01339	.01321	8.468
5.physhlth1	.007469	.007419	11.44	.007905	.007842	11.11
6.physhlth1	.08382	.07686	3.004	.07878	.07258	3.127
77.physhlth1	.01577	.01553	7.774	.01462	.01441	8.088
99.physhlth1	.00332	.003311	17.27	.004624	.004603	14.6
1.AlcCatb	.1436	.1231	2.033	.1483	.1263	1.979
2.AlcCatb	.03568	.03444	5.006	.04943	.04699	4.157
77.AlcCatb	.004149	.004136	15.43	.00665	.006606	12.14
99.AlcCatb	.00166	.001658	24.48	.001585	.001583	25.06
2._RFSMOK3	.127	.1109	2.241	.1246	.1091	2.274
9._RFSMOK3	.00332	.003311	17.27	.005791	.005758	13.03

After: _uw2aObesity as the weighting variable

	Dementia caregivers (treat)			Non-caregivers (control)		
	mean	variance	skewness	mean	variance	skewness
2.sex	.6622	.2239	-.6861	.662	.2238	-.685
9.sex	.00166	.001658	24.48	.001661	.001658	24.48
_AGE80	59.69	190.5	-.696	59.68	190.5	-.6939
2._RACEGR3	.04647	.04435	4.309	.04649	.04433	4.308
3._RACEGR3	.08548	.07824	2.965	.08551	.0782	2.964
4._RACEGR3	.05394	.05107	3.949	.05397	.05106	3.948
5._RACEGR3	.0639	.05987	3.566	.06393	.05984	3.565
9._RACEGR3	.01909	.01874	7.029	.0191	.01873	7.027
2._EDUCAG	.2191	.1712	1.358	.2193	.1712	1.357
3._EDUCAG	.3071	.2129	.8366	.3073	.2129	.8355
4._EDUCAG	.4498	.2477	.2019	.4499	.2475	.2015
9._EDUCAG	.00166	.001658	24.48	.001661	.001658	24.48
2.EMPLOY1	.09461	.08573	2.77	.09464	.08569	2.77
3.EMPLOY1	.0249	.0243	6.099	.02491	.02429	6.097
4.EMPLOY1	.01079	.01068	9.471	.01079	.01068	9.469
5.EMPLOY1	.06307	.05914	3.595	.0631	.05912	3.594
6.EMPLOY1	.01411	.01392	8.24	.01411	.01392	8.238
7.EMPLOY1	.3519	.2282	.6204	.3521	.2281	.6195
8.EMPLOY1	.04066	.03904	4.651	.04068	.03903	4.65
9.EMPLOY1	.005809	.00578	13.01	.005812	.005778	13
2._INCOMG	.1245	.1091	2.275	.1246	.1091	2.273
3._INCOMG	.07967	.07338	3.105	.0797	.07335	3.104
4._INCOMG	.1212	.1066	2.322	.1213	.1066	2.32
5._INCOMG	.5012	.2502	-.004979	.5012	.25	-.004968
9._INCOMG	.1195	.1053	2.346	.1196	.1053	2.344
2._CHLDCNT	.08714	.07961	2.928	.08717	.07958	2.927
3._CHLDCNT	.05394	.05107	3.949	.05397	.05106	3.948
4._CHLDCNT	.02075	.02033	6.725	.02076	.02033	6.723
5._CHLDCNT	.01079	.01068	9.471	.01079	.01068	9.469
6._CHLDCNT	.00332	.003311	17.27	.003321	.00331	17.27
9._CHLDCNT	.004979	.004959	14.07	.004982	.004957	14.06
2.marital	.1336	.1159	2.154	.1337	.1159	2.152
3.marital	.08382	.07686	3.004	.08385	.07682	3.003
4.marital	.01411	.01392	8.24	.01411	.01392	8.238
5.marital	.122	.1072	2.31	.1221	.1072	2.308
6.marital	.01826	.01794	7.197	.01827	.01793	7.195
9.marital	.004979	.004959	14.07	.004982	.004957	14.06
1.menthlth1	.19	.1541	1.58	.1902	.154	1.579
2.menthlth1	.05477	.05181	3.914	.0548	.05179	3.912
3.menthlth1	.03485	.03367	5.072	.03487	.03366	5.071
4.menthlth1	.02241	.02192	6.454	.02242	.02192	6.452
5.menthlth1	.008299	.008237	10.84	.008303	.008234	10.84
6.menthlth1	.07801	.07198	3.147	.07804	.07195	3.146
77.menthlth1	.01079	.01068	9.471	.01079	.01068	9.469
99.menthlth1	.004149	.004136	15.43	.004151	.004134	15.42
1.physhlth1	.2041	.1626	1.468	.2043	.1626	1.467
2.physhlth1	.06639	.06203	3.483	.06642	.06201	3.482
3.physhlth1	.03154	.03057	5.361	.03155	.03056	5.36
4.physhlth1	.01494	.01473	7.997	.01495	.01472	7.995
5.physhlth1	.007469	.007419	11.44	.007473	.007417	11.44
6.physhlth1	.08382	.07686	3.004	.08385	.07682	3.003
77.physhlth1	.01577	.01553	7.774	.01578	.01553	7.772
99.physhlth1	.00332	.003311	17.27	.003321	.00331	17.27
1.AlcCatb	.1436	.1231	2.033	.1437	.1231	2.031
2.AlcCatb	.03568	.03444	5.006	.0357	.03443	5.005
77.AlcCatb	.004149	.004136	15.43	.004151	.004134	15.42
99.AlcCatb	.00166	.001658	24.48	.001661	.001658	24.48
2._RFSMOK3	.127	.1109	2.241	.1271	.1109	2.239
9._RFSMOK3	.00332	.003311	17.27	.003321	.00331	17.27

Table A18 Covariate distribution before and after entropy balancing (dementia caregivers versus non-caregivers) – Analysis of Alcohol

Treated units: 1214 total of weights: 1214
Control units: 45929 total of weights: 1214

Before: without weighting

	Dementia caregivers (treat) mean	variance	skewness	Non-caregivers (control) mean	variance	skewness
2.sex	.6623	.2239	-.6862	.5381	.2486	-.1528
9.sex	.001647	.001646	24.58	.0005661	.0005658	41.99
_AGE80	59.67	190.6	-.6928	55.19	302.3	-.3926
2._RACEGR3	.04613	.04404	4.327	.0509	.04831	4.086
3._RACEGR3	.08484	.07771	2.98	.08855	.08071	2.897
4._RACEGR3	.05354	.05072	3.967	.04363	.04173	4.468
5._RACEGR3	.06343	.05945	3.582	.09293	.08429	2.804
9._RACEGR3	.01895	.0186	7.057	.01838	.01804	7.172
2._EDUCAG	.2199	.1717	1.352	.2578	.1913	1.108
3._EDUCAG	.3056	.2124	.844	.2709	.1975	1.031
4._EDUCAG	.4498	.2477	.202	.406	.2412	.3828
9._EDUCAG	.001647	.001646	24.58	.003527	.003515	16.75
2.EMPLOY1	.0972	.08782	2.72	.08946	.08146	2.877
3.EMPLOY1	.02471	.02412	6.123	.02125	.0208	6.639
4.EMPLOY1	.01071	.0106	9.508	.02097	.02053	6.687
5.EMPLOY1	.0626	.05873	3.611	.04851	.04616	4.203
6.EMPLOY1	.014	.01382	8.272	.02728	.02654	5.804
7.EMPLOY1	.3509	.228	.6248	.2989	.2096	.8786
8.EMPLOY1	.04119	.03952	4.618	.06379	.05973	3.57
9.EMPLOY1	.005766	.005738	13.06	.00725	.007198	11.62
2._INCOMG	.126	.1102	2.254	.1316	.1142	2.18
3._INCOMG	.07908	.07288	3.12	.08317	.07626	3.019
4._INCOMG	.1203	.1059	2.335	.1177	.1039	2.372
5._INCOMG	.5016	.2502	-.00659	.4471	.2472	.2128
9._INCOMG	.1194	.1053	2.347	.1427	.1223	2.044
2._CHLDCNT	.08649	.07908	2.942	.1066	.0952	2.55
3._CHLDCNT	.05437	.05145	3.931	.09188	.08344	2.826
4._CHLDCNT	.02142	.02098	6.612	.04004	.03844	4.692
5._CHLDCNT	.01071	.0106	9.508	.01592	.01566	7.736
6._CHLDCNT	.003295	.003287	17.34	.008949	.008869	10.43
9._CHLDCNT	.004942	.004922	14.12	.004855	.004832	14.25
2.marital	.1334	.1157	2.156	.1369	.1182	2.113
3.marital	.08402	.07702	2.999	.1238	.1085	2.284
4.marital	.014	.01382	8.272	.01951	.01913	6.948
5.marital	.1219	.1071	2.311	.1707	.1416	1.75
6.marital	.01812	.01781	7.225	.03201	.03098	5.318
9.marital	.004942	.004922	14.12	.005269	.005241	13.67
1.menthlth1	.1895	.1537	1.585	.1555	.1313	1.902
2.menthlth1	.05437	.05145	3.931	.04533	.04328	4.371
3.menthlth1	.03542	.03419	5.027	.03135	.03037	5.378
4.menthlth1	.02224	.02176	6.48	.01452	.01431	8.116
5.menthlth1	.008237	.008176	10.88	.005748	.005715	13.08
6.menthlth1	.07743	.07149	3.162	.04958	.04712	4.15
77.menthlth1	.01153	.01141	9.15	.01034	.01024	9.68
99.menthlth1	.004119	.004105	15.49	.00442	.0044	14.94
1.physhlth1	.2035	.1622	1.473	.1794	.1472	1.671
2.physhlth1	.0659	.06161	3.499	.04903	.04663	4.177
3.physhlth1	.0313	.03035	5.383	.03133	.03035	5.38
4.physhlth1	.01483	.01462	8.029	.01337	.01319	8.474
5.physhlth1	.007414	.007365	11.48	.007838	.007777	11.16
6.physhlth1	.0832	.07634	3.018	.07886	.07264	3.125
77.physhlth1	.01647	.01622	7.597	.01511	.01488	7.95
99.physhlth1	.003295	.003287	17.34	.004703	.004681	14.48
2._RFSMOK3	.126	.1102	2.254	.125	.1094	2.267
9._RFSMOK3	.003295	.003287	17.34	.006205	.006167	12.58
2._PAINDX1	.4217	.2441	.3169	.4569	.2481	.1731
9._PAINDX1	.03377	.03266	5.162	.03593	.03464	4.987
2._RFBMI5	.6549	.2262	-.6515	.6145	.2369	-.4704
9._RFBMI5	.05025	.04776	4.118	.05916	.05566	3.737

After: _uw2aAlcohol as the weighting variable

	Dementia caregivers (treat) mean	variance	skewness	Non-caregivers (control) mean	variance	skewness

2.sex	.6623	.2239	-.6862	.6621	.2237	-.6852
9.sex	.001647	.001646	24.58	.001648	.001646	24.57
_AGE80	59.67	190.6	-.6928	59.66	190.6	-.6904
2._RACEGR3	.04613	.04404	4.327	.04615	.04402	4.326
3._RACEGR3	.08484	.07771	2.98	.08488	.07767	2.979
4._RACEGR3	.05354	.05072	3.967	.05356	.0507	3.966
5._RACEGR3	.06343	.05945	3.582	.06345	.05943	3.582
9._RACEGR3	.01895	.0186	7.057	.01895	.0186	7.055
2._EDUCAG	.2199	.1717	1.352	.2201	.1717	1.351
3._EDUCAG	.3056	.2124	.844	.3058	.2123	.8429
4._EDUCAG	.4498	.2477	.202	.4498	.2475	.2017
9._EDUCAG	.001647	.001646	24.58	.001648	.001646	24.57
2.EMPLOY1	.0972	.08782	2.72	.09723	.08778	2.719
3.EMPLOY1	.02471	.02412	6.123	.02472	.02411	6.122
4.EMPLOY1	.01071	.0106	9.508	.01071	.0106	9.505
5.EMPLOY1	.0626	.05873	3.611	.06263	.05871	3.61
6.EMPLOY1	.014	.01382	8.272	.01401	.01381	8.27
7.EMPLOY1	.3509	.228	.6248	.3511	.2278	.6239
8.EMPLOY1	.04119	.03952	4.618	.0412	.03951	4.617
9.EMPLOY1	.005766	.005738	13.06	.005769	.005736	13.05
2._INCOMG	.126	.1102	2.254	.1262	.1102	2.252
3._INCOMG	.07908	.07288	3.12	.07911	.07285	3.119
4._INCOMG	.1203	.1059	2.335	.1204	.1059	2.333
5._INCOMG	.5016	.2502	-.00659	.5016	.25	-.006576
9._INCOMG	.1194	.1053	2.347	.1196	.1053	2.345
2._CHLDCNT	.08649	.07908	2.942	.08652	.07904	2.941
3._CHLDCNT	.05437	.05145	3.931	.05439	.05143	3.93
4._CHLDCNT	.02142	.02098	6.612	.02143	.02097	6.61
5._CHLDCNT	.01071	.0106	9.508	.01071	.0106	9.505
6._CHLDCNT	.003295	.003287	17.34	.003296	.003286	17.33
9._CHLDCNT	.004942	.004922	14.12	.004945	.00492	14.12
2.marital	.1334	.1157	2.156	.1336	.1157	2.154
3.marital	.08402	.07702	2.999	.08405	.07699	2.998
4.marital	.014	.01382	8.272	.01401	.01381	8.27
5.marital	.1219	.1071	2.311	.122	.1071	2.309
6.marital	.01812	.01781	7.225	.01813	.0178	7.223
9.marital	.004942	.004922	14.12	.004945	.00492	14.12
1.menthlth1	.1895	.1537	1.585	.1896	.1537	1.584
2.menthlth1	.05437	.05145	3.931	.05439	.05143	3.93
3.menthlth1	.03542	.03419	5.027	.03544	.03418	5.026
4.menthlth1	.02224	.02176	6.48	.02225	.02176	6.478
5.menthlth1	.008237	.008176	10.88	.008241	.008173	10.88
6.menthlth1	.07743	.07149	3.162	.07746	.07146	3.161
77.menthlth1	.01153	.01141	9.15	.01154	.0114	9.148
99.menthlth1	.004119	.004105	15.49	.004121	.004104	15.48
1.physhlth1	.2035	.1622	1.473	.2036	.1622	1.472
2.physhlth1	.0659	.06161	3.499	.06592	.06158	3.498
3.physhlth1	.0313	.03035	5.383	.03132	.03034	5.382
4.physhlth1	.01483	.01462	8.029	.01483	.01461	8.027
5.physhlth1	.007414	.007365	11.48	.007417	.007362	11.48
6.physhlth1	.0832	.07634	3.018	.08323	.0763	3.018
77.physhlth1	.01647	.01622	7.597	.01648	.01621	7.595
99.physhlth1	.003295	.003287	17.34	.003296	.003286	17.33
2._RFSMOK3	.126	.1102	2.254	.1262	.1102	2.252
9._RFSMOK3	.003295	.003287	17.34	.003296	.003286	17.33
2._PAINDX1	.4217	.2441	.3169	.4219	.2439	.3164
9._PAINDX1	.03377	.03266	5.162	.03379	.03265	5.161
2._RFBMI5	.6549	.2262	-.6515	.6546	.2261	-.6505
9._RFBMI5	.05025	.04776	4.118	.05027	.04774	4.117

Table A19 Covariate distribution before and after entropy balancing (dementia caregivers versus non-caregivers) – Analysis of Tobacco

Treated units: 1205 total of weights: 1205
Control units: 45416 total of weights: 1205

Before: without weighting

	Dementia caregivers (treat)	Non-caregivers (control)

	mean	variance	skewness	mean	variance	skewness
2.sex	.6622	.2239	-.6861	.539	.2485	-.1564
9.sex	.00166	.001658	24.48	.0004404	.0004402	47.62
_AGE80	59.69	190.5	-.696	55.19	302.3	-.3922
2._RACEGR3	.04647	.04435	4.309	.05082	.04824	4.09
3._RACEGR3	.08548	.07824	2.965	.08865	.08079	2.894
4._RACEGR3	.05394	.05107	3.949	.04375	.04184	4.461
5._RACEGR3	.0639	.05987	3.566	.09261	.08404	2.811
9._RACEGR3	.01909	.01874	7.029	.01803	.01771	7.244
2._EDUCAG	.2191	.1712	1.358	.2569	.1909	1.113
3._EDUCAG	.3071	.2129	.8366	.2715	.1978	1.028
4._EDUCAG	.4498	.2477	.2019	.4067	.2413	.3798
9._EDUCAG	.00166	.001658	24.48	.003281	.00327	17.37
2.EMPLOY1	.09461	.08573	2.77	.08944	.08144	2.877
3.EMPLOY1	.0249	.0243	6.099	.02129	.02084	6.632
4.EMPLOY1	.01079	.01068	9.471	.02072	.02029	6.729
5.EMPLOY1	.06307	.05914	3.595	.04853	.04618	4.202
6.EMPLOY1	.01411	.01392	8.24	.02728	.02654	5.804
7.EMPLOY1	.3519	.2282	.6204	.2989	.2095	.8788
8.EMPLOY1	.04066	.03904	4.651	.0637	.05964	3.573
9.EMPLOY1	.005809	.00578	13.01	.006914	.006866	11.9
2._INCOMG	.1245	.1091	2.275	.1316	.1143	2.18
3._INCOMG	.07967	.07338	3.105	.08327	.07634	3.017
4._INCOMG	.1212	.1066	2.322	.1179	.104	2.37
5._INCOMG	.5012	.2502	-.004979	.4491	.2474	.2047
9._INCOMG	.1195	.1053	2.346	.1403	.1206	2.071
2._CHLDCNT	.08714	.07961	2.928	.1067	.09528	2.549
3._CHLDCNT	.05394	.05107	3.949	.09197	.08351	2.824
4._CHLDCNT	.02075	.02033	6.725	.04029	.03867	4.675
5._CHLDCNT	.01079	.01068	9.471	.01603	.01577	7.707
6._CHLDCNT	.00332	.003311	17.27	.008852	.008773	10.49
9._CHLDCNT	.004979	.004959	14.07	.004492	.004472	14.82
2.marital	.1336	.1159	2.154	.137	.1182	2.111
3.marital	.08382	.07686	3.004	.1237	.1084	2.286
4.marital	.01411	.01392	8.24	.01955	.01917	6.94
5.marital	.122	.1072	2.31	.1707	.1416	1.751
6.marital	.01826	.01794	7.197	.03195	.03093	5.323
9.marital	.004979	.004959	14.07	.005086	.005061	13.91
1.menthlth1	.19	.1541	1.58	.1559	.1316	1.897
2.menthlth1	.05477	.05181	3.914	.04562	.04354	4.355
3.menthlth1	.03485	.03367	5.072	.03149	.0305	5.366
4.menthlth1	.02241	.02192	6.454	.01451	.0143	8.12
5.menthlth1	.008299	.008237	10.84	.005791	.005758	13.03
6.menthlth1	.07801	.07198	3.147	.04928	.04685	4.165
77.menthlth1	.01079	.01068	9.471	.009996	.009897	9.851
99.menthlth1	.004149	.004136	15.43	.004338	.004319	15.08
1.physhlth1	.2041	.1626	1.468	.1799	.1476	1.666
2.physhlth1	.06639	.06203	3.483	.04915	.04673	4.171
3.physhlth1	.03154	.03057	5.361	.03142	.03043	5.372
4.physhlth1	.01494	.01473	7.997	.01339	.01321	8.468
5.physhlth1	.007469	.007419	11.44	.007905	.007842	11.11
6.physhlth1	.08382	.07686	3.004	.07878	.07258	3.127
77.physhlth1	.01577	.01553	7.774	.01462	.01441	8.088
99.physhlth1	.00332	.003311	17.27	.004624	.004603	14.6
1.AlcCatb	.1436	.1231	2.033	.1483	.1263	1.979
2.AlcCatb	.03568	.03444	5.006	.04943	.04699	4.157
77.AlcCatb	.004149	.004136	15.43	.00665	.006606	12.14
99.AlcCatb	.00166	.001658	24.48	.001585	.001583	25.06
2._PAINDX1	.4207	.2439	.3211	.4563	.2481	.1754
9._PAINDX1	.03402	.03289	5.141	.03494	.03372	5.065
2._RFBMI5	.6556	.226	-.6549	.6158	.2366	-.476
9._RFBMI5	.05062	.0481	4.1	.05802	.05465	3.781

After: _uw2aTobacco as the weighting variable

	mean	Dementia caregivers (treat) variance	skewness	mean	Non-caregivers (control) variance	skewness
2.sex	.6622	.2239	-.6861	.662	.2238	-.6851
9.sex	.00166	.001658	24.48	.001661	.001658	24.48
_AGE80	59.69	190.5	-.696	59.68	190.4	-.6936

2._RACEGR3	.04647	.04435	4.309	.04649	.04433	4.308
3._RACEGR3	.08548	.07824	2.965	.08551	.0782	2.964
4._RACEGR3	.05394	.05107	3.949	.05396	.05105	3.948
5._RACEGR3	.0639	.05987	3.566	.06393	.05984	3.565
9._RACEGR3	.01909	.01874	7.029	.0191	.01873	7.028
2._EDUCAG	.2191	.1712	1.358	.2193	.1712	1.357
3._EDUCAG	.3071	.2129	.8366	.3073	.2129	.8355
4._EDUCAG	.4498	.2477	.2019	.4499	.2475	.2015
9._EDUCAG	.00166	.001658	24.48	.001661	.001658	24.48
2.EMPLOY1	.09461	.08573	2.77	.09464	.08569	2.77
3.EMPLOY1	.0249	.0243	6.099	.02491	.02429	6.097
4.EMPLOY1	.01079	.01068	9.471	.01079	.01068	9.469
5.EMPLOY1	.06307	.05914	3.595	.0631	.05912	3.594
6.EMPLOY1	.01411	.01392	8.24	.01411	.01392	8.238
7.EMPLOY1	.3519	.2282	.6204	.3521	.2281	.6195
8.EMPLOY1	.04066	.03904	4.651	.04068	.03903	4.65
9.EMPLOY1	.005809	.00578	13.01	.005812	.005778	13
2._INCOMG	.1245	.1091	2.275	.1246	.1091	2.273
3._INCOMG	.07967	.07338	3.105	.0797	.07335	3.104
4._INCOMG	.1212	.1066	2.322	.1213	.1066	2.32
5._INCOMG	.5012	.2502	-.004979	.5012	.25	-.004969
9._INCOMG	.1195	.1053	2.346	.1196	.1053	2.344
2._CHLDCNT	.08714	.07961	2.928	.08717	.07957	2.927
3._CHLDCNT	.05394	.05107	3.949	.05396	.05105	3.948
4._CHLDCNT	.02075	.02033	6.725	.02076	.02033	6.723
5._CHLDCNT	.01079	.01068	9.471	.01079	.01068	9.469
6._CHLDCNT	.00332	.003311	17.27	.003321	.00331	17.27
9._CHLDCNT	.004979	.004959	14.07	.004982	.004957	14.06
2.marital	.1336	.1159	2.154	.1337	.1159	2.152
3.marital	.08382	.07686	3.004	.08385	.07682	3.003
4.marital	.01411	.01392	8.24	.01411	.01392	8.238
5.marital	.122	.1072	2.31	.1221	.1072	2.308
6.marital	.01826	.01794	7.197	.01827	.01793	7.195
9.marital	.004979	.004959	14.07	.004982	.004957	14.06
1.menthlth1	.19	.1541	1.58	.1902	.154	1.579
2.menthlth1	.05477	.05181	3.914	.05479	.05179	3.913
3.menthlth1	.03485	.03367	5.072	.03487	.03366	5.071
4.menthlth1	.02241	.02192	6.454	.02242	.02191	6.452
5.menthlth1	.008299	.008237	10.84	.008303	.008234	10.84
6.menthlth1	.07801	.07198	3.147	.07804	.07195	3.146
77.menthlth1	.01079	.01068	9.471	.01079	.01068	9.469
99.menthlth1	.004149	.004136	15.43	.004151	.004134	15.42
1.physhlth1	.2041	.1626	1.468	.2043	.1626	1.467
2.physhlth1	.06639	.06203	3.483	.06642	.06201	3.482
3.physhlth1	.03154	.03057	5.361	.03155	.03055	5.36
4.physhlth1	.01494	.01473	7.997	.01494	.01472	7.996
5.physhlth1	.007469	.007419	11.44	.007472	.007417	11.44
6.physhlth1	.08382	.07686	3.004	.08385	.07682	3.003
77.physhlth1	.01577	.01553	7.774	.01577	.01553	7.772
99.physhlth1	.00332	.003311	17.27	.003321	.00331	17.27
1.AlcCatb	.1436	.1231	2.033	.1437	.1231	2.031
2.AlcCatb	.03568	.03444	5.006	.0357	.03443	5.005
77.AlcCatb	.004149	.004136	15.43	.004151	.004134	15.42
99.AlcCatb	.00166	.001658	24.48	.001661	.001658	24.48
2._PAINDX1	.4207	.2439	.3211	.4209	.2437	.3206
9._PAINDX1	.03402	.03289	5.141	.03404	.03288	5.139
2._RFBMI5	.6556	.226	-.6549	.6554	.2259	-.6539
9._RFBMI5	.05062	.0481	4.1	.05064	.04808	4.099

Table A20 Covariate distribution before and after entropy balancing (dementia caregivers versus non-caregivers) – Analysis of Physical Activity

Treated units: 1205 total of weights: 1205
Control units: 45416 total of weights: 1205

Before: without weighting

	Dementia caregivers (treat)			Non-caregivers (control)		
	mean	variance	skewness	mean	variance	skewness

2.sex	.6622	.2239	-.6861	.539	.2485	-.1564
9.sex	.00166	.001658	24.48	.0004404	.0004402	47.62
_AGE80	59.69	190.5	-.696	55.19	302.3	-.3922
2._RACEGR3	.04647	.04435	4.309	.05082	.04824	4.09
3._RACEGR3	.08548	.07824	2.965	.08865	.08079	2.894
4._RACEGR3	.05394	.05107	3.949	.04375	.04184	4.461
5._RACEGR3	.0639	.05987	3.566	.09261	.08404	2.811
9._RACEGR3	.01909	.01874	7.029	.01803	.01771	7.244
2._EDUCAG	.2191	.1712	1.358	.2569	.1909	1.113
3._EDUCAG	.3071	.2129	.8366	.2715	.1978	1.028
4._EDUCAG	.4498	.2477	.2019	.4067	.2413	.3798
9._EDUCAG	.00166	.001658	24.48	.003281	.00327	17.37
2.EMPLOY1	.09461	.08573	2.77	.08944	.08144	2.877
3.EMPLOY1	.0249	.0243	6.099	.02129	.02084	6.632
4.EMPLOY1	.01079	.01068	9.471	.02072	.02029	6.729
5.EMPLOY1	.06307	.05914	3.595	.04853	.04618	4.202
6.EMPLOY1	.01411	.01392	8.24	.02728	.02654	5.804
7.EMPLOY1	.3519	.2282	.6204	.2989	.2095	.8788
8.EMPLOY1	.04066	.03904	4.651	.0637	.05964	3.573
9.EMPLOY1	.005809	.00578	13.01	.006914	.006866	11.9
2._INCOMG	.1245	.1091	2.275	.1316	.1143	2.18
3._INCOMG	.07967	.07338	3.105	.08327	.07634	3.017
4._INCOMG	.1212	.1066	2.322	.1179	.104	2.37
5._INCOMG	.5012	.2502	-.004979	.4491	.2474	.2047
9._INCOMG	.1195	.1053	2.346	.1403	.1206	2.071
2._CHLDCNT	.08714	.07961	2.928	.1067	.09528	2.549
3._CHLDCNT	.05394	.05107	3.949	.09197	.08351	2.824
4._CHLDCNT	.02075	.02033	6.725	.04029	.03867	4.675
5._CHLDCNT	.01079	.01068	9.471	.01603	.01577	7.707
6._CHLDCNT	.00332	.003311	17.27	.008852	.008773	10.49
9._CHLDCNT	.004979	.004959	14.07	.004492	.004472	14.82
2.marital	.1336	.1159	2.154	.137	.1182	2.111
3.marital	.08382	.07686	3.004	.1237	.1084	2.286
4.marital	.01411	.01392	8.24	.01955	.01917	6.94
5.marital	.122	.1072	2.31	.1707	.1416	1.751
6.marital	.01826	.01794	7.197	.03195	.03093	5.323
9.marital	.004979	.004959	14.07	.005086	.005061	13.91
1.menthlth1	.19	.1541	1.58	.1559	.1316	1.897
2.menthlth1	.05477	.05181	3.914	.04562	.04354	4.355
3.menthlth1	.03485	.03367	5.072	.03149	.0305	5.366
4.menthlth1	.02241	.02192	6.454	.01451	.0143	8.12
5.menthlth1	.008299	.008237	10.84	.005791	.005758	13.03
6.menthlth1	.07801	.07198	3.147	.04928	.04685	4.165
77.menthlth1	.01079	.01068	9.471	.009996	.009897	9.851
99.menthlth1	.004149	.004136	15.43	.004338	.004319	15.08
1.physhlth1	.2041	.1626	1.468	.1799	.1476	1.666
2.physhlth1	.06639	.06203	3.483	.04915	.04673	4.171
3.physhlth1	.03154	.03057	5.361	.03142	.03043	5.372
4.physhlth1	.01494	.01473	7.997	.01339	.01321	8.468
5.physhlth1	.007469	.007419	11.44	.007905	.007842	11.11
6.physhlth1	.08382	.07686	3.004	.07878	.07258	3.127
77.physhlth1	.01577	.01553	7.774	.01462	.01441	8.088
99.physhlth1	.00332	.003311	17.27	.004624	.004603	14.6
1.AlcCatb	.1436	.1231	2.033	.1483	.1263	1.979
2.AlcCatb	.03568	.03444	5.006	.04943	.04699	4.157
77.AlcCatb	.004149	.004136	15.43	.00665	.006606	12.14
99.AlcCatb	.00166	.001658	24.48	.001585	.001583	25.06
2._RFSMOK3	.127	.1109	2.241	.1246	.1091	2.274
9._RFSMOK3	.00332	.003311	17.27	.005791	.005758	13.03

After: _uw2aPA as the weighting variable

	Dementia caregivers (treat)			Non-caregivers (control)		
	mean	variance	skewness	mean	variance	skewness
2.sex	.6622	.2239	-.6861	.662	.2238	-.685
9.sex	.00166	.001658	24.48	.001661	.001658	24.48
_AGE80	59.69	190.5	-.696	59.68	190.5	-.6939
2._RACEGR3	.04647	.04435	4.309	.04649	.04433	4.308
3._RACEGR3	.08548	.07824	2.965	.08551	.0782	2.964
4._RACEGR3	.05394	.05107	3.949	.05397	.05106	3.948

5._RACEGR3	.0639	.05987	3.566	.06393	.05984	3.565
9._RACEGR3	.01909	.01874	7.029	.0191	.01873	7.027
2._EDUCAG	.2191	.1712	1.358	.2193	.1712	1.357
3._EDUCAG	.3071	.2129	.8366	.3073	.2129	.8355
4._EDUCAG	.4498	.2477	.2019	.4499	.2475	.2015
9._EDUCAG	.00166	.001658	24.48	.001661	.001658	24.48
2.EMPLOY1	.09461	.08573	2.77	.09464	.08569	2.77
3.EMPLOY1	.0249	.0243	6.099	.02491	.02429	6.097
4.EMPLOY1	.01079	.01068	9.471	.01079	.01068	9.469
5.EMPLOY1	.06307	.05914	3.595	.0631	.05912	3.594
6.EMPLOY1	.01411	.01392	8.24	.01411	.01392	8.238
7.EMPLOY1	.3519	.2282	.6204	.3521	.2281	.6195
8.EMPLOY1	.04066	.03904	4.651	.04068	.03903	4.65
9.EMPLOY1	.005809	.00578	13.01	.005812	.005778	13
2._INCOMG	.1245	.1091	2.275	.1246	.1091	2.273
3._INCOMG	.07967	.07338	3.105	.0797	.07335	3.104
4._INCOMG	.1212	.1066	2.322	.1213	.1066	2.32
5._INCOMG	.5012	.2502	-.004979	.5012	.25	-.004968
9._INCOMG	.1195	.1053	2.346	.1196	.1053	2.344
2._CHLDCNT	.08714	.07961	2.928	.08717	.07958	2.927
3._CHLDCNT	.05394	.05107	3.949	.05397	.05106	3.948
4._CHLDCNT	.02075	.02033	6.725	.02076	.02033	6.723
5._CHLDCNT	.01079	.01068	9.471	.01079	.01068	9.469
6._CHLDCNT	.00332	.003311	17.27	.003321	.00331	17.27
9._CHLDCNT	.004979	.004959	14.07	.004982	.004957	14.06
2.marital	.1336	.1159	2.154	.1337	.1159	2.152
3.marital	.08382	.07686	3.004	.08385	.07682	3.003
4.marital	.01411	.01392	8.24	.01411	.01392	8.238
5.marital	.122	.1072	2.31	.1221	.1072	2.308
6.marital	.01826	.01794	7.197	.01827	.01793	7.195
9.marital	.004979	.004959	14.07	.004982	.004957	14.06
1.menthlth1	.19	.1541	1.58	.1902	.154	1.579
2.menthlth1	.05477	.05181	3.914	.0548	.05179	3.912
3.menthlth1	.03485	.03367	5.072	.03487	.03366	5.071
4.menthlth1	.02241	.02192	6.454	.02242	.02192	6.452
5.menthlth1	.008299	.008237	10.84	.008303	.008234	10.84
6.menthlth1	.07801	.07198	3.147	.07804	.07195	3.146
77.menthlth1	.01079	.01068	9.471	.01079	.01068	9.469
99.menthlth1	.004149	.004136	15.43	.004151	.004134	15.42
1.physhlth1	.2041	.1626	1.468	.2043	.1626	1.467
2.physhlth1	.06639	.06203	3.483	.06642	.06201	3.482
3.physhlth1	.03154	.03057	5.361	.03155	.03056	5.36
4.physhlth1	.01494	.01473	7.997	.01495	.01472	7.995
5.physhlth1	.007469	.007419	11.44	.007473	.007417	11.44
6.physhlth1	.08382	.07686	3.004	.08385	.07682	3.003
77.physhlth1	.01577	.01553	7.774	.01578	.01553	7.772
99.physhlth1	.00332	.003311	17.27	.003321	.00331	17.27
1.AlcCatb	.1436	.1231	2.033	.1437	.1231	2.031
2.AlcCatb	.03568	.03444	5.006	.0357	.03443	5.005
77.AlcCatb	.004149	.004136	15.43	.004151	.004134	15.42
99.AlcCatb	.00166	.001658	24.48	.001661	.001658	24.48
2._RFSMOK3	.127	.1109	2.241	.1271	.1109	2.239
9._RFSMOK3	.00332	.003311	17.27	.003321	.00331	17.27
